# Carbonyl derivatization tunes triplet-state aromaticity and dynamics in fused-ring cyclic amides

**DOI:** 10.1039/d6sc05784e

**Published:** 2026-08-10

**Authors:** Dexter J. Gordon, Damon M. de Clercq, Ben P. Carwithen, Adrian Mena, Max R. Bonengel, Alexander J. Baldacchino, Mohan Bhadbhade, Ruoming Tian, Dane R. McCamey, Timothy W. Schmidt, Martin D. Peeks

**Affiliations:** a School of Chemistry, UNSW Sydney NSW 2052 Australia m.peeks@unsw.edu.au; b School of Physics, UNSW Sydney NSW 2052 Australia; c Mark Wainwright Analytical Centre, UNSW Sydney NSW 2052 Australia

## Abstract

Singlet fission (SF) and triplet–triplet annihilation upconversion (TTA-UC) are photophysical processes which, respectively, result in the division of one high energy singlet state into two lower-energy triplet states, and *vice versa*. These processes are components of strategies to overcome the efficiency limits of photovoltaic devices, by funneling more energy into silicon solar cells. Molecules featuring a fused π-conjugated ring system interrupted by amide groups have recently attracted interest for singlet fission owing to their greater stability than acene-based chromophores. While aromaticity has been regularly used to design chromophores for both SF and TTA-UC and rationalize results, debate continues about the relevance and application of the concept. Here, we describe the spectroscopic properties of novel derivatives of a 5-6-5 fused-ring chromophore with a central 8π 1,4-diacyl-1,4-dihydropyrazine ring. Density functional theory calculations suggest that the parent chromophore does not meet the key thermodynamic criterion for SF, *i.e. E*(S_1_) ≥ 2*E*(T_1_), but that modifying the carbonyl groups *via* microwave-assisted thionation and subsequent silver(i)-promoted condensation will substantially modulate the *E*(T_1_)/*E*(S_1_) ratio. Through photophysical measurements including time-resolved absorption and magneto-photoluminescence, we demonstrate that intersystem crossing followed by triplet–triplet annihilation occurs upon excitation of a dicyanomethylene-substituted derivative. We propose an explanation for these observations in terms of Mandado's (2*n* + 1) aromaticity rule, which provides guidance for future designs of multiexcitonic chromophore systems based on the cyclic amide motif.

## Abstract 2

The Hückel and Mandado rules explain the emergence of triplet–triplet annihilation in a functionalized amide-containing chromophore.

## Graphical abstract

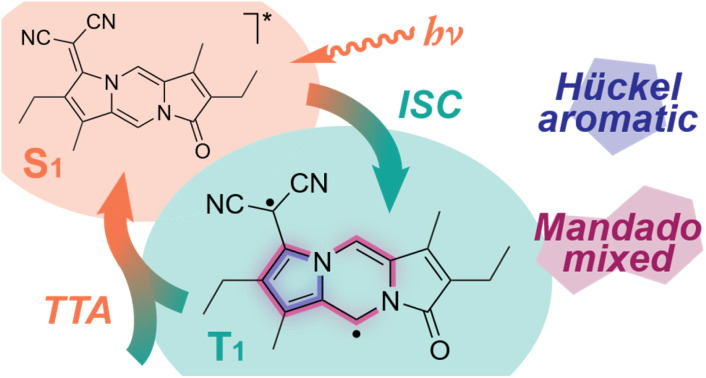

## Introduction

**Fig. 1:**
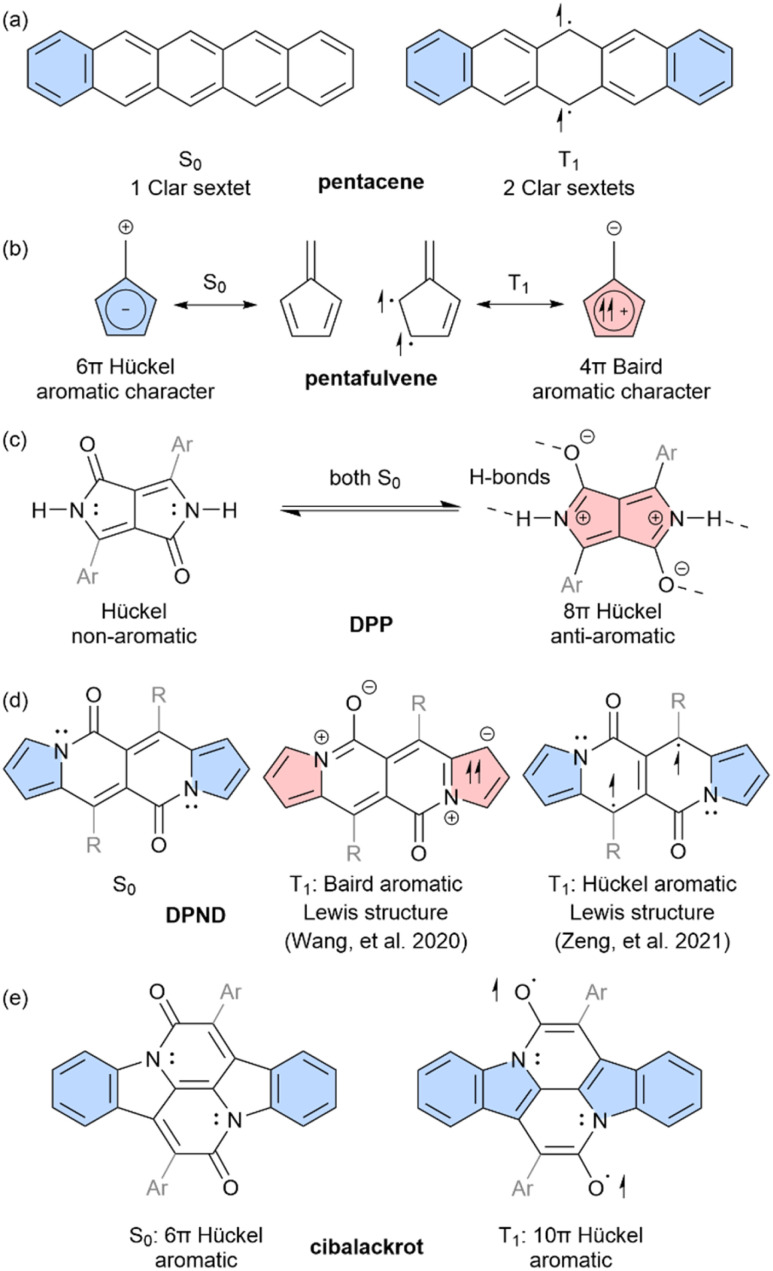
Some approaches to lowering *E*(T_1_) in organic π-conjugated molecules: (a) pentacene gains a Clar sextet in its open-shell resonance structure,^7^ while (b) pentafulvene is an aromatic chameleon.^12^ (c) DPP has enhanced Hückel antiaromaticity in hydrogen-bonded environments due to the resonance of amides;^18^ (d) the Baird aromaticity of DPND based on a similar resonance has been questioned.^14,19,20^ (e) In cibalackrot-type compounds the triplet state is considered Hückel aromatic in character.^20^ R = C_6_H_13_, Ar = aryl groups.

Conventional silicon photovoltaic cells have a theoretical maximum efficiency of around 30%.^1^ This limit arises in part because excess photon energy above the bandgap is lost as heat, and sub-bandgap photons are not absorbed. One mechanism to more efficiently harvest high energy photons is singlet fission (SF), through which a photoexcited singlet excited state is converted to a pair of triplet states *via* a spin-correlated triplet pair state.^2^ The reverse process, triplet–triplet annihilation upconversion (TTA-UC) can be used to harvest photons below the bandgap, by converting two lower-energy triplets into one high-energy singlet.^3^ For SF to be exothermic, the energy of the first excited singlet state must be at least double that of the first triplet state, *i.e. E*(S_1_) ≥ 2*E*(T_1_),^4^ and the reverse for TTA-UC; several recent studies have exploited aromaticity rules to identify potential SF chromophores that would meet this energy criterion.^5^ For example, pentacene has near quantitative SF yield,^4^ and the low energy of its T_1_ state can be explained using Clar's rule, which states that a polycyclic aromatic hydrocarbon becomes more stable as the number of aromatic π-sextets in the Kekulé resonance structure increases.^6^ Pentacene gains an additional π-sextet when a diradical resonance structure is drawn, as shown in Fig. 1a.^7^

Another approach to engineer excited-state energies of organic chromophores involves exploiting excited-state aromaticity, described by Baird's rule.^8^ While Hückel's rule states that a molecule with a planar, conjugated π-system containing (4*n* + 2) π-electrons is aromatic, and with 4*n* π-electrons is antiaromatic, Baird's rule inverts this relationship in the first excited triplet state of (π–π*) character.^8–10^ Baird aromaticity (4*n* π-electrons) is therefore a favorable design principle for preparing π-systems with low-lying triplet states suitable for SF. However, the same 4*n* π-electron configuration ordinarily leads to Hückel antiaromaticity in the ground state, resulting in chemical instability.^5^ A range of molecules for which both (4*n* + 2) and 4*n* π-electron configurations can be drawn present an exception: these aromatic chameleons are excited-state aromatic molecules that are not antiaromatic in their ground states. Pentafulvene (Fig. 1b) is one example: this molecule has Hückel aromatic character in S_0_ and Baird aromatic character in T_1_.^11,12^

Several recently-identified SF candidates contain fused π-conjugated rings interrupted by amide groups. Examples include derivatives of diketopyrrolopyrrole (DPP),^13^ dipyrrolonaphthyridinedione (DPND),^14,15^ cibalackrot,^16^ and perylene diimides (PDI).^17^ The emergence of a Hückel antiaromatic (and thus Baird aromatic) π-electron configuration in DPP can be associated with amide–iminol tautomerism, as occurs in the ground-state during hydrogen bonding,^18^ which promotes an 8 π-electron structure (Fig. 1c). Wang and co-workers drew a similar structure to explain the SF capability of DPND (Fig. 1d),^14^ though this description has been disputed, favoring a symmetric diradical structure.^19,20^ Not every excited state exhibits Baird aromaticity; in cibalackrot, the triplet state diradicals are located outside the π-conjugated ring system, and so Baird's rule does not apply and each highlighted ring in Fig. 1e is considered 10-π Hückel aromatic.^20^

**Fig. 2:**
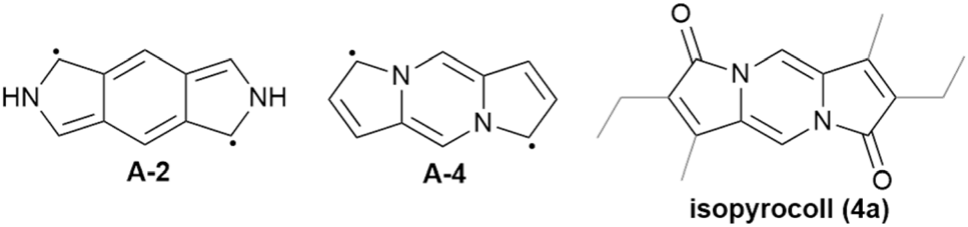
A-2 and A-4 are aza-*s*-indacene structures identified by Nagami and co-workers that are theoretically capable of SF.^21^ Isopyrocoll, 4a, was discovered by Plieninger and co-workers in 1968.^22^

Our work was inspired by theoretical studies of aza-*s*-indacene dimers, which showed that compounds A-2 and A-4, in Fig. 2, were capable of exothermic SF.^21^ While the diradical nature of these compounds as drawn would render their synthesis a serious challenge, attaching carbonyl groups at the radical sites of A-4 gives access to unsaturated lactam 4a, which was first reported as a by-product in a study of bile pigments in the 1960s.^22^ Compound 4a can be synthesized in two steps from 3-ethyl-4-methyl-3-pyrrolin-2-one,^22,23^ which is an industrially important feedstock for the diabetes drug glimepiride.^24^ Despite its short synthetic route, the photophysical properties of this unique π-system have not been previously explored. We refer to this scaffold as isopyrocoll, reflecting its structural isomerism with pyrocoll.^25^

## Results and discussion

### Synthesis of isopyrocoll

**Scheme 1:**
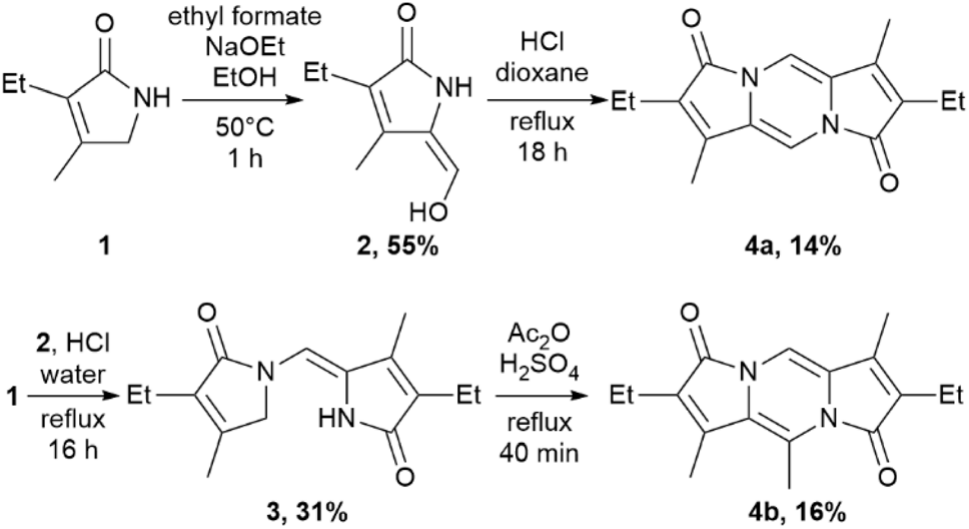
Syntheses of 4a and 4b as reproduced from literature.^22,23^

Enol 2 was synthesized according to the literature procedure.^23^ The 3,4-dimethyl analogue of enol 2 was originally thought to be isolable as the keto form,^23^ but recharacterization identified it as the enol,^26^ and the later literature agreed that 2 is an enol;^22^ our spectroscopic data are consistent with the enol structure. Since 4a had only been reported as a by-product of the synthesis of open dimer 3 from a mixture of enol 2 and pyrrolinone 1, and without an experimental yield,^22^ we attempted to optimize this final reaction. We could not exceed the modest yield shown in Scheme 1 despite testing a range of conditions. The results of the optimization are shown in SI Table S1 (additional comments are provided in SI Section S2.11). We confirmed the structure of 4a by X-ray crystallography, which also revealed that isopyrocoll crystallizes in a slip-stacked configuration with a distance between planes of 3.4 Å (SI Section S4.2).

### Modification of carbonyl groups

Our initial computational studies on isopyrocoll were conducted at the ωB97XD/def2-SVP level of theory,^27,28^ as range-separated functionals can provide a reasonably accurate description of the electronic properties of π-conjugated molecules.^29^ The calculations suggested SF in isopyrocoll 4a would be endothermic, with a calculated singlet–triplet energy ratio (*E*(S_1_)/*E*(T_1_)) of 1.79. Transient absorption (TA) spectroscopy of thin films of 4a (see SI Fig. S5) showed negligible yield of long-lived triplet states. These results led us to explore means of derivatizing 4a.

**Scheme 2:**
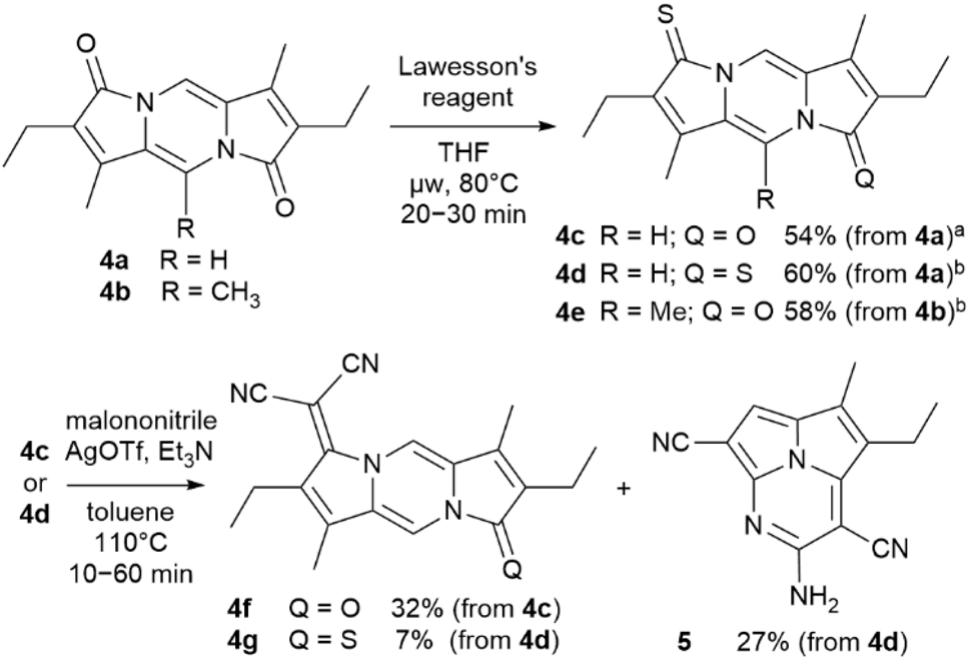
Thionation and subsequent malononitrile condensation of isopyrocoll derivatives; ^*a*^using 3 eq. Lawesson's reagent; ^*b*^using 5 eq. Lawesson's reagent.

We first attempted to derivatize 4a by substitution on the central ring with electrophilic reagents such as *N*-bromosuccinimide and SelectFluor. However, these reactions invariably gave complex mixtures of products. Attempts to lithiate the central ring using lithium diisopropylamide or *n*-butyllithium were also unsuccessful. We next identified the carbonyl groups as an alternative target for functionalization. Thionation with Lawesson's reagent was successful under microwave heating^30,31^ yielding thioamide derivatives 4c–e (see SI Table S2 for optimization details). The reaction did not proceed with conventional heating (reflux, THF). The thiocarbonyl group offers a useful chemical handle for an otherwise stubbornly unreactive framework. Guided by computational results we hoped to install dicyanomethylene groups *via* a Knoevenagel reaction. We first attempted to activate the thiocarbonyl group using methyl iodide to yield a methylthioiminium salt, followed by Knoevenagel condensation of malononitrile, as reported elsewhere on pyrrolinone- and pyridone-fused polycyclic aromatic hydrocarbons.^32,33^ This approach yielded products too unstable to be characterized, presumably because the methylthioiminium intermediate bears a central 12-p antiaromatic ring system, analogous to diaza-*s*-indacenes.^34^ Instead, we adapted a literature procedure that used silver(i) salts to promote the one-pot Knoevenagel condensation,^35,36^ affording dicyanomethylene derivatives 4f and 4g from thionated compounds 4c and 4d respectively (Scheme 2).

### Steady-state spectroscopy

**Fig. 3:**
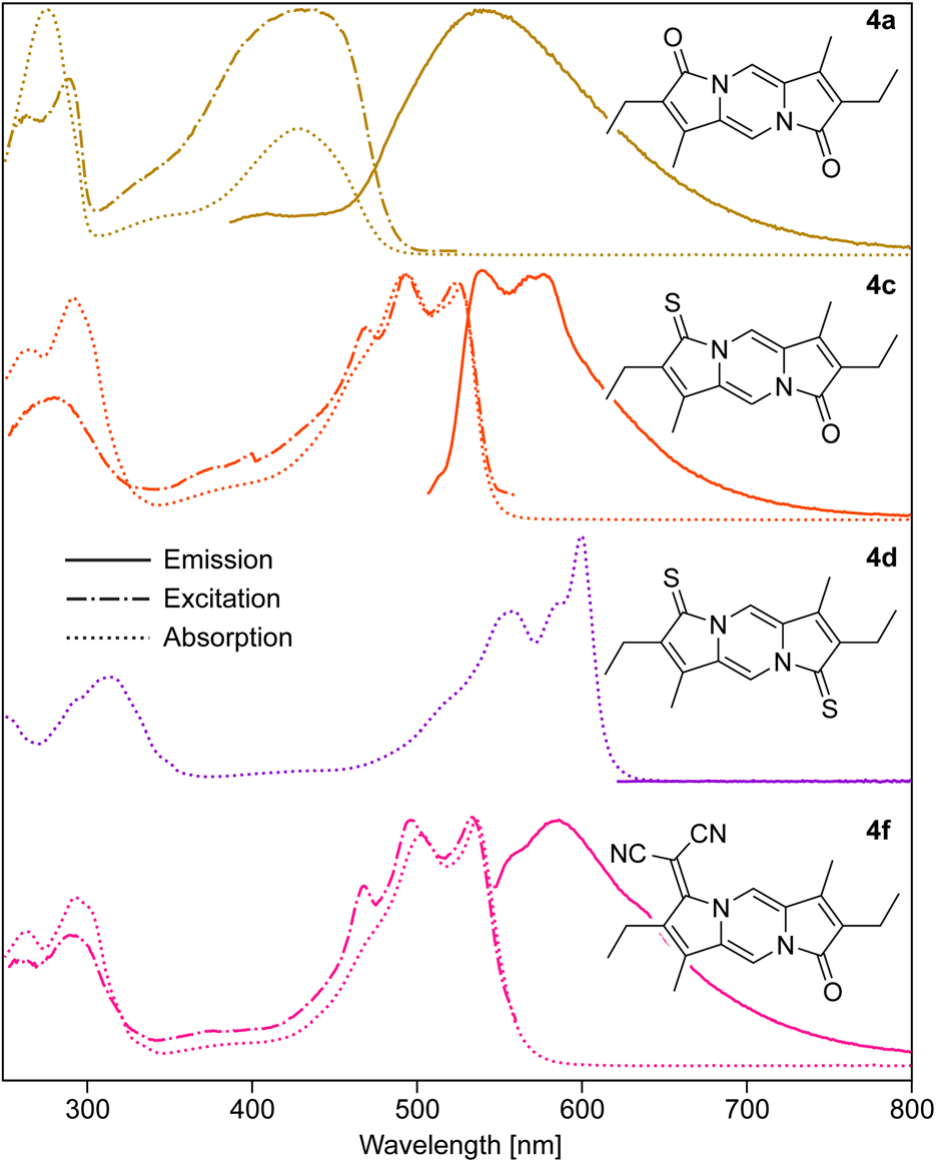
Absorption, photoluminescence emission and excitation spectra of 4a, 4c, 4d and 4f in dichloromethane. 4a: *λ*_ex_ = 365 nm, *λ*_em_ = 545 nm. 4c: *λ*_ex_ = 505 nm, *λ*_em_ = 577 nm. 4d: *λ*_ex_ = 600 nm. 4f: *λ*_ex_ = 535 nm, *λ*_em_ = 580 nm.

Compounds 4a, 4c, 4d and 4f were selected for spectroscopic analysis to investigate the effect of substituting one or more of the carbonyl groups with thiocarbonyl or dicyanomethylene. Replacement of each carbonyl group with thiocarbonyl or dicyanomethylene results in a bathochromic shift of the UV-visible absorption spectrum (Fig. 3). Fine vibronic structure is also apparent in the absorption spectra of the modified compounds, compared to the broad peak observed in 4a. Photoluminescence (PL) results, shown in Table 1, show that the PL quantum yield (PLQY) reduces when the carbonyl groups are modified. In thiocarbonyl compounds, sulfur can induce a heavy atom effect that allows intersystem crossing (ISC) to generate triplet states which can then be quenched by another ground-state molecule^37^ or by dissolved oxygen.^38^ Where ISC competes with fluorescence, a decrease in observed PLQY is expected as ISC becomes more favorable. A decrease in PLQY is also expected when SF competes with fluorescence.^4^

**Table 1:** UV-visible absorption and fluorescence emission wavelengths, molar absorption coefficients, TCSPC fluorescence lifetimes and quantum yields for 4a, 4c, 4d and 4f, *c.* 5–20 µM in dichloromethane. Molar absorption coefficients were measured at the wavelength *λ*_abs_ indicated. Errors are shown as the standard deviation of the mean

	*λ*_abs_ [nm]	*λ*_em_ [nm]	*ε* [M^−1^ cm^−1^]	*τ* [ns]	PLQY_obs_ [%]
4a	430	540	1.7 ± 0.1 × 10^4^	0.24	1.2 ± 0.2
4c	526	580	2.5 ± 0.2 × 10^4^	0.19	0.2 ± 0.1
4d	600	n.d.	6.0 ± 0.3 × 10^4^	n.d.	n.d.
4f	536	585	2.5 ± 0.1 × 10^4^	n.d.	0.8 ± 0.3

**Fig. 4:**
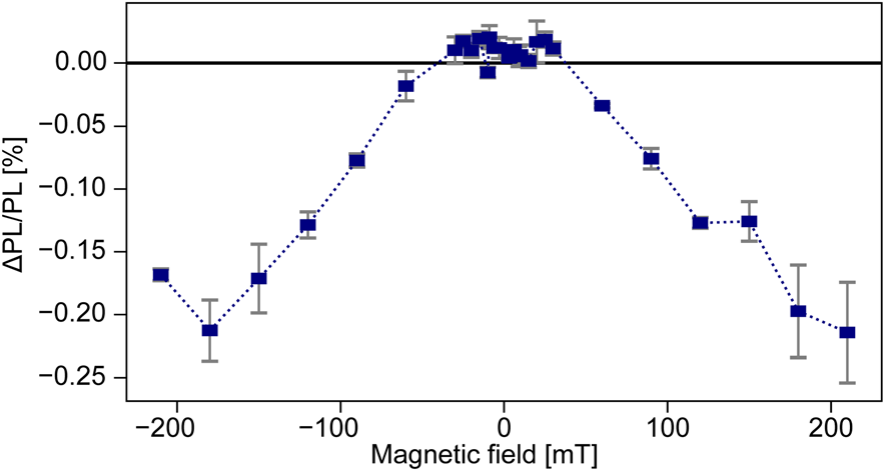
MPL measurements on a thermally evaporated thin film sample of 4f, with an excitation wavelength of 520 nm and an emission range between 550 and 900 nm.

To evaluate whether SF occurs in a thin film of the dicyanomethylene-substituted 4f, its magneto-photoluminescence (MPL) was measured. SF shows a magnetic field dependence due to the impact of the magnetic field on the spin mixing of the weakly coupled triplet pair states. At high field strengths, the number of triplet pair states with singlet character decreases from three to two, slowing the rate of SF and increasing the fluorescence, leading to a characteristic “W”-shaped MPL curve.^39^ However, as shown in Fig. 4, 4f gave an “M” curve often characteristic of the inverse process-triplet–triplet annihilation (TTA),^39^ though we cannot exclude other spin processes such as a triplet–doublet interaction. The MPL effect in the solution state, shown in SI Fig. S2, was extremely weak. These results led us to investigate the time-resolved spectroscopy of 4a, 4c, 4d and 4f.

### Time-resolved spectroscopy

**Fig. 5:**
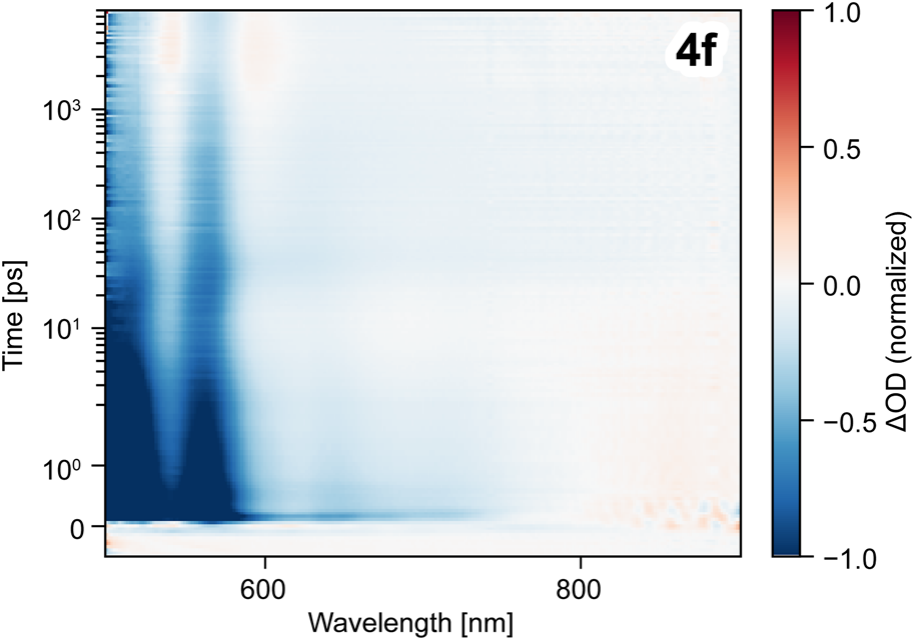
fsTA spectra for thin film of 4f. Full-size fsTA spectra for thin films of 4a, 4c, 4d and 4f are given in SI Fig. S5.

Solution transient absorption (TA) data are presented in SI Fig. S3 and S4. Consistent with our earlier assumptions, femtosecond TA (fsTA) spectroscopy shows that the initially-formed excited state of isopyrocoll 4a decays to the ground state (*τ* = 400 ps). Thionated derivatives 4c and 4d quickly (2–3 ps) convert, following photoexcitation, to a state which persists beyond the duration of the experiment (>4 ns). Solution-phase nanosecond TA (nsTA) experiments on 4c and 4d (SI Fig. S7) revealed that these states are long-lived (4c*τ* = 3.9 µs, 4d*τ* = 3.0 µs), which is consistent with a T_1_ assignment given the propensity of thioketones to undergo ISC.^37^ However, analysis of the solution fsTA spectra of the dicyanomethylene derivative 4f (SI Fig. S3) revealed interesting excited-state behavior. Initially, the S_1_ state is populated, as the species-associated spectrum (SAS) given in SI Fig. S4 shows ground-state bleach (GSB) and stimulated emission (SE) features matching the steady-state absorption and emission. Within a few picoseconds (*τ* ≈ 2 ps), a second distinct spectral component is populated and rapidly quenched (*τ* ≈ 7 ps). Based on the similarity of the SAS with that of the assigned triplet in 4c, we tentatively assign this state as a short-lived triplet, which rapidly decays *via* non-radiative pathways. We speculate that this decay could occur by rotation of, or charge transfer to, the dicyanomethylene group. fsTA spectroscopy of thin films of 4a, 4c, 4d and 4f, prepared by thermal evaporation, uncovered further interesting excited-state behavior (SI Fig. S5 and S6). As with our solution-phase experiments, thin films of 4c and 4d produced transient species that persisted beyond the duration of the fsTA experiments (>4 ns). Curiously, 4f also produced a long-lived transient species (Fig. 5), which we investigated further using nsTA spectroscopy. Lifetimes of the long-lived transient species in 4c, 4d and 4f are given in Table 2; data for thin-film nsTA experiments are given in SI Fig. S8.

**Table 2:** Lifetime of the longest-lived component for thin films of 4c, 4d and 4f. Data in SI Fig.S8. Errors determined using the *F*-test with a confidence value of 95%

	*τ* [µs]
4c	1.1^+0.1^_−0.1_
4d	14^+2^_−1_
4f	0.21^+0.05^_−0.04_

Kinetic modelling of the fsTA data on thin films of 4a, 4c, 4d and 4f gives clues as to the origin of the long-lived transient species in the latter three of these compounds. In each case, a short-lived component decays quickly (*τ* ≈ 1–10 ps) after excitation, giving rise, in each case, to a much longer-lived second species (shown in SI Fig. S6, in blue). In 4a, the lifetime of this second species is ∼2 ns, after which it decays to the ground state. In 4c, 4d and 4f the lifetimes of the second species are shorter (0.4–1.7 ns) and they each evolve into third species which persists with lifetimes beyond the duration of the fsTA experiment, but resolved by nsTA (shown in SI Fig. S6 in red, see lifetimes in Table 2). We assign the second species in all four systems to the lowest singlet excited state, S_1_. The identity of the long-lived species identified by nsTA in 4c and 4d is likely a triplet due to its long lifetime.

**Fig. 6:**
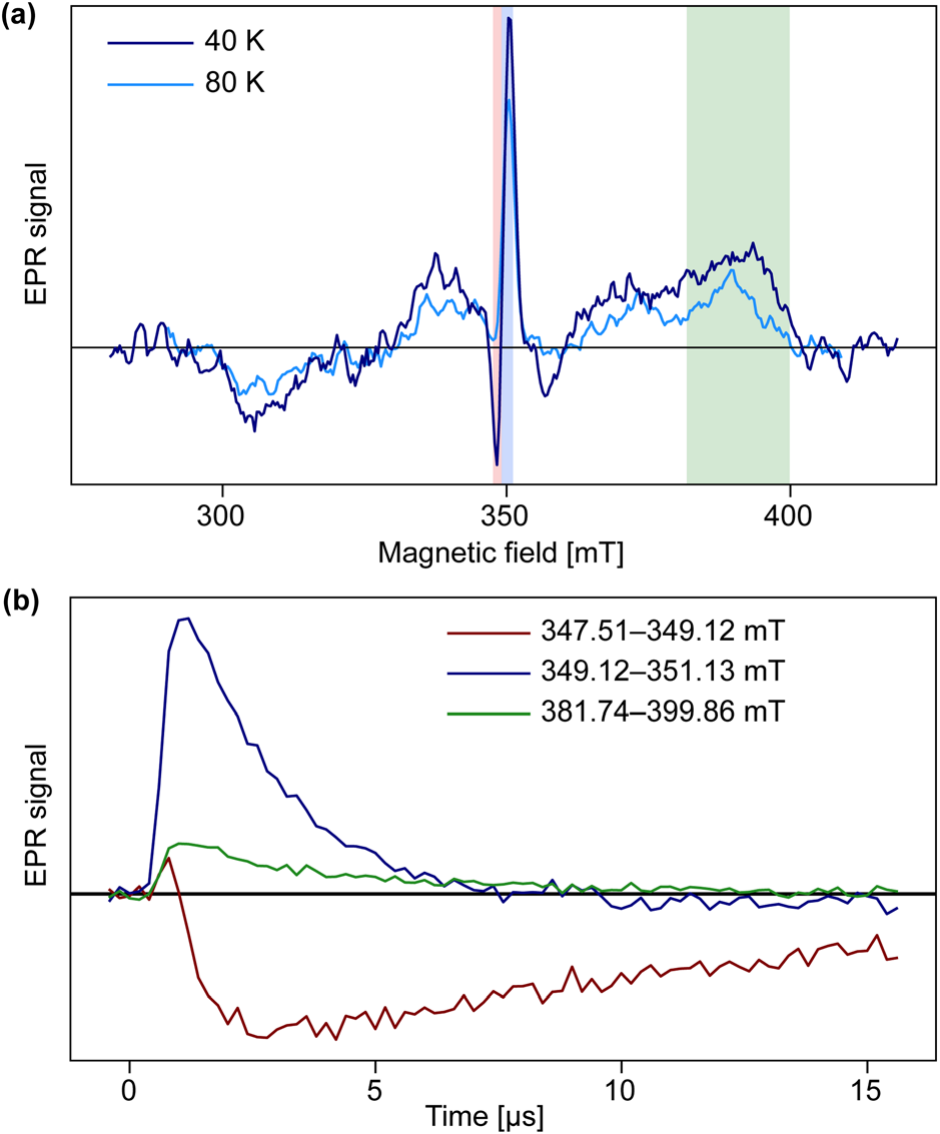
Continuous-wave TR-EPR spectrum of 4f as a pulverized thin film. (a) 40 K and 80 K, 0.6–1.0 µs after excitation; line smoothed using a 5-point moving average; (b) 80 K, time evolution summed across magnetic field ranges shown in top panel; data averaged in 200 ns intervals.

To further investigate the nature of this long-lived state, and develop our understanding of the earlier MPL results, we conducted time-resolved electron paramagnetic resonance (TR-EPR) spectroscopy on a thin film of 4f prepared by thermal evaporation, shown in Fig. 6. Full 2D TR-EPR surface plots are provided in SI Fig. S9 and S10; EPR spectra at various time points are given in SI Fig. S11.

Upon excitation of 4f at 510 nm, a broad triplet signal emerges (Fig. 6, green) and persists for 1–2 µs after excitation. Concurrent with this signal is a sharp absorptive feature at 350 mT. Later, a sharp emissive feature forms at 348 mT (Fig. 6, red), which persists beyond 15 µs after excitation. Viewed in isolation, the *eeaeaa* spin polarization pattern of the initial triplet signal indicates that it was generated by ISC.^40^ This interpretation of the data supports the hypothesis, based on the fsTA, nsTA and MPL results, that a triplet is generated by ISC and subsequently annihilated by TTA or a related doublet–triplet process. If the triplets are annihilated back to S_1_, we should observe delayed fluorescence from that state. To test this hypothesis, we measured the time-resolved photoluminescence (TRPL) of thin films of 4f (SI Fig. S12), which revealed that the emission is dominated by prompt fluorescence (*τ* < 1 ns), with a long (>100 ns) tail of delayed fluorescence, which offers evidence for TTA-UC.

In a TR-EPR study of anthradithiophene films, which undergo the same ISC → TTA process, a sharp emissive feature (∼348 mT) was observed with increasing laser fluence and temperature.^41^ The authors speculated that this feature could indicate a spin-correlated radical pair resulting from charge-separation of the ISC-born triplet, occurring concurrently with TTA.^41^ We suggest that the sharp emissive feature shown in 4f (Fig. 6a) might originate from a similar process. Viewing our data along the time axis (Fig. 6b) the emissive signal (348 mT, shown in red) builds up after the triplet signal (shown in green) reaches its maximum intensity, and reaches its peak 2.5 µs after excitation. Crucially, as has been reported for anthradithiophene films,^41^ despite the calculated thermodynamic favorability of 4f to SF, we did not observe any^5^(TT) quintet species.^42,43^

### Electronic descriptors of aromaticity

Once we had in hand spectroscopic data for compounds 4a, 4c, 4d and 4f, we performed a small computational benchmark to select a suitable functional and basis set for further calculations. From this benchmarking study, which is described in SI Section S5.2, B3LYP/6-311G(d,p)^44–46^ was selected as the appropriate method for calculating various properties of these compounds. Full results are given in SI Section S5.3. We used TD-DFT to calculate the vertical excitation energies of the singlet (*E*(S_1_)_v_). To explore the role of vibrational relaxation in the S_1_ state,^47^ we also attempted to calculate the adiabatic *E*(S_1_)_a_ for 4a, 4c, 4d, and 4f, but results from TD-DFT were inconsistent with the experimental results, placing *E*(S_1_)_a_ beneath *E*(T_1_) (see SI Section S5.3). Evaluation of excited-state aromaticity in S_1_ is challenging due to the failure of single-determinant methods (such as TD-DFT) to accurately describe the S_1_ wavefunction.^48^ Consistent with our preliminary calculations using ωB97XD, these calculations showed an improved *E*(S_1_)/*E*(T_1_) ratio in 4d and 4f compared to 4a (Table 3). The linear correction method of Tayebjee and co-workers, given here as *E*(T_1_)_c_, which gives accurate triplet energies for some π-conjugated heterocycles, gave an *E*(S_1_)/*E*(T_1_) ratio of 2.32 for 4f.^49^ This method was not applied to 4c and 4d due to their (*n*–π*) excitation character, for which the linear correction method is reported to be inaccurate.^49^ The fact that we saw no evidence of SF in 4f despite these results, but instead the reverse process (TTA-UC), highlights the challenges associated with predicting SF capability using DFT-derived excited state energies alone. Nonetheless, DFT provides a valuable window through which to explore the structural and electronic features that affect the singlet–triplet gap. Therefore, we analysed the electronic and magnetic descriptors of aromaticity present in these systems.

**Table 3:** Calculated singlet (TD-B3LYP/6-311G(d,p)) and triplet excitations of 4a, 4c, 4d, and 4f. All energies are given in units of eV.^a^ Structures with singlet and triplet energies sufficient for *exo*- or isothermic singlet fission are marked in green, others are marked in red

	*E*(S_1_)_v_	*E*(T_1_)_a_	*E*(T_1_)_c_	*E*(S_1_)/*E*(T_1_)_a_	*E*(S_1_)/*E*(T_1_)_c_
4a	3.03	1.97	1.49	1.54	2.03
4c	2.40	1.64	n.d.	1.46	n.d.
4d	2.20	1.37	n.d.	1.61	n.d.
4f	2.66	1.33	1.15	2.00	2.32

a *E*(T_*n*_)_a_ and *E*(T_1_)_c_ are the adiabatic (B3LYP/6-311G(d,p) and corrected (B3LYP/3-21G) triplet energies of *n*th state respectively.

**Fig. 7:**
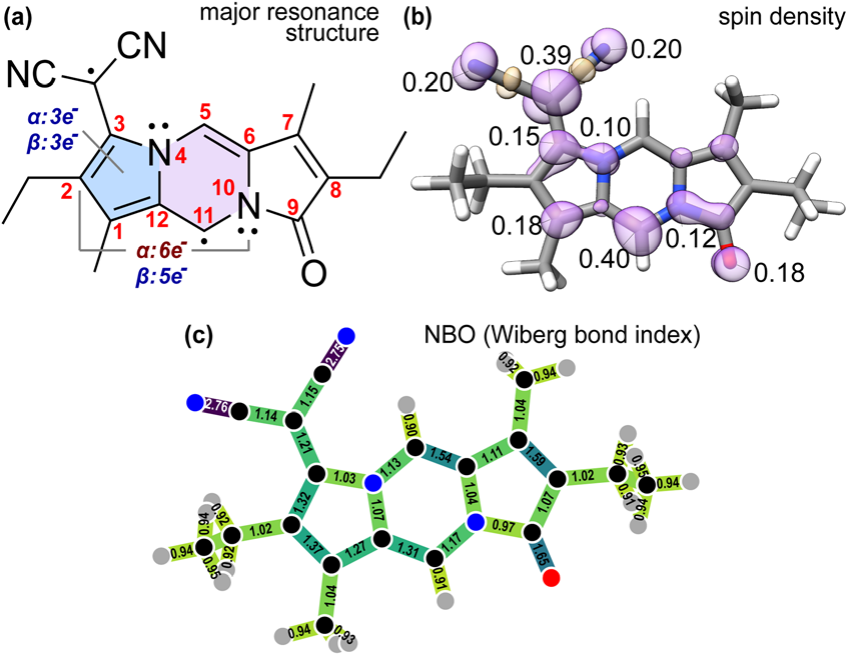
(a) Major resonance structure assignment of 4f in the T_1_ state, with Mandado (anti)aromatic rings and ring atom numbers marked. (b) Calculated spin density isosurface of triplet 4f, isovalue = 0.005. (c) Wiberg bond indices calculated by NBO for triplet 4f.

Although 4a has *C*_*i*_ symmetry in S_0_, it desymmetrizes to *C*_1_ in the optimized T_1_ state. This symmetry-breaking effect can be understood by employing the spin-separated concept of aromaticity introduced by Mandado.^50^ Here, the number of π-electrons in each spin manifold (α/β, or up/down) is counted; a spin-manifold with (2*n* + 1) π-electrons contributes aromaticity, (2*n*) contributes anti-aromaticity.^50^ Mandado's rules unify the Hückel and Baird rules for ground- and excited-state aromaticity.^51^ A 4*n* π-electron Baird aromatic cycle with two unpaired electrons contains (2*n* + 1) π-electrons in the α manifold ([2*n* − 1] paired and 2 unpaired), and (2*n* − 1) π-electrons in the β manifold, so both spin systems are aromatic.^52^ To assign the aromaticity of our structures using Mandado's rules, we analyzed the bonding within these structures using natural bond orbital (NBO) methods^53^ and the global electron density of delocalized bonds (EDDB_G_).^54–56^ We used the bond index developed by Wiberg,^57^ calculated using the NBO 7.0 software, to quantify spin-separated bond order in a chemically intuitive fashion. Visualizations of Wiberg bond index given by NBO for 4a, 4c, 4d and 4f are given in SI Fig. S19–S22. Spin density isosurfaces for these compounds are given in SI Fig. S27–S30; EDDB_G_ isosurfaces are given in SI Fig. S31–S34. NBO and spin density results for compound 4f in the triplet state, and its major resonance structure are shown in Fig. 7.

In 4a, the calculated spin density isosurface (SI Fig. S27) shows unpaired electron density at the oxygen atom, and at the central ring, creating a 6π-electron aromatic pyrrole unit between these points. We ascribe the dominant resonance structures of 4a, 4c and 4f in the triplet state to this asymmetric configuration, with 4d adopting a symmetrical resonance-hybrid structure (SI Fig. S36). In 4f (Fig. 7b), the aromatic pyrrole unit is adjacent to the dicyanomethylene group. Dicyanomethylene is a common feature in organic diradical(oid)s as the electron-withdrawing cyano groups stabilize spin at the central carbon atom.^58^ In 4f, we posit that this stabilization increases the weight of the resonance structure containing the aromatic pyrrole unit, which lowers the energy of the T_1_ state in 4f compared to that in the other systems, leading to a more favorable calculated *E*(S_1_)/*E*(T_1_). This effect also increases the energy of the T_1_ → T_2_ transition from 0.69 (4a) to 1.04 eV (4f) as calculated by TD-DFT, which would deactivate the parasitic T_1_ + T_1_ → T_2_ + S_0_ annihilation pathway.^4^ The geometric dimension of aromaticity is typically evaluated using the harmonic oscillator model of aromaticity (HOMA),^59,60^ but HOMA cannot be used to assign aromaticity in individual rings of a fused-ring system because a shared bond cannot be isolated to a single ring. However, in this case we can obtain some insight by evaluating the bond length alternation associated with the four unshared bonds of the aromatic pyrrole ring in the triplet state of 4f. The calculated bond lengths for this ring range from 1.406–1.420 Å in the triplet state, compared to 1.374–1.462 Å in the singlet state, reflecting relatively equalized bond lengths with partial double bond character and indicating aromaticity gain. Calculated bond lengths of 4a, 4c, 4d and 4f in the singlet and triplet states are given in SI Fig. S23–S26.The EDDB_G_ isosurface of the 4f triplet state (SI Fig. S34) also shows a continuous delocalization pathway through the two rings closest to the dicyanomethylene group. In the major resonance structure given in Fig. 7a, according to Mandado's (2*n* + 1) rule this ring system is antiaromatic in the α manifold and aromatic in the β manifold, which should confer properties intermediate of aromaticity and antiaromaticity.

### Magnetic descriptors of aromaticity

**Fig. 8:**
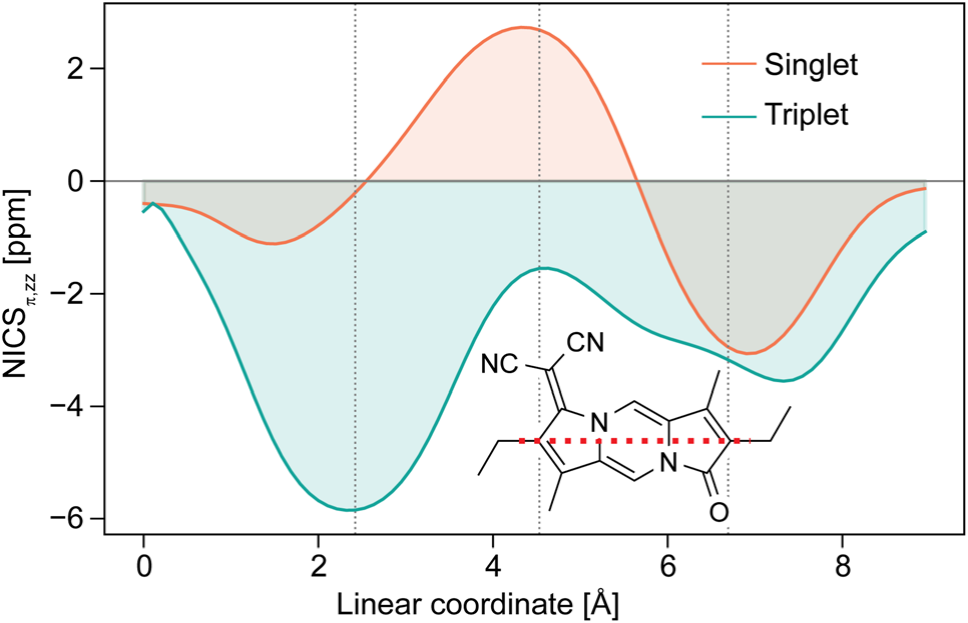
S_0_ and T_1_ NICS_π,*zz*_ (B3LYP/6-311G(d,p)) plot of 4f, measured 1.7 Å above the ring plane. Ring centroids are shown as dotted lines, with the NICS-XY path shown on the molecule in red. Smooth line obtained by interpolation of discrete data points spaced ∼0.1 Å apart.

Nucleus-independent chemical shift (NICS)^61,62^ calculations were performed on compounds 4a, 4c, 4d and 4f, and the π-contributions to the NICS were isolated using the σ-only model as developed by Stanger and co-workers.^63^ NICS_π,*zz*_ data for 4f are shown in Fig. 8, with 4a, 4c and 4d given in SI Fig. S35.

Despite the 8π-electron, Hückel antiaromatic 1,4-diacyl-1,4-dihydropyrazine (DADHP) ring contained in the singlet states of these compounds,^64^ the paramagnetic shielding effect is weak (∼2–5 ppm), suggesting that the central ring is essentially non-aromatic in its ground state. Within this DADHP ring, the C=C bond length measured by XRD is 1.348 Å, typical of a fully localized double bond. However, the length of the adjacent C–N bond between atoms 4 and 5 is 1.385 Å, which indicates partial double bond character, contrasting with the fully bond length alternant regime expected of distorted antiaromatic systems. In addition to the lack of strong paramagnetic shielding and localized bond lengths, the other manifestations of antiaromaticity—instability to air and light, and symmetry-forbidden and low-energy HOMO → LUMO transitions—are completely absent. Thus, we consider the central ring to be partially olefinic in character, potentially due to the resonance character of amides. The excited-state aromaticity gain in the triplet state of 4f, discussed in the previous section, is reflected in the NICS_π,*zz*_ data as a strong diatropic ring current corresponding to enhanced aromaticity in the pyrrole ring adjacent to the dicyanomethylene group, consistent with the NBO analysis (Fig. 7a, blue). The weakened magnetic shielding at the central ring reflects a counterbalancing effect of the mixed Mandado (anti)aromaticity of the system comprising the two rings closest to the dicyanomethylene group, as discussed earlier. Thus, our aromaticity assignments using Mandado's (2*n* + 1) rule agree with the electronic and magnetic descriptors of aromaticity discussed in this section, and our experimental data.

Stanger has recently reported that NICS can be used to screen for SF capability.^65^ While all four compounds pass Stanger's “tropicity difference” (<*c*. 4 ppm) criterion (SI Fig. S35), the ring current structure in the triplet state for 4a, 4c and 4f is markedly different from the singlet. Stanger's criteria hold that a given compound should have a similar ring current structure in its singlet and triplet states to be suitable for SF,^65^ which would exclude these compounds.

## Conclusions

Research into SF chromophores can benefit from the application of aromaticity rules as a design heuristic, and requires robust chemical transformations to improve the singlet–triplet energy ratio (*E*(S_1_)/*E*(T_1_)) by exploring the chemical space of derivatives of known π-systems. We identified diverse excited-state behaviors on a set of chromophores obtained by thionation of a cyclic amide chromophore, followed by silver(i)-mediated Knoevenagel condensation with dicyanomethylene. The driving force behind the improved *E*(S_1_)/*E*(T_1_) was found to be excited-state aromaticity gain, enhanced by the stabilizing effect of dicyanomethylene on unpaired electron density. Fully understanding the (anti)aromatic character of this structure in the triplet state required a spin-separated approach, using Mandado's (2*n* + 1) aromaticity rule. We did not observe SF in our lead chromophore, possibly reflecting the importance of other factors such as molecular packing or exciton dynamics in the molecular dimer aggregate.^66^ The insights gained from this simple set of transformations should aid the rational design of stable organic chromophores featuring the cyclic amide moiety that are capable of exothermic SF.

## Author contributions

All the authors contributed to data collection and analysis. DJG and MDP drafted the manuscript and all authors reviewed and edited it.

## Conflicts of interest

The authors have no conflicts to declare.

## Supplementary Material

SC-OLF-D6SC05784E-s001

SC-OLF-D6SC05784E-s002

## Data Availability

CCDC 2480691 and 2480716 contain the supplementary crystallographic data for this paper.^67*a*,*b*^ Cartesian coordinates associated with computational chemistry calculations are available at 10.6084/m9.figshare.32953976. The data supporting this article have been included as part of the supplementary information (SI). Supplementary information is available. See DOI: https://doi.org/10.1039/d6sc05784e.
