# Supplementary material for: Carbonyl derivatization tunes triplet-state aromaticity and dynamics in fused-ring cyclic amides
Source: Chem Sci. 2026 Aug 10;17(39):18782–90. doi: 10.1039/d6sc05784e (PMC13487667; doi:10.1039/d6sc05784e)
Supplement: Supplementary file 1 [file SC-017-D6SC05784E-s001.pdf]

Supplementary Information (SI) for Chemical Science.
This journal is © The Royal Society of Chemistry 2026

Chemical Science

Electronic Supplementary Information

Carbonyl Derivatization Tunes Triplet-State Aromaticity and Dynamics in Fused-Ring Cyclic Amides

Dexter J. Gordon, Damon M. de Clercq, Ben P. Carwithen, Adrian Mena, Max R. Bonengel, Alexander J. Baldacchino, Mohan Bhadbhade, Ruoming Tian, Dane R. McCamey, Timothy W. Schmidt, Martin D. Peeks*

* Correspondence: m.peeks@unsw.edu.au

Table of Contents

S1. General procedures 3

S2. Synthesis 4

S2.1 **2** 3-ethyl-5-hydroxymethylene-4-methyl-3-pyrrolin-2-one 4

S2.2 **3** 3-ethyl-1-((4-ethyl-3-methyl-5-oxo-1,5-dihydro-2*H*-pyrrol-2-ylidene)methyl)-4-methyl-1,5-dihydro-2*H*-pyrrol-2-one 4

S2.3 **4a** 2,7-diethyl-1,6-dimethyl-3*H*,8*H*-dipyrrolo[1,2-*a*:1',2'-*d*]pyrazine-3,8-dione ... 5

S2.4 **4b** 2,7-diethyl-1,5,6-trimethyl-3*H*,8*H*-dipyrrolo[1,2-*a*:1',2'-*d*]pyrazine-3,8-dione 6

S2.5 **4c** 2,7-diethyl-1,6-dimethyl-8-thioxo-3*H*,8*H*-dipyrrolo[1,2-*a*:1',2'-*d*]pyrazin-3-one 7

S2.6 **4d** 2,7-diethyl-1,6-dimethyl-3*H*,8*H*-dipyrrolo[1,2-*a*:1',2'-*d*]pyrazine-3,8-dithione 7

S2.7 **4e** 2,7-diethyl-1,5,6-trimethyl-8-thioxo-3*H*,8*H*-dipyrrolo[1,2-*a*:1',2'-*d*]pyrazin-3-one 8

S2.8 **4f** (2,7-diethyl-1,6-dimethyl-8-oxo-3*H*,8*H*-dipyrrolo[1,2-*a*:1',2'-*d*]pyrazin-3-ylidene)malononitrile 9

S2.9 **4g** (2,7-diethyl-1,6-dimethyl-8-thioxo-3*H*,8*H*-dipyrrolo[1,2-*a*:1',2'-*d*]pyrazin-3-ylidene)malononitrile 10

S2.10 **5** 2-amino-4-ethyl-5-methylpyrimido[2,1,6-*cd*]pyrrolizine-3,7-dicarbonitrile 10

S2.11 Synthetic optimization of **4a** 11

S2.12 Synthetic optimization of **4c**, **4d** 12

S3. Spectroscopy 13

S3.1 UV-visible absorption 13

S3.2 Spectrofluorometry 14

S3.3 Magnetic photoluminescence 15

S3.4 Femtosecond transient absorption spectroscopy 16

S3.5 Nanosecond transient absorption spectroscopy 22

S3.6 Transient electron paramagnetic resonance spectroscopy 27

S3.7 Time-resolved photoluminescence spectroscopy 29

S4. Crystallography 31

S4.1 Experimental details 31

S4.2 Crystal structures 33

S5. Computational 35

S5.1 General procedures 35

S5.2 Benchmarking of computational methods 35

S5.3 $S_0 \rightarrow S_1$ and T_1 transition energy results 37

S5.4 Natural bond orbital (NBO) analysis 40
S5.5 Calculated bond lengths 42
S5.6 Spin density calculations 43
S5.7 Electron density of delocalized bonds (EDDB) calculations 45
S5.8 Nucleus-independent chemical shift 46
S6. Additional mechanisms 49
S7. NMR spectra 50
S8. References 60

S1. General procedures

Chemicals were purchased from Sigma-Aldrich, ChemSupply Australia and Combi-Blocks Inc., and used as received unless otherwise noted. Argon 4.7 was obtained from Coregas. Solvents used in reactions were dried over molecular sieves and degassed by bubbling with inert gas for 15-30 minutes. NMR spectra were measured using a Bruker Avance III HD 400 MHz or Bruker Avance III HD 600 MHz NMR spectrometer. The residual solvent peaks were used as the internal reference. Chemical shifts (δ) are reported in ppm, with s = singlet, d = doublet, t = triplet, q = quartet, br s = broad singlet, m = multiplet. High resolution mass spectrometry (HRMS) was measured on a Thermo LTQ Orbitrap XL using nanospray ionization (NSI).

S2. Synthesis

S2.1 **2** 3-ethyl-5-hydroxymethylene-4-methyl-3-pyrrolin-2-one

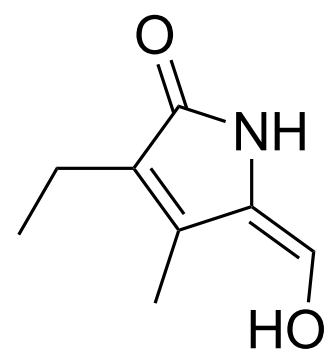

Enol **2** was prepared according to a procedure adapted from that reported by Plieninger and co-workers.[1] Pyrrolinone **1** (9.983 g, 79.75 mmol) was added to a dry flask, which was purged with inert gas. Anhydrous, degassed ethanol (20 mL) and ethyl formate (15 mL, 250 mmol) were added to this flask *via* syringe and swirled until everything dissolved. To a separate dry, inert gas-filled flask, ethanol (50 mL) and 21% sodium ethoxide solution in ethanol (44 mL, 120 mmol) were added *via* syringe. To this flask, the solution of pyrrolinone **1** was added *via* syringe, and the mixture was heated to 45 °C. Over the next hour, the reaction mixture became cloudy. After 45 minutes, the reaction was removed from heating and quenched with water (2 mL), causing some coagulation of the precipitate.

The suspension was filtered through a glass frit and washed twice with ethanol. Then, water (*c.* 10 mL) was added into the frit to dissolve the solids and pass the solvated product through into a separate flask. Meanwhile, the filtrate from the ethanol wash was evaporated under reduced pressure until a yellowish solid precipitated out of solution. This was again filtered through the frit, then the solid product was dissolved with water (*c.* 10 mL) to pass through into a flask. To the dissolved product, 1 M acetic acid (100 mL, 10 mmol) was added slowly, causing a white solid to precipitate. This was filtered through a glass frit and washed with water (*c.* 100 mL), then the paste-like solid was washed with ethanol (*c.* 10 mL) and dried under vacuum to yield a beige powder (6.718 g, 43.86 mmol, 55%). ^{1}H NMR (DMSO-d_6, 400 MHz, 298 K): δ 9.71 (br s, 1H, O-H), 9.30 (s, 1H, N-H), 6.54 (s, 1H, =C-H), 2.16 (q, J = 8 Hz, 2H, Et. CH_2), 1.93 (s, 3H, Me. CH_3), 0.96 (t, J = 8 Hz, 3H, Et. CH_3). ^{13}C NMR (DMSO-d_6, 101 MHz, 298 K): δ 169.9, 139.0, 129.1, 128.4, 121.6, 16.5, 14.1, 9.4.

S2.2 **3** 3-ethyl-1-((4-ethyl-3-methyl-5-oxo-1,5-dihydro-2*H*-pyrrol-2-ylidene)methyl)-4-methyl-1,5-dihydro-2*H*-pyrrol-2-one

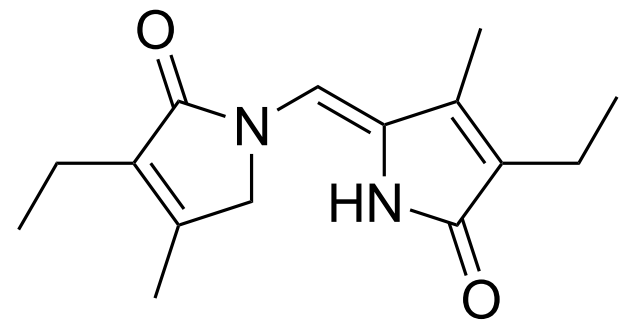

Open dimer **3** was prepared according to a procedure adapted from that reported by Plieninger and co-workers.[2] Pyrrolinone **1** (0.839 g, 6.70 mmol) and enol **2** (1.026 g, 6.70

mmol) were added to a flask, which was purged with inert gas. 2 M aqueous hydrochloric acid (25 mL, 50 mmol) was added, and the mixture was heated to 100 °C. After 3 hours, a yellow precipitate formed, and the reaction mixture was cooled in an ice bath. The precipitate was collected by vacuum filtration and washed with water (*c.* 50 mL), then recrystallized from acetone at −20 °C to yield white, fluffy crystals (0.545 g, 2.09 mmol, 31%). ^{1}H NMR ($CDCl_3$, 400 MHz, 298 K): δ 9.30 (s, 1H, N-H), 6.48 (s, 1H, N-CH), 4.32 (s, 2H, N-CH_2), 2.35 (q, J = 8 Hz, 2H, Et. CH_2), 2.33 (q, J = 8 Hz, 2H, Et. CH_2), 2.08 (s, 3H, Me. CH_3), 2.07 (s, 3H, Me. CH_3), 1.10 (t, J = 8 Hz, 3H, Et. CH_3), 1.09 (t, J = 8 Hz, 3H, Et. CH_3).^{13}C NMR ($CDCl_3$, 101 MHz, 298 K): δ 173.0, 171.2, 149.2, 141.9, 133.9, 131.2, 123.2, 106.4, 55.1, 17.3, 17.2, 13.9, 13.3, 13.2, 10.0.

S2.3 **4a** 2,7-diethyl-1,6-dimethyl-3*H*,8*H*-dipyrrolo[1,2-*a*:1',2'-*d*]pyrazine-3,8-dione

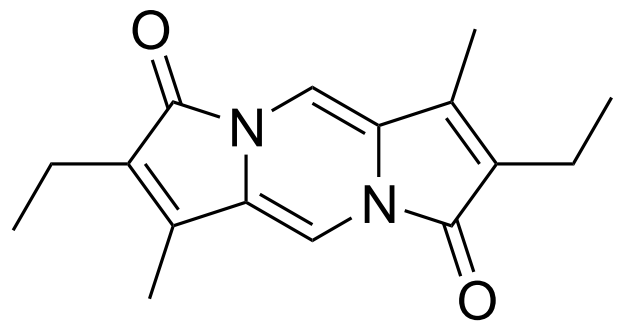

The synthesis of isopyrocoll **4a** was developed based on observations reported by Plieninger and co-workers.[1,2]

Organic Method

Enol **2** (0.099 g, 0.646 mmol) was added to a flask, which was fitted with a reflux condenser and drying tube containing Drierite™. A commercial solution of 4 M hydrogen chloride in dioxane (2.0 mL) was added, and the mixture was heated to 100 °C. After 21 hours, the mixture was removed from heating and transferred to a separatory funnel. Water (3 mL) and dichloromethane (3 mL) were added, and the aqueous phase was extracted 3 times with dichloromethane (*c.* 50 mL), then with ethyl acetate (*c.* 50 mL). The combined organic phase was then dried over anhydrous magnesium sulfate and evaporated under reduced pressure to yield a dark red oil.

The product was purified using silica gel column chromatography (5% methanol in dichloromethane). The yellow fraction was evaporated under reduced pressure to yield an orange solid (0.012 g, 0.046 mmol, 14%). ^{1}H NMR ($CDCl_3$, 400 MHz, 298 K): δ 7.22 (s, 1H, Ar-CH), 2.45 (q, J = 8 Hz, 2H, Et. CH_2), 2.16 (s, 3H, Me. CH_3), 1.14 (t, J = 8 Hz, 3H, Et. CH_3). ^{13}C NMR ($CDCl_3$, 101 MHz, 298 K): δ 163.7, 137.1, 134.0, 123.7, 106.5, 17.3, 13.3, 9.6. UV/vis (CH_2Cl_2): λ_{max} (ε [M^{-1} cm^{-1}]) = 428 (17 000) nm. HRMS: (NSI): m/z: $[M + H]^+$, calcd. for $C_{16}H_{19}N_2O_2^+$: 271.14410; found 271.14367.

Aqueous Method

Enol **2** (1.011 g, 6.598 mmol) was added to a flask, which was purged with inert gas. A mixture of 3 M aqueous hydrochloric acid (10 mL, 30 mmol) and ethanol (5 mL) was added, a reflux condenser was attached, and the mixture was heated to 110 °C. The reaction mixture gradually turned yellow. After 18 hours, the mixture was removed from heating and transferred to a separatory funnel. Water (25 mL) was added, and the aqueous phase was extracted 3 times with dichloromethane (25 mL). The combined organic phase was then dried over anhydrous magnesium sulfate and evaporated under reduced pressure to yield a flaky pale yellow solid.

The solid was purified using silica gel column chromatography (1% methanol in dichloromethane). The yellow fraction was evaporated under reduced pressure to yield an orange solid (0.100 g, 0.370 mmol, 11%). Characterization data as above.

S2.4 **4b** 2,7-diethyl-1,5,6-trimethyl-3*H*,8*H*-dipyrrolo[1,2-*a*:1',2'-*d*]pyrazine-3,8-dione

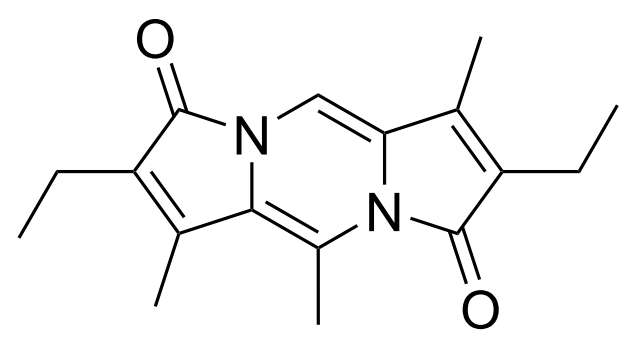

Isopyrocoll **4b** was prepared according to a procedure adapted from that reported by Plieninger and co-workers.[2] Open dimer **3** (0.850 g, 2.95 mmol) was added to a dry flask, which was purged with inert gas. Acetic anhydride (17 mL, 18 g, 180 mmol) was added and the mixture was heated to 140 °C. Concentrated sulfuric acid (4 drops) was added, causing the mixture to turn black. The mixture was refluxed for 40 minutes and then cooled to 0 °C. Water (*c.* 50 mL) was added, and the aqueous phase was extracted 5 times with dichloromethane (*c.* 50 mL). The organic phase was washed with saturated sodium hydrogen carbonate solution (*c.* 50 mL), then water (*c.* 50 mL), then saturated sodium chloride solution (*c.* 50 mL). The organic phase was dried over anhydrous magnesium sulfate, then evaporated under reduced pressure until a small amount of viscous, black liquid remained. This material was passed through a silica gel plug in neat dichloromethane. As the eluent was evaporated, orange needle-like crystals precipitated out, which were washed 3 times with methanol (*c.* 2 mL) and collected (0.1371 g, 0.482 mmol, 16%). ^{1}H NMR ($CDCl_3$, 400 MHz, 298 K): δ 7.24 (s, 1H, pyrazine), 2.90 (s, 3H, 5-CH_3), 2.45 (q, J = 8 Hz, 2H, Et. CH_2), 2.39 (q, J = 8 Hz, 2H, Et. CH_2), 2.35 (s, 3H, Me. CH_3), 2.12 (s, 3H, Me. CH_3), 1.11 (t, J = 8 Hz, 3H, Et. CH_3), 1.11 (t, J = 8 Hz, 3H, Et. CH_3). ^{13}C NMR ($CDCl_3$, 101 MHz, 298 K):

δ 166.5, 162.6, 137.9, 136.3, 133.8, 133.4, 125.6, 124.0, 106.2, 17.0, 16.9, 13.9, 13.6, 13.2, 9.2. HRMS: (NSI): m/z: $[M + Na]^+$, calcd. for $C_{17}H_{20}N_2O_2Na^+$: 307.14170; found 307.14182.

S2.5 **4c** 2,7-diethyl-1,6-dimethyl-8-thioxo-3*H*,8*H*-dipyrrolo[1,2-*a*:1',2'-*d*]pyrazin-3-one

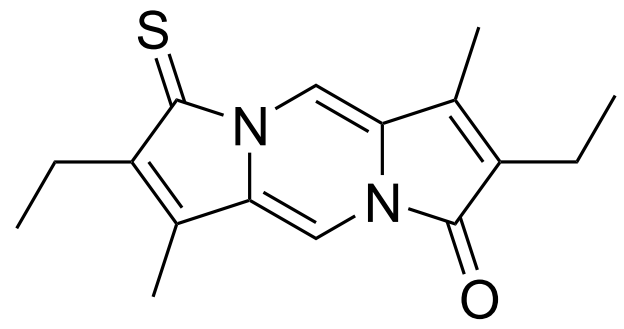

Isopyrocoll **4a** (0.042 g, 0.15 mmol) and Lawesson's reagent (0.187 g, 0.462 mmol) were added to a microwave vial, which was equipped with a rubber septum and purged with inert gas. THF (3 mL) was added, then the septum was replaced with a sealed cap, and the vial was heated in a microwave reactor at 80 °C for 20 minutes. A crimson mixture was obtained, which was transferred to a separatory funnel with ethyl acetate (*c.* 50 mL) and saturated aqueous $NaHCO_3$ (*c.* 50 mL) was added. The organic phase was separated and washed with distilled water (*c.* 50 mL), followed by saturated aqueous NaCl (*c.* 50 mL). The organic phase was then dried over anhydrous magnesium sulfate and evaporated under reduced pressure to yield a red-black solid.

The solid was purified using silica gel column chromatography (1:1 dichloromethane/*n*-hexane + 1% triethylamine). The major product, which appeared as an orange band, was evaporated under reduced pressure and washed 3 times with methanol (*c.* 2 mL) to yield a brick-red solid (0.024 g, 0.08 mmol, 54%). ^1H NMR ($CDCl_3$ + 1% pyridine-d_5, 400 MHz, 298 K): δ 7.91 (s, 1H, Pyr-CH), δ 7.40 (s, 1H, Pyr-CH), 2.70 (q, J = 8 Hz, 2H, Et. CH_2), 2.45 (q, J = 8 Hz, 2H, Et. CH_2), 2.23 (s, 3H, Me. CH_3), 2.21 (s, 3H, Me. CH_3), 1.16 (t, J = 8 Hz, 6H, Et. CH_3), 1.16 (t, J = 8 Hz, 6H, Et. CH_3). ^{13}C NMR ($CDCl_3$ + 1% pyridine-d_5, 101 MHz, 298 K): δ 178.7, 163.9, 144.4, 139.9, 133.9, 133.5, 128.9, 125.5, 109.0, 106.9, 18.9, 17.1, 13.4, 13.1, 9.8, 9.4. UV/vis (CH_2Cl_2): λ_{max} (ε [M^{-1} cm^{-1}]) = 493 (24 000), 527 (25 000) nm. HRMS: (NSI): m/z: $[M + H]^+$, calcd. for $C_{16}H_{19}N_2OS^+$: 287.12126; found 287.12100.

S2.6 **4d** 2,7-diethyl-1,6-dimethyl-3*H*,8*H*-dipyrrolo[1,2-*a*:1',2'-*d*]pyrazine-3,8-dithione

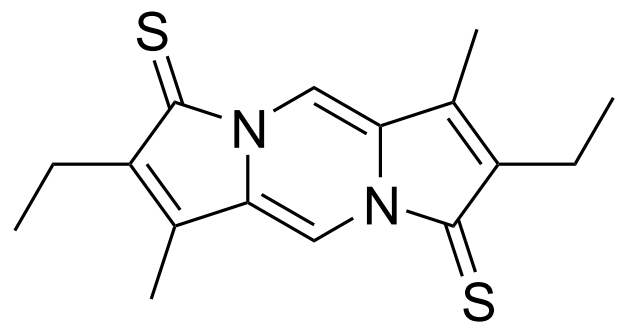

Isopyrocoll **4a** (0.040 g, 0.15 mmol) and Lawesson's reagent (0.301 g, 0.744 mmol) were added to a microwave vial, which was equipped with a rubber septum and purged with inert gas. THF (3 mL) was added, then the septum was replaced with a sealed cap, and the vial was heated in a microwave reactor at 80 °C for 30 minutes. A

purple mixture was obtained, which was transferred to a separatory funnel with ethyl acetate (*c.* 50 mL) and saturated aqueous $NaHCO_3$ (*c.* 50 mL) was added. The organic phase was separated and washed with distilled water (*c.* 50 mL), followed by saturated aqueous NaCl (*c.* 50 mL). The organic phase was then dried over anhydrous magnesium sulfate and evaporated under reduced pressure to yield a purple-black solid.

The solid was purified using silica gel column chromatography (1:1 dichloromethane/*n*-hexane + 1% triethylamine). The major product, which appeared as a purple band, was evaporated under reduced pressure to yield a purple-black solid (0.027 g, 0.09 mmol, 60%). ^{1}H NMR ($CDCl_3$, 400 MHz, 298 K): δ 7.95 (s, 1H, Pyr-CH), 2.68 (q, J = 8 Hz, 2H, Et. CH_2), 2.25 (s, 3H, Me. CH_3), 1.16 (t, J = 8 Hz, 3H, Et. CH_3). ^{13}C NMR ($CDCl_3$, 101 MHz, 298 K): δ 181.2, 144.4, 136.0, 130.4, 108.4, 18.8, 13.4, 9.7. UV/vis (CH_2Cl_2): λ_{max} (ε [M^{-1} cm^{-1}]) = 558 (42 000), 587 (44 000), 600 (60 000) nm. HRMS: (NSI): m/z: [M + H]$^+$, calcd. for $C_{16}H_{19}N_2S_2^+$: 303.09842; found 303.09806.

S2.7 **4e** 2,7-diethyl-1,5,6-trimethyl-8-thioxo-3*H*,8*H*-dipyrrolo[1,2-*a*:1',2'-*d*]pyrazin-3-one

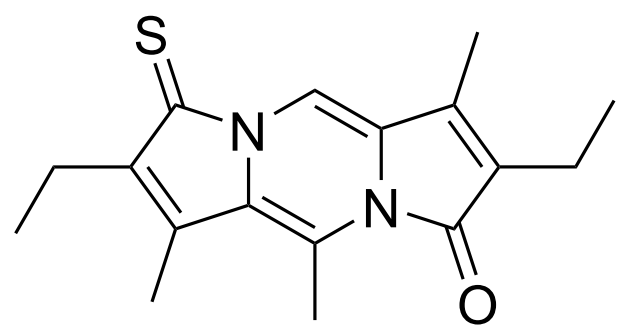

Isopyrocoll **4b** (0.106 g, 0.374 mmol) and Lawesson's reagent (0.768 g, 1.90 mmol) were added to a microwave vial, which was equipped with a rubber septum and purged with inert gas. THF (10 mL) was added, then the septum was replaced with a sealed cap, and the vial was heated in a microwave reactor at 80 °C for 30 minutes. A crimson mixture was obtained, which was transferred to a separatory funnel with ethyl acetate (*c.* 50 mL) and saturated aqueous $NaHCO_3$ (*c.* 50 mL) was added. The organic phase was separated and washed with distilled water (*c.* 50 mL), followed by saturated aqueous NaCl (*c.* 50 mL). The organic phase was then dried over anhydrous magnesium sulfate and evaporated under reduced pressure to yield a red-black solid.

The solid was purified using a short silica gel plug in neat dichloromethane. The major product, which appeared as an orange band, was recovered and evaporated under reduced pressure then washed 3 times with methanol (*c.* 2 mL) to yield a dark red solid (0.065 g, 0.22 mmol, 58%). ^{1}H NMR ($CDCl_3$ + 1% pyridine-d_5, 400 MHz, 298 K): δ 8.01 (s, 1H, pyrazine), 2.93 (s, 3H, 5-CH_3), 2.72 (q, J = 8 Hz, 2H, Et. CH_2), 2.41 (s, 3H, Me. CH_3), 2.39 (q, J = 8 Hz, 2H, Et. CH_2), 2.17 (s, 3H, Me. CH_3), 1.12 (t, J = 8 Hz, 6H, Et. CH_3), 1.12 (t, J = 8 Hz, 6H, Et.

CH_3). ^{13}C NMR ($CDCl_3$ + 1% pyridine-d$_5$, 101 MHz, 298 K): δ 175.4, 166.7, 144.2, 139.4, 134.4, 133.8, 127.4, 126.0, 125.3, 109.1, 18.9, 17.0, 13.8, 13.7, 13.6, 13.1, 9.6. HRMS: (NSI): m/z: $[M + H]^+$, calcd. for $C_{17}H_{21}N_2OS^+$: 301.13691; found 301.13607.

S2.8 **4f** (2,7-diethyl-1,6-dimethyl-8-oxo-3*H*,8*H*-dipyrrolo[1,2-*a*:1',2'-*d*]pyrazin-3-ylidene)malononitrile

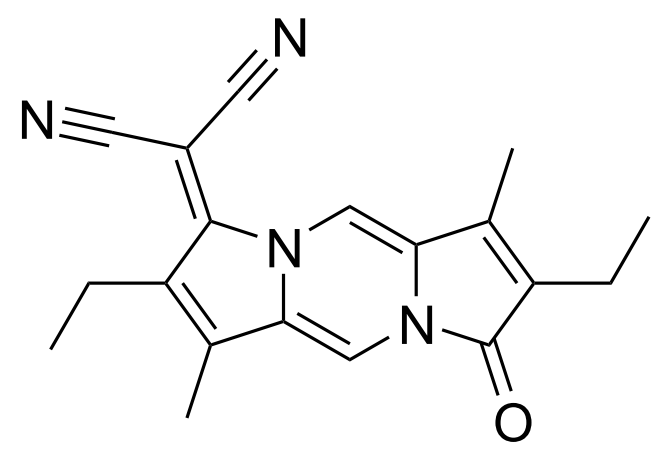

Thioamide **4c** (0.058 g, 0.204 mmol) and malononitrile (0.205 g, 3.11 mmol) were added to a dry reaction flask, which was purged with inert gas. To this flask, triethylamine (1.0 mL, 0.73 g, 7.2 mmol) and toluene (15.0 mL) were added, and the mixture was heated with stirring to 110 °C. In a separate dry flask, silver triflate (0.219 g, 0.85 mmol) was dissolved in toluene (5.0 mL). This solution was transferred to the reaction flask when the latter's contents reached *c.* 105 °C. Upon addition, the mixture became opaque and red-brown, then after 10 minutes a red-pink translucent solution was formed. The mixture was cooled to 0 °C and transferred to a separatory funnel with ethyl acetate (*c.* 50 mL), then saturated aqueous $NaHCO_3$ (*c.* 50 mL) was added. The organic phase was separated and washed with distilled water (*c.* 50 mL), followed by saturated aqueous NaCl (*c.* 50 mL). The organic phase was then dried over anhydrous magnesium sulfate and evaporated under reduced pressure to yield a red solid.

The solid was purified using silica gel column chromatography (1:1 dichloromethane/hexane, 1% triethylamine). The product, which appeared as a pink band, was recovered and evaporated under reduced pressure to yield a red solid (0.021 g, 0.066 mmol, 32%). To remove other trace products, it may be necessary to subject the product to an additional column (1% triethylamine in dichloromethane). ^1H NMR ($CDCl_3$ + 1% pyridine-d$_5$, 400 MHz, 298 K): δ 8.21 (s, 1H, Pyr-CH), 7.54 (s, 1H, Pyr-CH), 2.80 (q, J = 7 Hz, 2H, Et. CH_2), 2.45 (q, J = 8 Hz, 2H, Et. CH_2), 2.19 (s, 6H, Me. CH_3), 1.19 (t, J = 7 Hz, 3H, Et. CH_3), 1.14 (t, J = 8 Hz, 3H, Et. CH_3). ^{13}C NMR ($CDCl_3$ + 1% pyridine-d$_5$, 151 MHz, 298 K): δ 164.1, 150.2, 139.3, 137.7, 135.6, 134.7, 127.8, 125.2, 110.5, 107.5, 43.9, 18.3, 17.2, 14.2, 13.0, 9.7, 9.0. UV/vis (CH_2Cl_2): λ_{max} (ε [M^{-1} cm^{-1}]) = 502 (24 000), 536 (25 000) nm. HRMS: (NSI): m/z: $[M + H]^+$, calcd. for $C_{19}H_{19}N_4O^+$: 319.15534; found 319.15484.

S2.9 **4g** (2,7-diethyl-1,6-dimethyl-8-thioxo-3*H*,8*H*-dipyrrolo[1,2-*a*:1',2'-*d*]pyrazin-3-ylidene)malononitrile

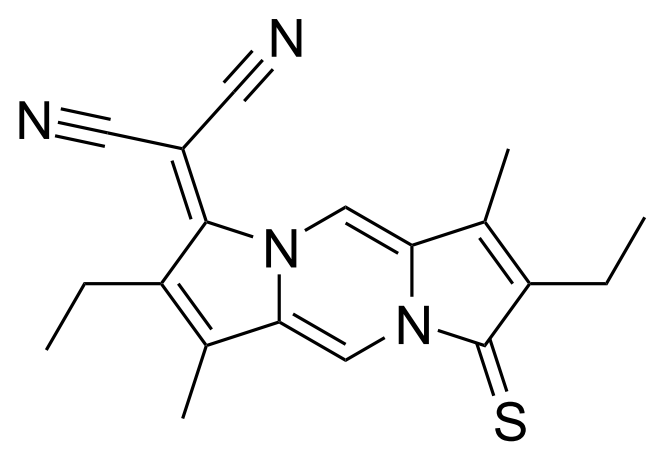

Thioamide **4d** (0.042 g, 0.014 mmol) and malononitrile (0.092 g, 1.39 mmol) were added to a dry reaction flask, which was purged with inert gas. To this flask, triethylamine (0.25 mL, 0.18 g, 1.8 mmol) and toluene (12.5 mL) were added, and the mixture was heated with stirring to 110 °C. In a separate dry flask, silver triflate (0.165 g, 0.64 mmol) was dissolved in toluene (2.0 mL). This solution was transferred to the reaction flask when the latter's contents reached *c.* 90 °C. Upon addition, the mixture became opaque and red-brown, then after 15 minutes a blue translucent solution was formed. The mixture was removed from heating and transferred to a separatory funnel with ethyl acetate (*c.* 50 mL), then saturated aqueous $NaHCO_3$ (*c.* 50 mL) was added. The organic phase was separated and washed with distilled water (*c.* 50 mL), followed by saturated aqueous NaCl (*c.* 50 mL). The organic phase was then dried over anhydrous magnesium sulfate and evaporated under reduced pressure to yield a blue-purple solid.

The solid was purified using silica gel column chromatography (1% triethylamine in dichloromethane). The product, which appeared as a blue band, was recovered and evaporated under reduced pressure to yield a blue-purple solid (0.003 g, 0.01 mmol, 7%). ^{1}H NMR ($CDCl_3$ + 1% pyridine-d_5, 400 MHz, 298 K): δ 8.33 (s, 1H, pyrazine), 8.05 (s, 1H, pyrazine), 2.81 (q, J = 8 Hz, 2H, Et. CH_2), 2.67 (q, J = 8 Hz, 2H, Et. CH_2), 2.22 (s, 3H, Me. CH_3), 2.22 (s, 3H, Me. CH_3), 1.20 (t, J = 8 Hz, 3H, Et. CH_3), 1.13 (t, J = 8 Hz, 3H, Et. CH_3). ^{13}C NMR ($CDCl_3$ + 1% pyridine-d_5, 101 MHz, 298 K): δ 192.9, 150.5, 145.2, 137.8, 137.3, 135.4, 130.1, 129.1, 117.5, 116.4, 115.7, 111.8, 106.9, 18.8, 18.3, 14.2, 13.3, 9.5, 9.2. HRMS: (NSI): m/z: $[M + H]^+$, calcd. for $C_{19}H_{19}N_4S^+$: 335.13304; found 335.13186.

S2.10 **5** 2-amino-4-ethyl-5-methylpyrimido[2,1,6-*cd*]pyrrolizine-3,7-dicarbonitrile

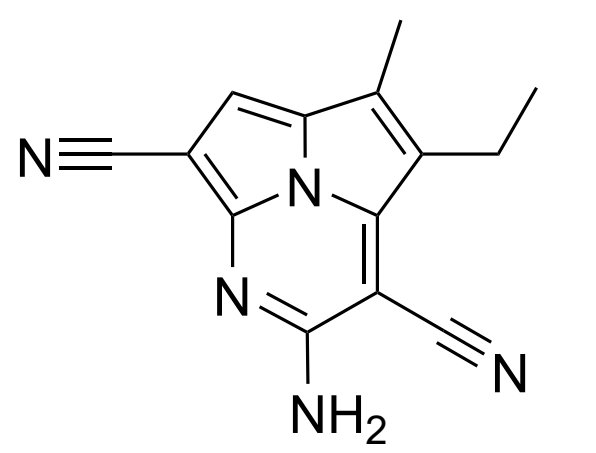

Thioamide **4d** (0.056 g, 0.018 mmol) and malononitrile (0.372 g, 5.64 mmol) were added to a dry reaction flask, which was purged with inert gas. To this flask, triethylamine (1.0 mL, 0.72 g, 7.1 mmol) and toluene (15 mL) were added, and the mixture was heated with stirring to 110 °C. In a separate dry flask, silver

triflate (0.203 g, 0.79 mmol) was dissolved in toluene (2.5 mL). This solution was transferred to the reaction flask when the latter's contents reached *c.* 90 °C. Upon addition, the mixture became opaque and red-brown. Additional silver triflate (0.218 g, 0.85 mmol) in toluene (2.5 mL) was added and heating was continued until TLC indicated consumption of the starting material (about 1 hour). The mixture was removed from heating and transferred to a separatory funnel with ethyl acetate (*c.* 50 mL), then saturated aqueous $NaHCO_3$ (*c.* 50 mL) was added. The organic phase was separated and washed with distilled water (*c.* 50 mL), followed by saturated aqueous NaCl (*c.* 50 mL). The organic phase was then dried over anhydrous magnesium sulfate and evaporated under reduced pressure to yield a brown solid.

The solid was purified using silica gel column chromatography (20–50% ethyl acetate / hexane, 1% triethylamine) to yield a red solid (0.012 g, 0.050 mmol, 27%). ^{1}H NMR ($CDCl_3$, 600 MHz, 298 K): δ 7.66 (s, 1H, pyr.), 5.74 (s, 2H, NH_2), 2.95 (q, J = 8 Hz, 2H, Et. CH_2), 2.53 (s, 3H, Me. CH_3), 1.32 (t, J = 8 Hz, 3H, Et. CH_3). ^{13}C NMR ($CDCl_3$, 151 MHz, 298 K): δ 157.0, 142.0, 141.3, 139.0, 129.1, 125.6, 123.6, 114.8, 114.5, 85.9, 17.6, 15.7, 11.6.

S2.11 Synthetic optimization of **4a**

Table S1 Screening of conditions for synthesis of **4a**. * NMR yield.

#	**Conditions**	**4a [%]**	**3* [%]**	**1* [%]**
1	4 M HCl in dioxane, reflux, 2 h	8	34	*n.d.*
2	4 M HCl in dioxane, reflux, 3 h	12	58	*n.d.*
3	4 M HCl in dioxane, reflux, 18 h	14	*n.d.*	*n.d.*
4	2 M HCl, 2:1 H_2O:EtOH, 0.1 M **2**, reflux, 18 h	2	3	40
5	2 M HCl, 2:1 H_2O:EtOH, 0.4 M **2**, reflux, 18 h	11	45	38
6	2 M $HCl_{(aq)}$, reflux, 18 h	3−11	*c.* 10−30	*c.* 15
7	2 eq. $BF_3{\cdot}OEt_2$, dioxane, reflux, 2 h	14	25	20
8	2 eq. $BF_3{\cdot}OEt_2$, dioxane, 3 Å MS, reflux, 2 h	3	4	>90
9	3 eq. TFAA, dioxane, reflux, 3 h	*n.d.*	*n.d.*	*n.d.*

In most entries in **Table S1**, we isolated isopyrocoll **4a** in modest yield, with crude NMR showing open dimer **3** as the major product; with lower concentrations of acid, pyrrolinone **1** was also identified. To explain these observations, we posit that enol **2** undergoes Lewis acid-

promoted decarbonylation to pyrrolinone **1**, which then reacts with an additional equivalent of enol **2** to yield open dimer **3**, as reported in the literature.[3,4] This side reaction competes with the desired cyclodimerization of **2** to **4a**, explaining the low yields. There are two exceptions to this pattern, which offer some further potential insight into the mechanism.

Firstly, under aqueous acidic conditions (entry 6, **Table S1**)—the original conditions reported by Plieninger and co-workers[2]—the reaction is heterogeneous because enol **2** has poor solubility in water. Consequently, the yields of isopyrocoll **4a** are highly variable, between 3−11%, and open dimer **3** is returned as the major product. However, when sufficient ethanol is added to completely dissolve **2** (entry 4), the decarbonylation occurs rapidly and pyrrolinone **1** is obtained as the major product. We posit that in the aqueous conditions (entry 6), the enol **2** is slowly decarbonylated to give **1**, which reacts with the locally high concentration of starting material to yield the open dimer **3**. Secondly, when the reaction is conducted in an organic solvent with $BF_3{\cdot}OEt_2$ as the Lewis acid (entries 7 and 8), the outcomes differ depending on the presence of molecular sieves. In their presence, pyrrolinone **1** is obtained as the major product, while their absence leads to similar amounts of each of **1**, **3** and **4a**. Both condensation steps in the cyclodimerization of **2** to **4a** give water as a by-product, and boron trifluoride decomposes in the presence of water to yield boric and fluoroboric acids,[5] both Brønsted acids. These factors suggest that the cyclodimerization of **2** to **4a** requires a proton source, but the decarbonylation does not.

S2.12 Synthetic optimization of **4c**, **4d**

Table S2 Optimization of conditions for microwave thionation of isopyrocoll **4a**. General conditions: 50 mM in THF, 80 °C, ≤ 200 W.

Lawesson's reagent eq.	Time [min]	4c [%]	4d [%]
3	20	**54**	24
3	30	17	47
5	20	34	55
5	30	15	**60**

S3. Spectroscopy

S3.1 UV-visible absorption

UV-visible absorbance spectra were collected with a Cary 60 UV-visible spectrophotometer using baseline correction, with a scan rate of 300 nm/min, 0.5 nm data interval and 0.1 s averaging time. Samples were measured in a 10 mm quartz cuvette in dichloromethane from 800 to 250 nm.

Molar absorption coefficients were measured using the above instrument at a fixed wavelength and calculated using the Beer-Lambert law:

$$A = \varepsilon c l$$

Plots of absorption against concentration are given in **Figure S1**.

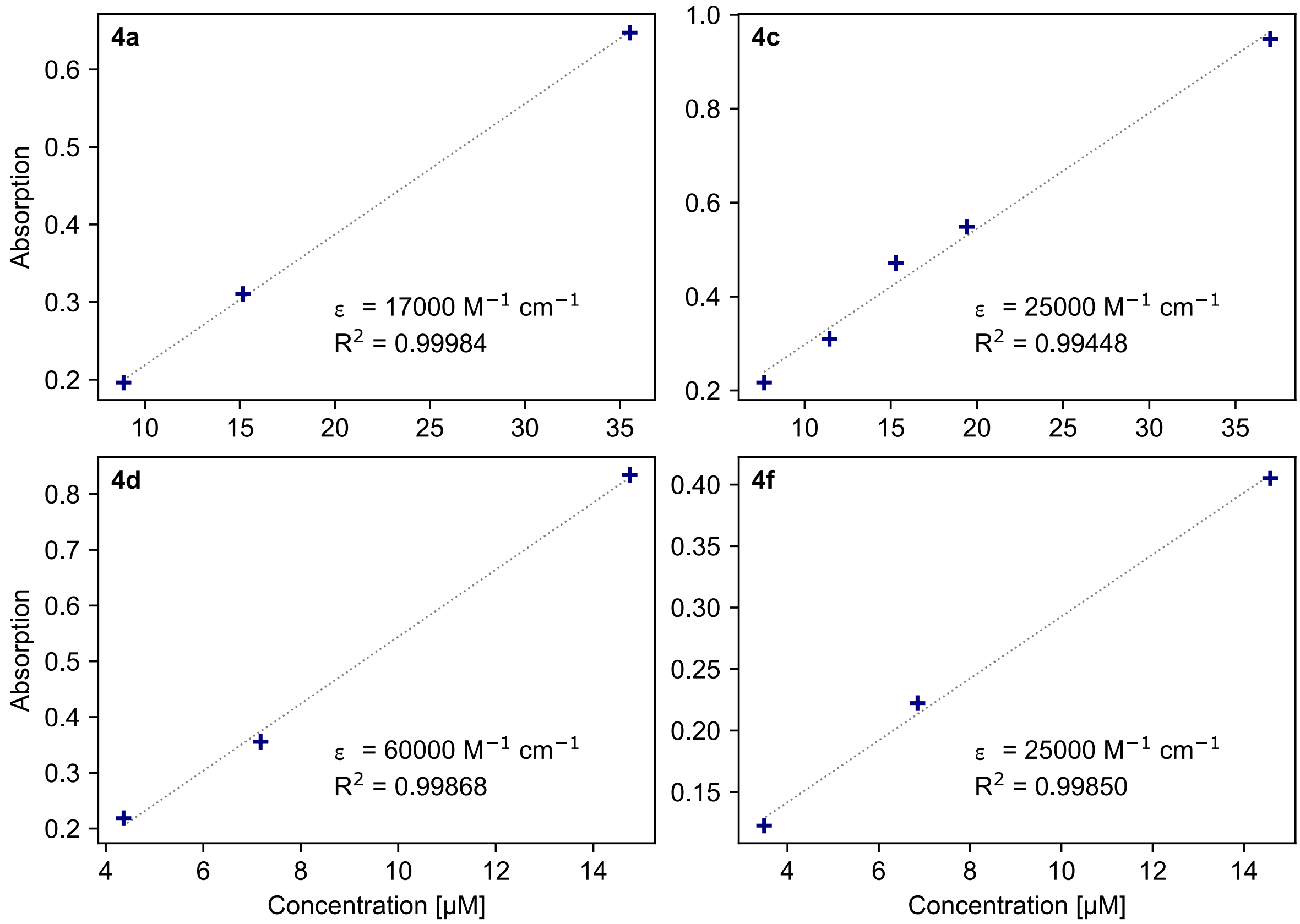

Figure S1 Molar absorption coefficient linear regression plots of **4a**, **4c**, **4d**, and **4f**.

S3.2 Spectrofluorometry

Photoluminescence emission and excitation spectra, time-correlated single photon counting (TCSPC) lifetime measurements and photoluminescence quantum yield (PLQY) measurements were collected using an Edinburgh Instruments FS5 spectrofluorometer. Emission and excitation spectra, and TCSPC measurements, were collected using the SC-05 sample holder. TCSPC measurements were fit by reconvolution with the instrument response function (IRF) to an exponential decay model using the in-built fitting tools in the Fluoracle software, from which lifetime data were obtained. PLQY measurements were collected in triplicate using the SC-30 integrating sphere and reported as observed. It was not necessary to correct for re-absorption due to the low observed quantum yield of the samples. For each technique, samples were measured in a 10 mm quartz 4-window cuvette in non-degassed dichloromethane with a dwell time of at least 0.1 s and a step size of 1 nm.

Table S3 Parameters used for collection of photoluminescence data on solutions of **4a**, **4c**, **4d** and **4f**.

	**4a**	**4c**	**4d**	**4f**
Excitation wavelength [nm]	365	495	N/A	535
Emission wavelength [nm]	545	577	600	580

Table S4 Parameters used for collection of TCSPC data on solutions of **4a** and **4c**.

	**4a**	**4c**
Laser wavelength [nm]	405	405
Timescale [ns]	20	20
Repetition rate [ns]	50	50
τ [ns]	0.24	0.19
χ^2	131	2.38

Table S5 Parameters used for collection of PLQY data on solutions of **4a**, **4c** and **4f**. Errors are shown as the standard deviation of the mean from 3 measurements (4 for **4a**).

	**4a**	**4c**	**4f**
Excitation wavelength [nm]	430	495	502

PLQY_{obs} [%]	1.2 ± 0.2	0.2 ± 0.1	0.8 ± 0.3

S3.3 Magnetic photoluminescence

Magnetic photoluminescence (MPL) was collected on a thermally evaporated thin film sample of **4f** and a *c.* 5 mM solution of the same in dichloromethane. A Magnetech Corp MFG-6-24 24VDC electromagnet was used, driven by a benchtop power supply. The excitation laser was a Thorlabs L520P50 520 nm laser diode, chopped at 93 Hz for lock-in detection. PL was detected with a silicon photodiode with a 550LP filter to block scattered excitation light. The instrument parameters for each measurement are given below.

Table S6 Parameters used for collection of MPL data on **4f**.

	**Solution**	**Thin film**
Excitation wavelength [nm]	520	520
Laser power [mW]	4.3	6.1
Magnetic field sweep [mT]	25 to 210	−210 to 210
Number of scans	300	5

The solution-phase MPL results are given in **Figure S2**; thin-film results are given in **Figure 4** in the main text.

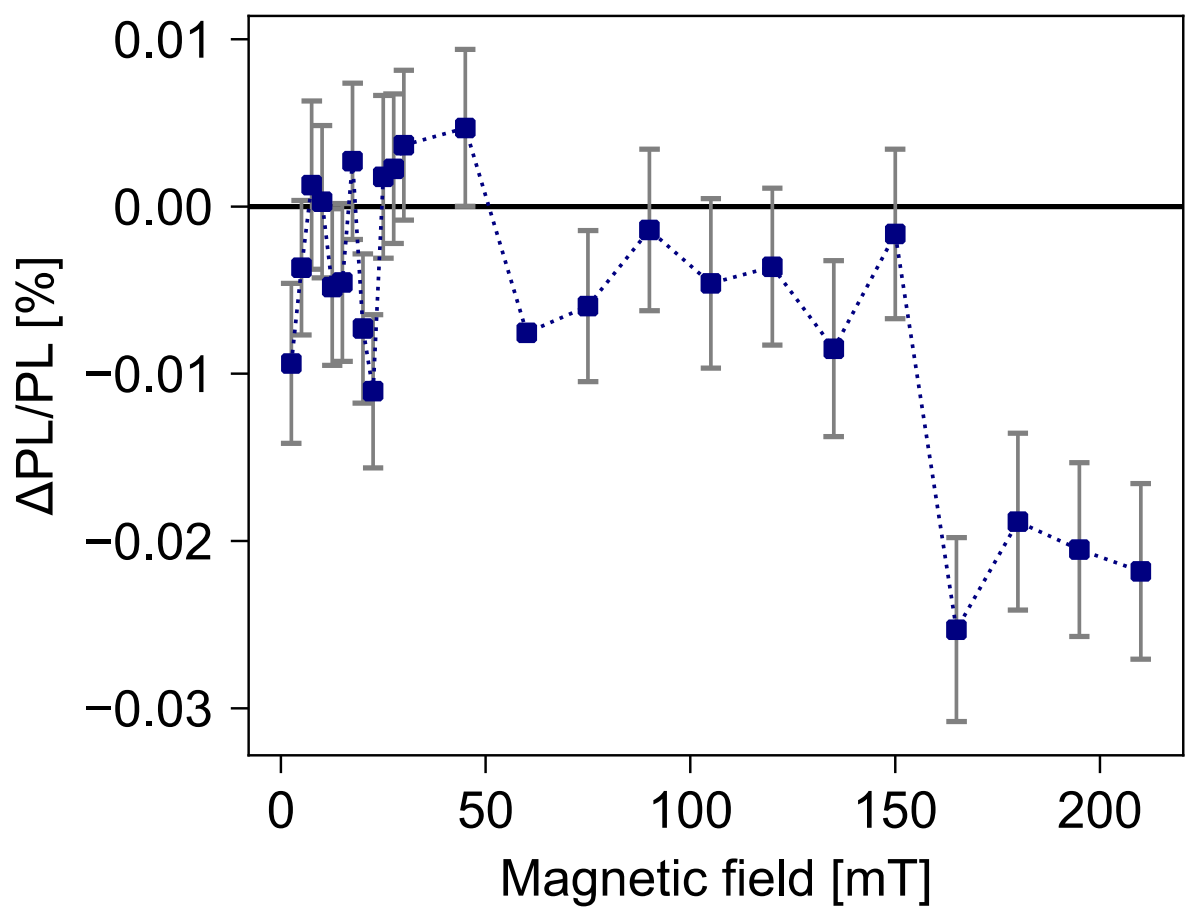

Figure S2 MPL measurements on a *c.* 5 mM solution of **4f** in dichloromethane, with an excitation wavelength of 520 nm and an emission range between 550 and 700 nm.

S3.4 Femtosecond transient absorption spectroscopy

Femtosecond transient absorption (fsTA) spectra were collected on thin-film and solution phase samples of **4a**, **4c**, **4d** and **4f**. Thin film samples were prepared by vacuum deposition of solid material onto a quartz substrate and encapsulated in a N_2 glovebox. Solution phase samples were prepared in dichloromethane at an approximate concentration of 0.1 mM in a 1 mm quartz cuvette and deoxygenated using the freeze-pump-thaw method before measurement.

The fsTA spectra were measured using a commercial instrument (Harpia TA, Light Conversion) seeded by a Nd:KGW regenerative amplifier (Pharos, Light Conversion, $\tau \approx 190$ fs, 1030 nm, $f = 50$ kHz). A portion of the beam was split before entering an optical parametric amplifier (OPA) (Orpheus-HP, Light Conversion), producing a tunable excitation ('pump') source, which was then modulated at 25 Hz by an optical chopper. The remaining 1030 nm beam was focused through a sapphire (<300-900 nm) to generate the white light supercontinuum used as 'probe'. Frequency-doubling of the seed prior to white light generation shifted the probe spectrum below 500 nm. The probe traversed a mechanical delay stage used to control its time of arrival at the sample relative to the pump. The pump and probe were then focused onto the same spot on the sample. The transmission with and without the pump was measured by a free-space spectrograph (Kymera 193i, Andor), to generate the differential absorption spectrum at each time point ($\Delta OD(\lambda, t)$). The pump was kept at a sufficiently low excitation density for all measurements so as to preclude many-body recombination processes such as singlet-singlet annihilation. Parameters used for collection of this data are given in **Table S7** and **Table S8**.

Table S7 Parameters used for collection of fsTA data on solutions of **4a**, **4c**, **4d** and **4f**.

	**4a**	**4c**	**4d**	**4f**
Excitation wavelength [nm]	410	480	520	480
Fluence [μJ cm^{-1}]	130	100	80	100

Table S8 Parameters used for collection of fsTA data on thin films of **4a**, **4c**, **4d** and **4f**.

	**4a**	**4c**	**4d**	**4f**
Excitation wavelength [nm]	410	460	480	480
Fluence [μJ cm^{-1}]	130	120	100	100

Full fsTA spectra are given below; solution-phase data is given in **Figure S3**, thin-film data is given in **Figure 5** in the main text.

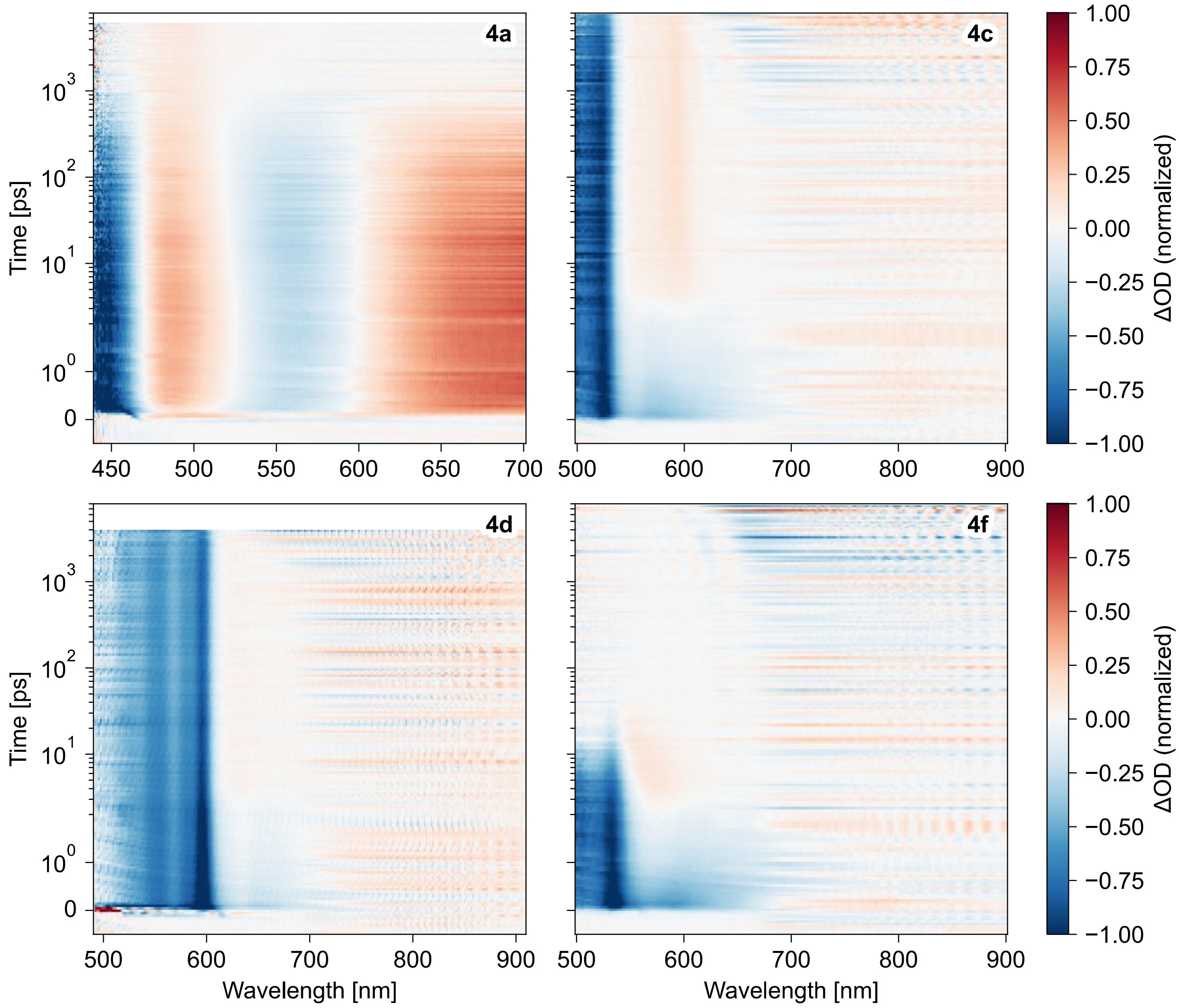

Figure S3 fsTA spectra of solutions of **4a**, **4c**, **4d** and **4f**, *c.* 0.1 mM in dichloromethane. λ_{ex}: **4a** 410 nm, **4c** 480 nm, **4d** 510 nm, **4f** 480 nm.

fsTA data were deconvoluted using the KiMoPack[6] software package to fit individual species to their species-associated spectra (SAS), using a wavelength bin size of 2 nm and fitting

between 1 ps and the end of the experiment. The SAS for solution-phase fsTA measurements are given in **Figure S4**, thin-film SAS are given in **Figure S6**.

Solution-phase sample **4a**, *c.* 0.1 mM in dichloromethane, was modelled using a mono-exponential decay fit to the ground state (*GS*):

$$A \xrightarrow{k_1} GS \qquad \text{(Model I)}$$

This model was interpreted as a simple $S_1 \rightarrow S_0$ decay with ground-state bleach (GSB) and stimulated emission (SE) features corresponding to the UV-visible absorption and emission spectra respectively.

Solution-phase samples **4c** and **4d**, *c.* 0.1 mM in dichloromethane, were modelled using a mono-exponential decay fit to a state with lifetime longer than the duration of the experiment:

$$A \xrightarrow{k_1} B \qquad \text{(Model II)}$$

This model was interpreted as an $S_1^* \rightarrow S_1/T_1$ ISC mechanism. In both cases the short-lived decay component ($\tau \leq 3$ ps) contains GSB and SE features corresponding to the UV-visible absorption and emission spectra respectively. The GSB persists into the long-lived component, but the SE feature is replaced with an excited-state absorption (ESA). Since TCSPC (**Table S4**) reveals an emissive lifetime of ~200 ps in **4c**, we assign the first component to a vibrationally hot state, followed by S_1 and T_1 states which are poorly resolved due to overlapping ESAs. We assign part of this state to T_1 based on the nanosecond TA (nsTA) experiments discussed in the following section.

Solution-phase sample **4f**, *c.* 0.1 mM in dichloromethane, was modelled using a consecutive exponential decay fit to the ground state (*GS*) with 2 components:

$$A \xrightarrow{k_1} B \xrightarrow{k_2} GS \qquad \text{(Model III)}$$

As discussed in the main text, we could not definitively assign the components of this model, though we tentatively assign component B as a short-lived triplet.

The rate constants and lifetimes given by these models are given in **Table S9**.

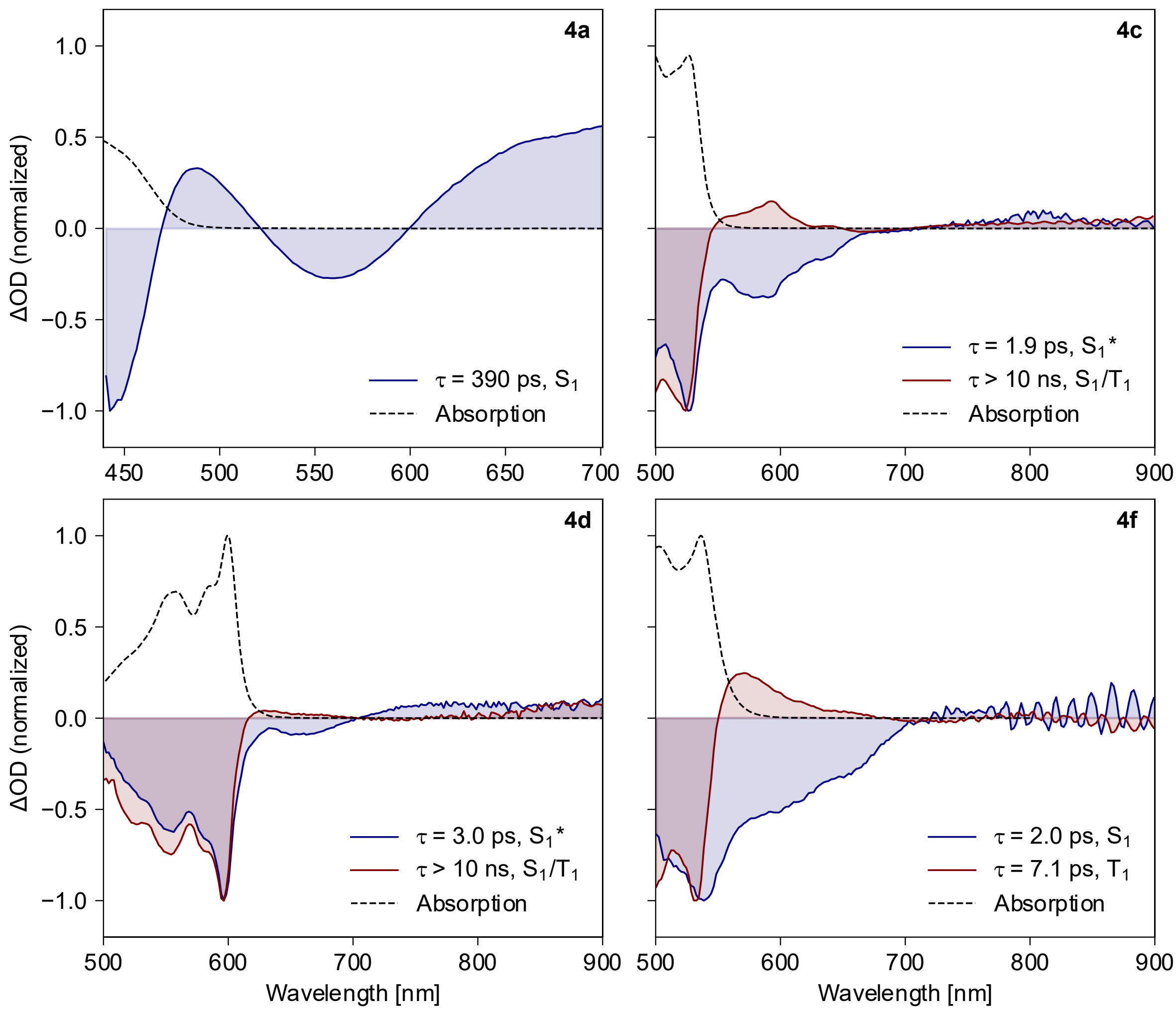

Figure S4 SAS for kinetically modelled species in the fsTA spectra of **4a**, **4c**, **4d** and **4f**, *c.* 0.1 mM in dichloromethane. λ_{ex}: **4a** 410 nm, **4c** 480 nm, **4d** 510 nm, **4f** 480 nm. Solution-phase steady-state absorption spectra are given for reference.

Table S9 Model used, rate constants k_1 and k_2 and lifetimes τ_1 and τ_2 given by consecutive mono-exponential decay fitting of solution-phase fsTA data on **4a**, **4c**, **4d** and **4f**. Errors determined using the F-test with a confidence value of 95%.

	**4a**	**4c**	**4d**	**4f**
Model	Model I	Model II	Model II	Model III
k_1 [ps^{-1}]	$(2.6^{+0.2}_{-0.2}) \times 10^{-3}$	$(5.2^{+6.5}_{-3.1}) \times 10^{-1}$	$(3.3^{+6.8}_{-2.9}) \times 10^{-1}$	$(4.9^{+21.0}_{-2.7}) \times 10^{-1}$
k_2 [ps^{-1}]	N/A	N/A	N/A	$(1.3^{+0.9}_{-0.7}) \times 10^{-1}$
τ_1 [ps]	$(3.9^{+0.3}_{-0.3}) \times 10^{2}$	$1.9^{+2.8}_{-1.1}$	$3.0^{+22.3}_{-2.0}$	$2.0^{+2.4}_{-1.6}$
τ_2 [ps]	N/A	N/A	N/A	$7.1^{+7.6}_{-2.7}$

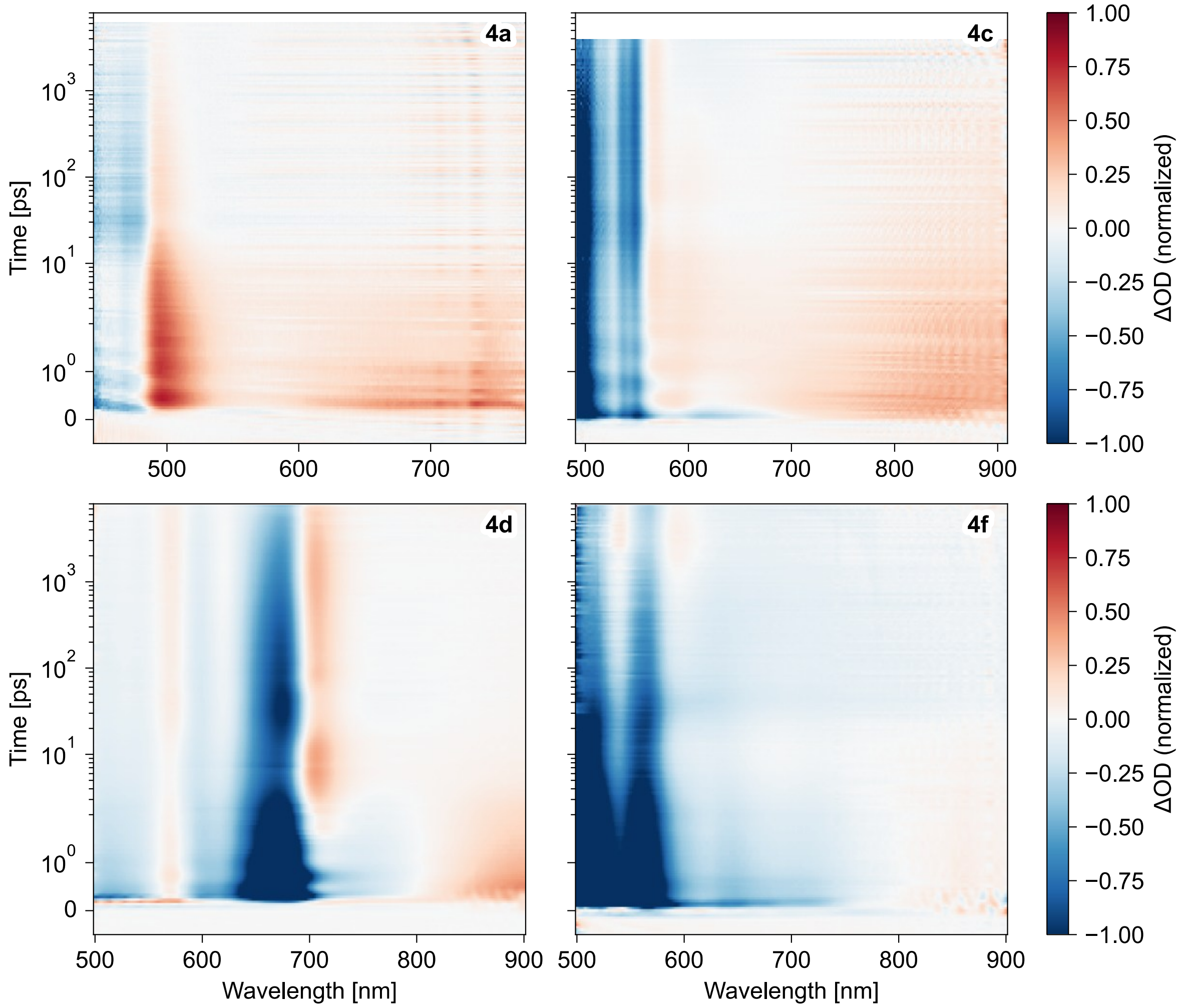

Figure S5 fsTA spectra of thin films of **4a**, **4c**, **4d** and **4f**. λ_{ex}: **4a** 410 nm, **4c** 460 nm, **4d** 480 nm, **4f** 480 nm.

fsTA measurements of a thin-film sample of **4a** were modelled using a 2-component consecutive exponential decay fit to the ground state (*GS*) with 2 components:

$$A \overset{k_1}{\rightarrow} B \overset{k_2}{\rightarrow} GS \qquad \text{(Model III)}$$

This model was interpreted as an $S_1* \rightarrow S_1 \rightarrow S_0$ mechanism, where the first component is a vibrationally hot state.

fsTA measurements of thin-film samples of **4c**, **4d** and **4f** were modelled using a 2-component consecutive exponential decay fit to a state with lifetime longer than the duration of the experiment:

$$A \overset{k_1}{\rightarrow} B \overset{k_2}{\rightarrow} C \qquad \text{(Model IV)}$$

This model was interpreted as an $S_1^* \rightarrow S_1 \rightarrow T_1$ ISC mechanism, where the first component is a vibrationally hot state.

The rate constants and lifetimes given by these models are given in **Table S10**.

Table S10 Model used, rate constants k_1 and k_2 and lifetimes τ_1 and τ_2 given by consecutive mono-exponential decay fitting of thin-film fsTA data on **4a**, **4c**, **4d** and **4f**. Errors determined using the F-test with a confidence value of 95%.

	**4a**	**4c**	**4d**	**4f**
Model	Model III	Model IV	Model IV	Model IV
k_1 [ps^{-1}]	$(9.9^{+1.5}_{-1.3}) \times 10^{-2}$	$(1.1^{+0.4}_{-0.3}) \times 10^{-1}$	$(8.5^{+1.8}_{-1.3}) \times 10^{-1}$	$(2.3^{+1.1}_{-0.9}) \times 10^{-1}$
k_2 [ps^{-1}]	$(5.4^{+1.6}_{-1.3}) \times 10^{-4}$	$(5.8^{+4.9}_{-3.8}) \times 10^{-4}$	$(9.0^{+3.4}_{-2.9}) \times 10^{-4}$	$(2.5^{+1.1}_{-0.8}) \times 10^{-2}$
τ_1 [ps]	$(1.0^{+0.2}_{-0.1}) \times 10^{1}$	$9.1^{+3.4}_{-2.5}$	$1.2^{+0.2}_{-0.2}$	$4.4^{+2.8}_{-1.4}$
τ_2 [ps]	$(1.8^{+0.6}_{-0.4}) \times 10^{3}$	$(1.7^{+3.1}_{-0.8}) \times 10^{3}$	$(1.1^{+0.5}_{-0.3}) \times 10^{3}$	$(4.0^{+1.8}_{-1.2}) \times 10^{2}$

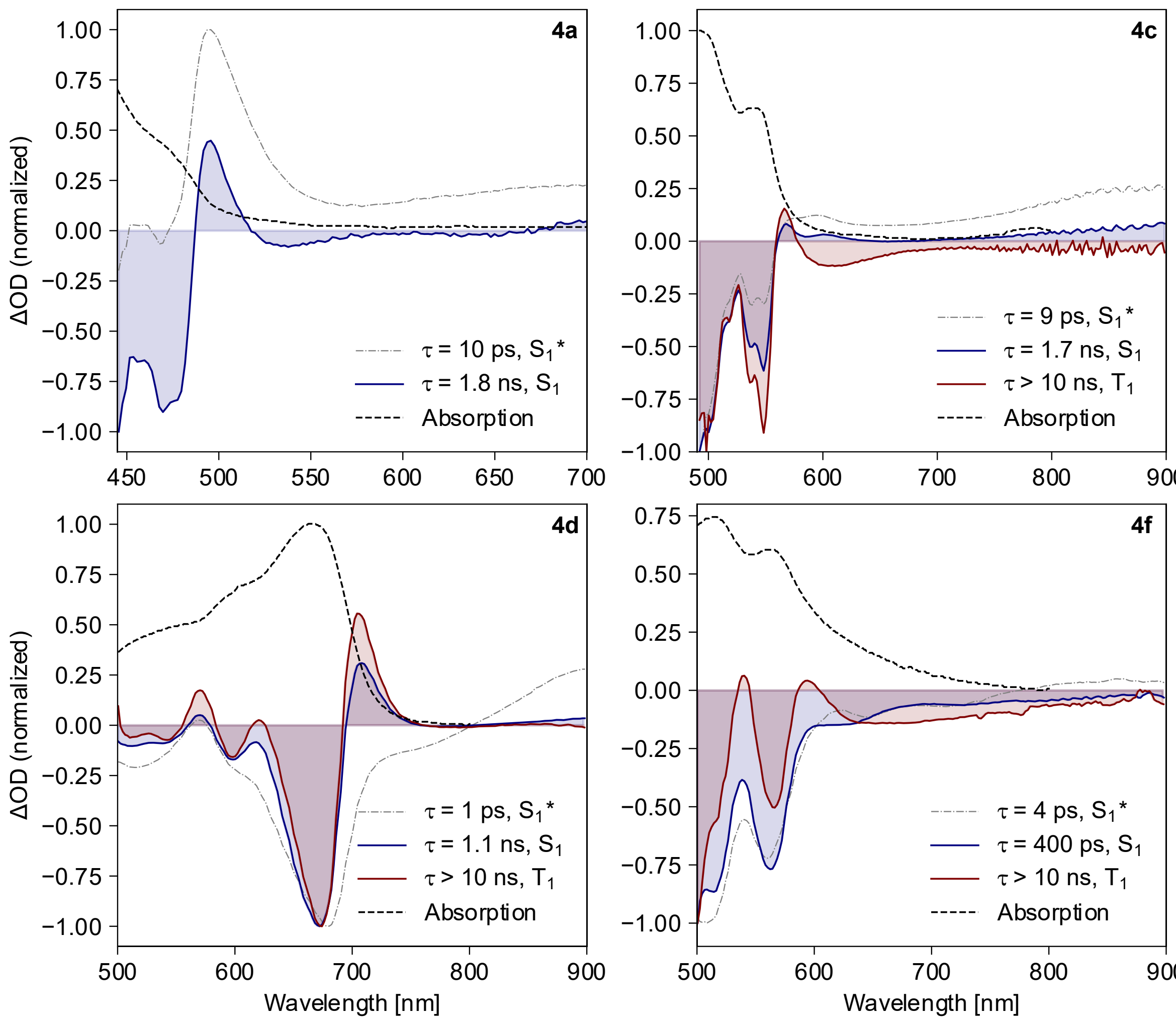

Figure S6 SAS for kinetically modelled species in the fsTA spectra of thin films of **4a**, **4c**, **4d** and **4f**. λ_{ex}: **4a** 410 nm, **4c** 460 nm, **4d** 480 nm, **4f** 480 nm. Thin film steady-state absorption spectra are given for reference. The SAS assigned as T_1 corresponds to the non-decaying component in the TA spectra.

S3.5 Nanosecond transient absorption spectroscopy

Nanosecond transient absorption (nsTA) spectra of **4c** and **4d**, *c.* 0.1 mM in dichloromethane, were collected using a Magnitude Instruments enVISion 5.3 UV/visible/NIR transient absorption spectrometer according to parameters given in **Table S11**.

Table S11 Parameters used for collection of solution-phase nsTA data on **4c** and **4d**.

	**4c**	**4d**
Probe wavelength [nm]	520	595
Spectral resolution [nm]	10	10
Repetition rate [Hz]	5556	5556
Excitation wavelength [nm]	470	520
Excitation fluence [μJ cm^{-2}]	40	25

Data were fitted to extract rate constants using the lmfit *Minimizer* function.[7] Data from samples **4c** and **4d** were modelled using a mono-exponential decay fit to the ground state (*GS*):

$$A \xrightarrow{k_1} GS \qquad \text{(Model I)}$$

This model was interpreted as a $T_1 \rightarrow S_0$ mechanism.

Table S12 Model, rate constants k_I and lifetimes τ_I given by mono-exponential decay fitting of solution-phase nsTA data on **4c** and **4d**. Errors determined using the F-test with a confidence value of 95%.

	**4c**	**4d**
Model	Model I	Model I
k_I [ns^{-1}]	$(2.58^{+0.03}_{-0.03}) \times 10^{-4}$	$(3.35^{+0.02}_{-0.02}) \times 10^{-4}$
τ_I [ns]	$(3.87^{+0.04}_{-0.04}) \times 10^{3}$	$(2.98^{+0.02}_{-0.02}) \times 10^{3}$

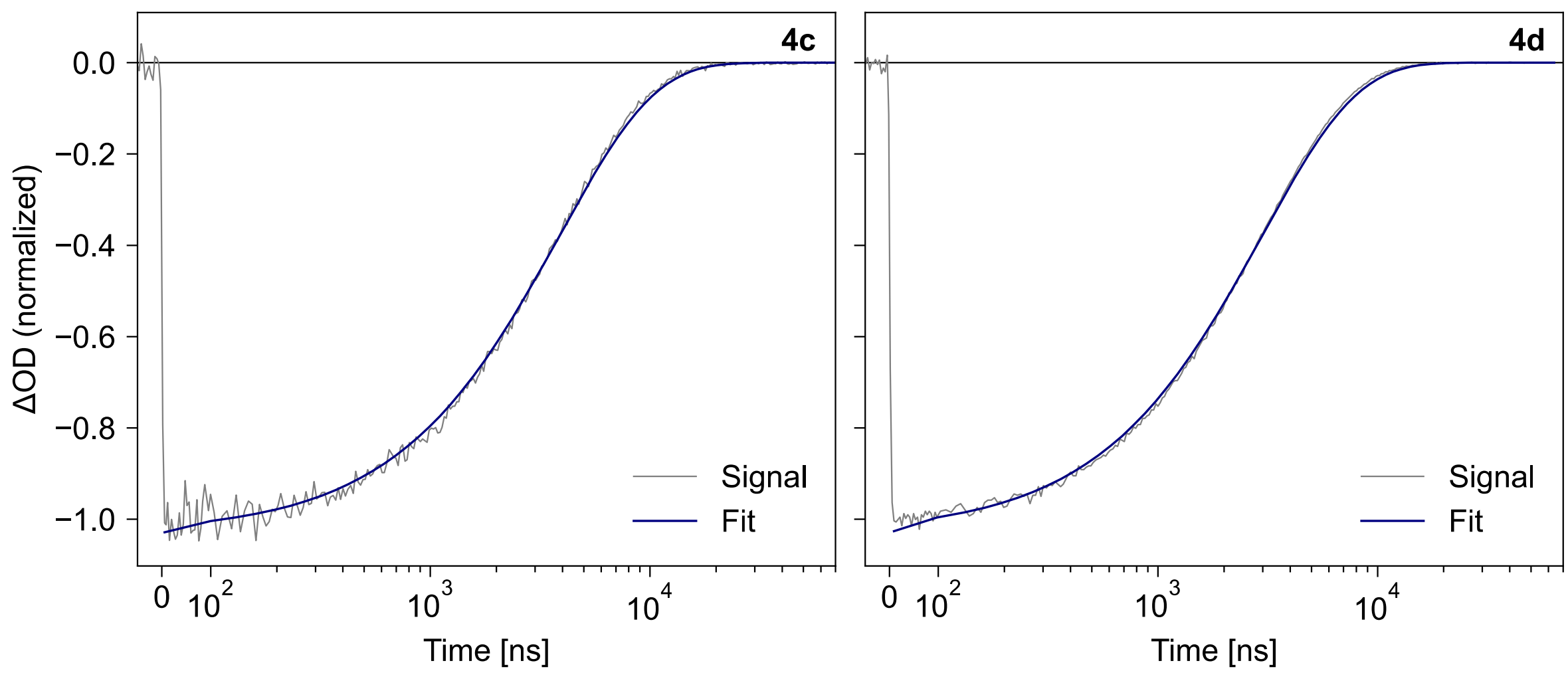

Figure S7 Solution-phase nsTA spectroscopic data for **4c** and **4d**, *c.* 0.1 mM in dichloromethane. Data fitted to consecutive exponential decay models according to parameters described in **Table S12**.

Nanosecond transient absorption (nsTA) spectra of thin films were collected using a Magnitude Instruments enVISion 5.3 UV/visible/NIR transient absorption spectrometer according to parameters given in **Table S13**.

Table S13 Parameters used for collection of nsTA data on thin films of **4c**, **4d** and **4f**.

	**4c**	**4d**	**4f**
Probe wavelength [nm]	500	675	560
Spectral resolution [nm]	10	9.6	9.6
Repetition rate [Hz]	5600	1000	1000
Excitation wavelength [nm]	460	480	480
Timescale [µs]	32	16	16

Data were fit to extract rate constants using the lmfit *Minimizer* function.[7] Data from samples **4c** and **4f** were modelled using a consecutive, mono-exponential decay fit with 2 components:

$$A \overset{k_1}{\rightarrow} B \overset{k_2}{\rightarrow} GS \qquad \text{(Model III)}$$

This model was interpreted as an $S_1 \rightarrow T_1 \rightarrow S_0$ mechanism.

Data from sample **4d** were modelled using a consecutive, mono-exponential decay fit with 3 components:

$$A \overset{k_1}{\rightarrow} B \overset{k_2}{\rightarrow} C \overset{k_3}{\rightarrow} GS \qquad \text{(Model V)}$$

We proposed that component C is a triplet due to its lifetime, but we could not confidently assign component B using the data available.

Table S14 Model, rate constants $k_1 \cdots k_3$ and lifetimes $\tau_1 \cdots \tau_3$ given by consecutive mono-exponential decay fitting of nsTA data on thin films of **4c**, **4d** and **4f**. Errors determined using the F-test with a confidence value of 95%.

	**4c**	**4d**	**4f**
Model	Model III	Model V	Model III
k_1 [ns^{-1}]	$(5.1^{+0.6}_{-0.4}) \times 10^{-1}$	$(7.2^{+1.5}_{-1.3}) \times 10^{-2}$	$(2.7^{+1.5}_{-1.0}) \times 10^{-1}$
k_2 [ns^{-1}]	$(9.0^{+0.8}_{-0.7}) \times 10^{-4}$	$(2.2^{+0.1}_{-0.1}) \times 10^{-3}$	$(4.8^{+1.1}_{-0.9}) \times 10^{-1}$
k_3 [ns^{-1}]	N/A	$(7.0^{+0.7}_{-0.7}) \times 10^{-5}$	N/A
τ_1 [ns]	$1.9^{+0.2}_{-0.2}$	$(1.4^{+0.3}_{-0.2}) \times 10^{1}$	$3.7^{+2.0}_{-1.3}$
τ_2 [ns]	$(1.1^{+0.1}_{-0.1}) \times 10^{3}$	$(4.6^{+0.2}_{-0.2}) \times 10^{2}$	$(2.1^{+0.5}_{-0.4}) \times 10^{2}$
τ_3 [ns]	N/A	$(1.4^{+0.2}_{-0.1}) \times 10^{4}$	N/A

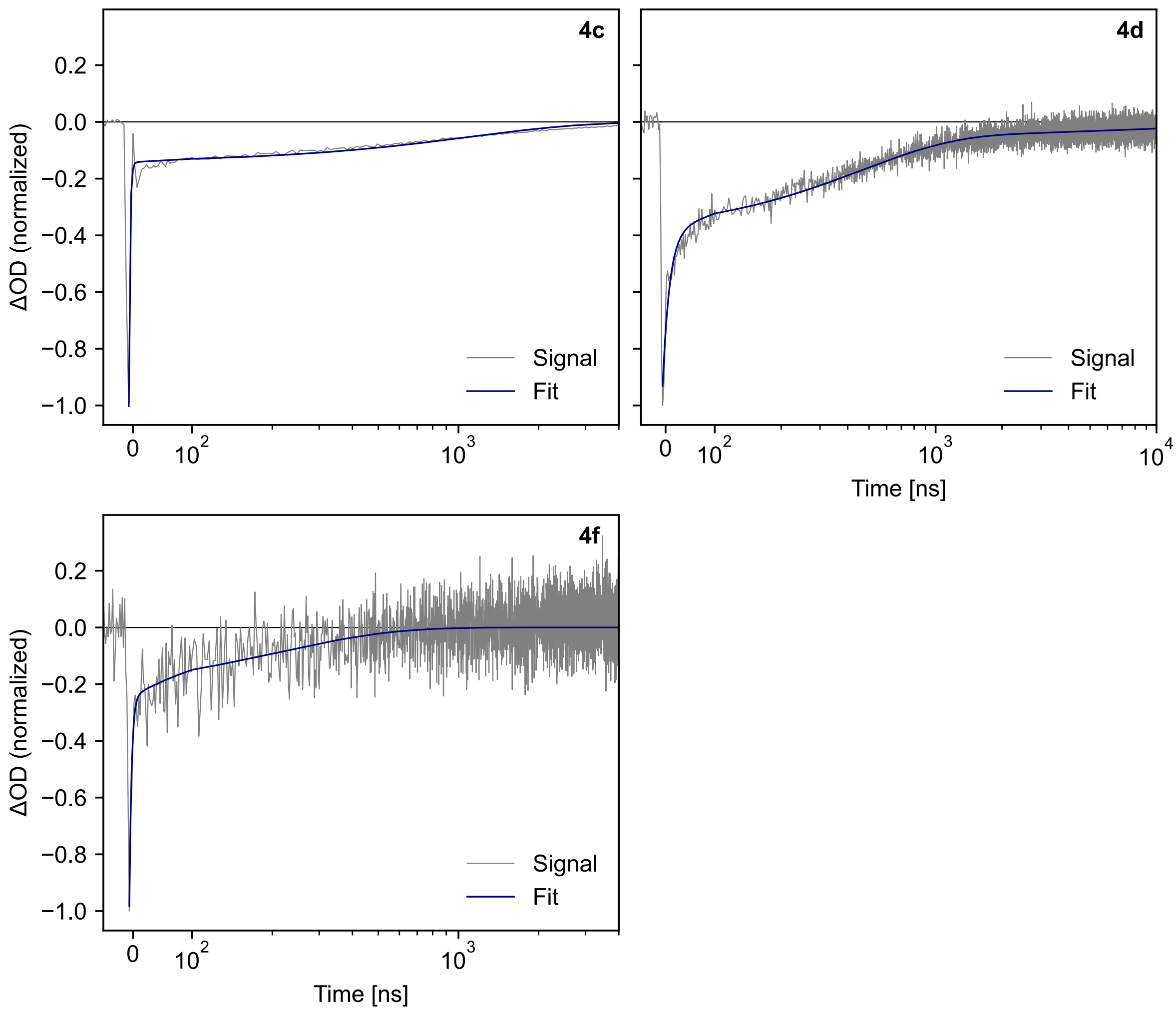

Figure S8 Nanosecond TA spectroscopic data for thin films of **4c**, **4d** and **4f**. Data fit to consecutive exponential decay models according to parameters described in **Table S14**.

S3.6 Transient electron paramagnetic resonance spectroscopy

A sample of **4f** was thermally evaporated onto a glass slide, then finely pulverized and added to a quartz EPR tube in an inert atmosphere. Transient electron paramagnetic resonance spectroscopy (TR-EPR) measurements on this sample were performed using a Bruker E580 Electron Spin Resonance (ESR) system. We used an X-band microwave source (Bruker, Super X FT-EPR Bridge) and resonator (Bruker, MD5). During the measurement the sample and resonator are cooled using a helium cryostat (Oxford Instruments, CF935) which includes a window for optical access. We used a tunable ~7 ns pulsed laser (Opotek, Opolette) for excitation and reported times are relative to the Q-switch of the laser. Transients are recorded using a fast digitizer (Bruker, SpecJet II) and a delay generator (Stanford Research, DG645) to trigger collection 200 ns before the Q-switch to allow for background subtraction. The time resolution of our transient spectra was 4 ns. Parameters used for collection of this data are given in **Table S15**. Data collected at 40 K is given in **Figure S9**; 80 K is given in **Figure S10**.

Table S15 Parameters used for collection of thin-film TR-EPR data on **4f**.

**Temperature [K]**	**40**	**80**
Excitation wavelength [nm]	510	510
Receiver gain [dB]	25	25
Microwave frequency [GHz]	9.75	9.75
Trigger position [µs]	1.0	0.5
Magnetic field span [mT]	279–420	289–410
Time span [µs]	15.4	15.9

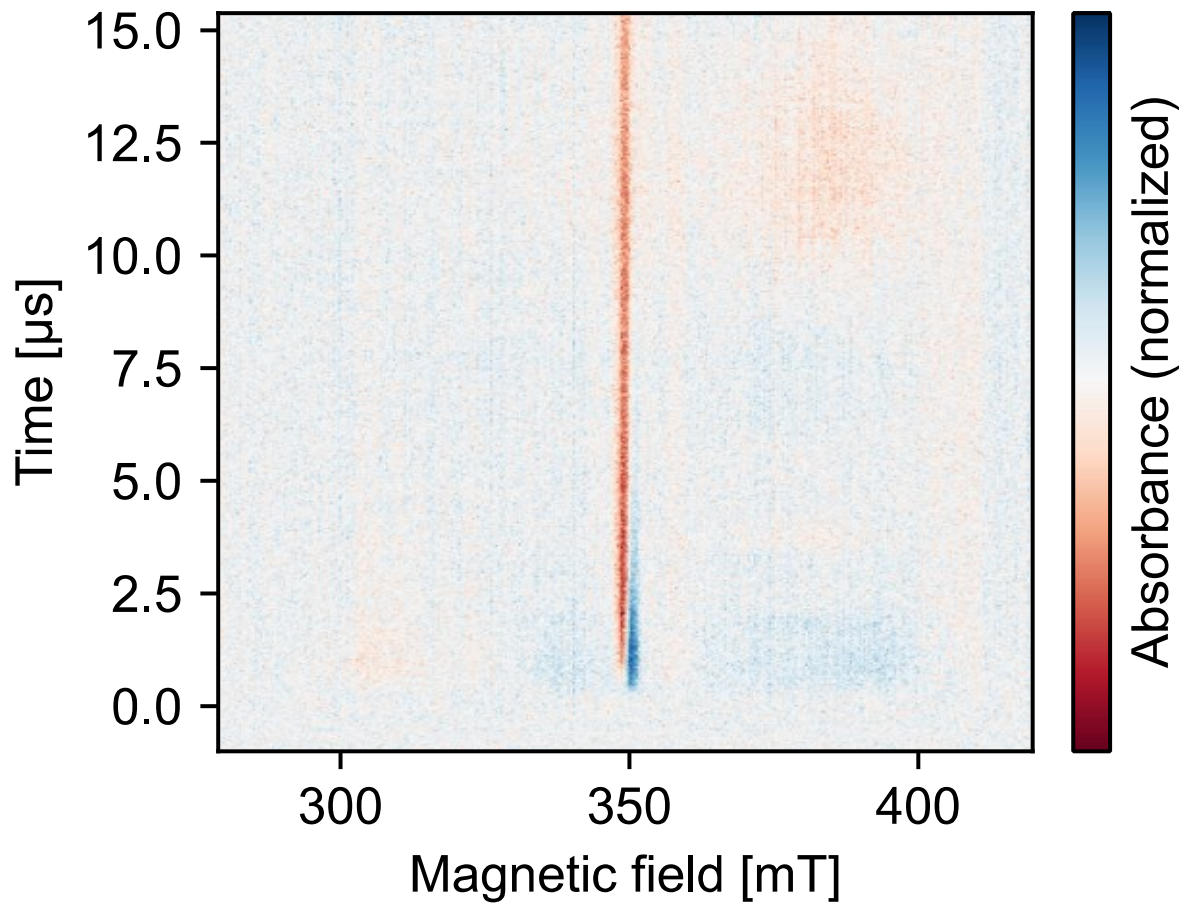

Figure S9 Continuous-wave TR-EPR spectra of **4f** pulverized thin film at 40 K. λ_{ex}: 510 nm.

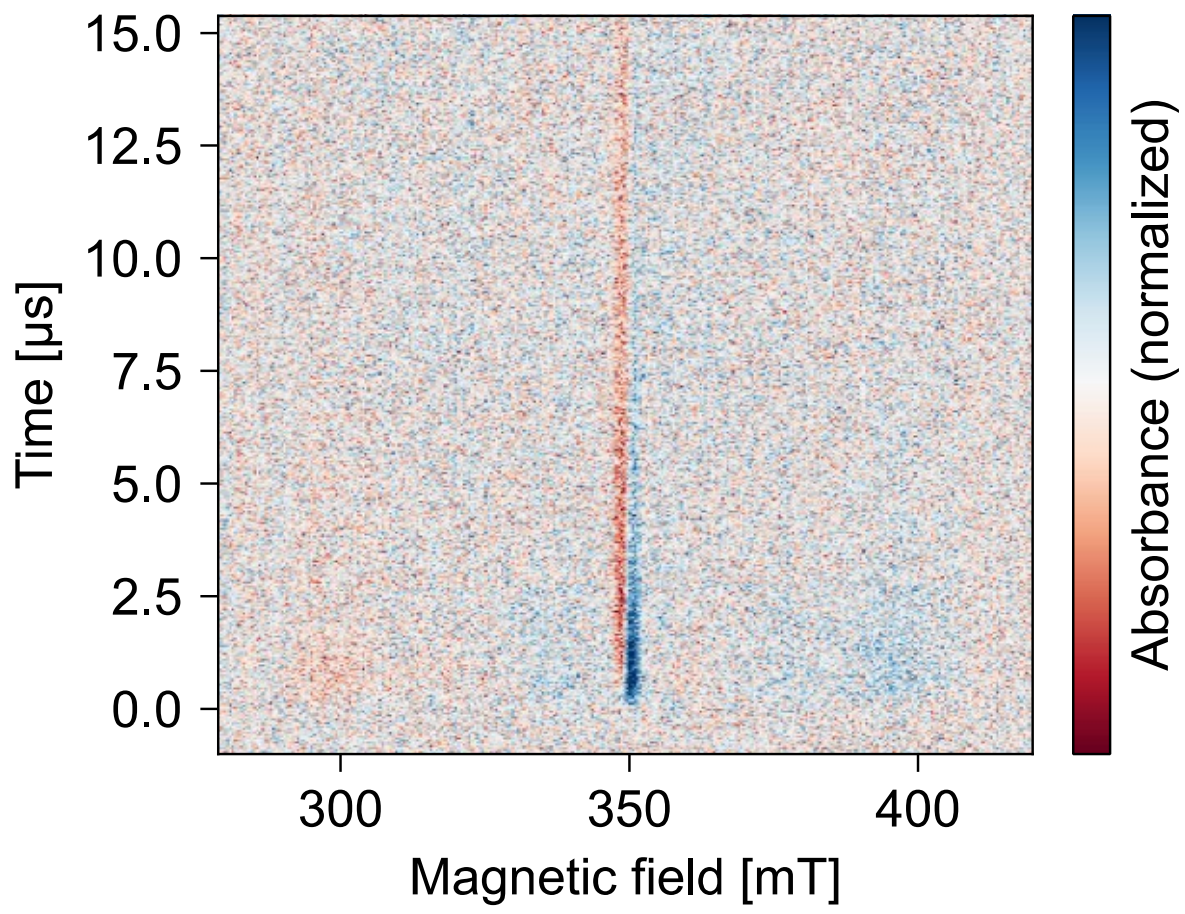

Figure S10 Continuous-wave TR-EPR spectra of **4f** pulverized thin film at 80 K. λ_{ex}: 510 nm.

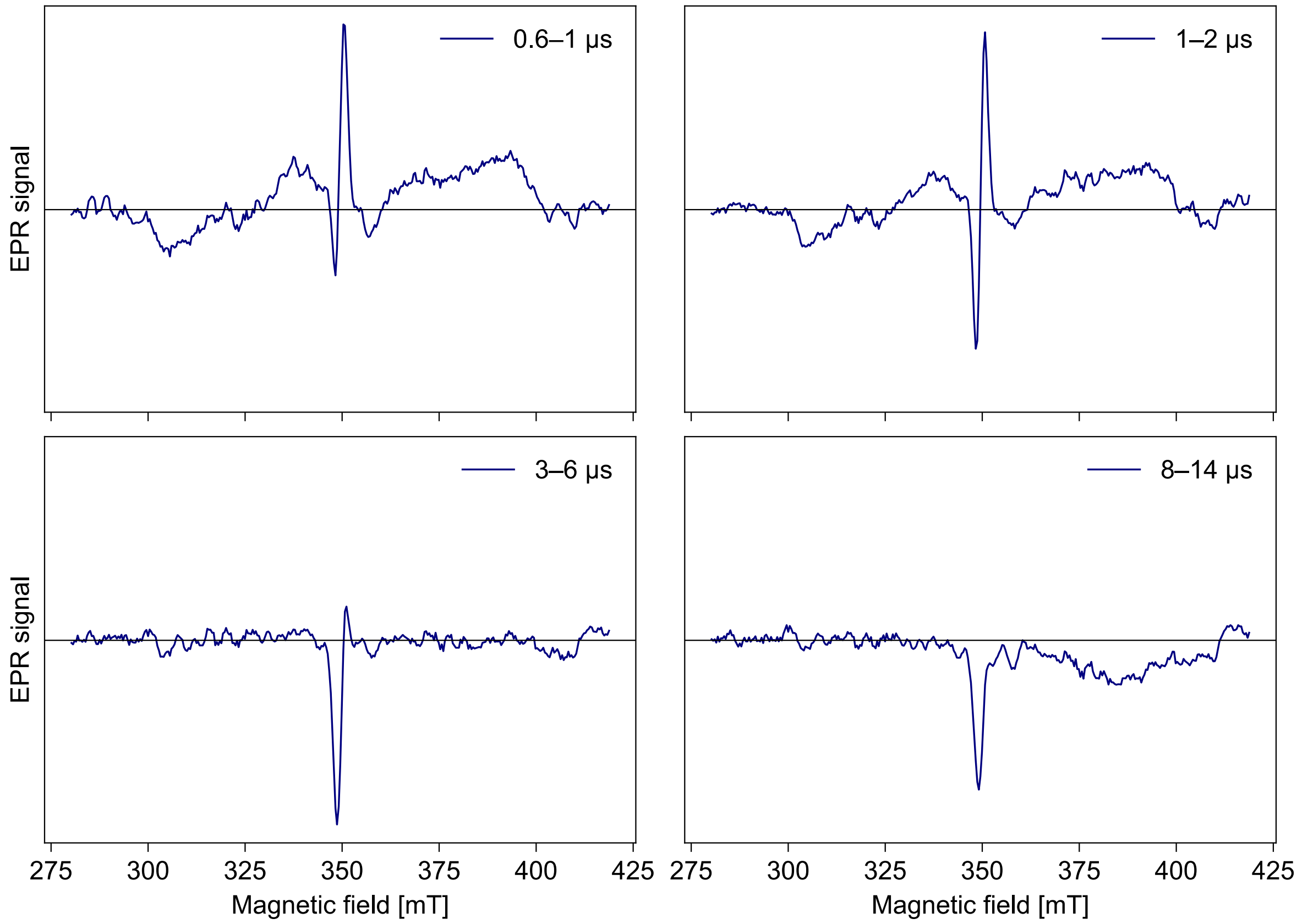

Figure S11 Continuous-wave TR-EPR spectrum of **4f** as a pulverized thin film at 40 K, at various time points after excitation; line smoothed using a 5-point moving average. λ_{ex}: 510 nm.

S3.7 Time-resolved photoluminescence spectroscopy

Time-resolved photoluminescence (TRPL) spectroscopy was conducted on a thin film sample of **4f**. This sample was prepared by vacuum deposition of solid material onto a quartz substrate and encapsulated in a N_2 glovebox. TRPL was conducted using 500 nm excitation from the same OPA as in **S3.4**. Emission was collected by a pair of off-axis parabolic mirrors, passing through a 550 nm longpass filter to reject scattered excitation light. The spectrum was analysed by a spectrograph (Acton SpectraPro SP-2150, Princeton Instruments) coupled to a high-speed intensified charge-coupled device camera (Pi-Max 4, Princeton Instruments, 300 g/mm, 500 nm blaze). Spectra were gated electronically with respect to the laser source trigger to construct the time-axis (time resolution of ~1.5 ns). The kinetics were generated by integrating the PL spectrum between 550 and 800 nm.

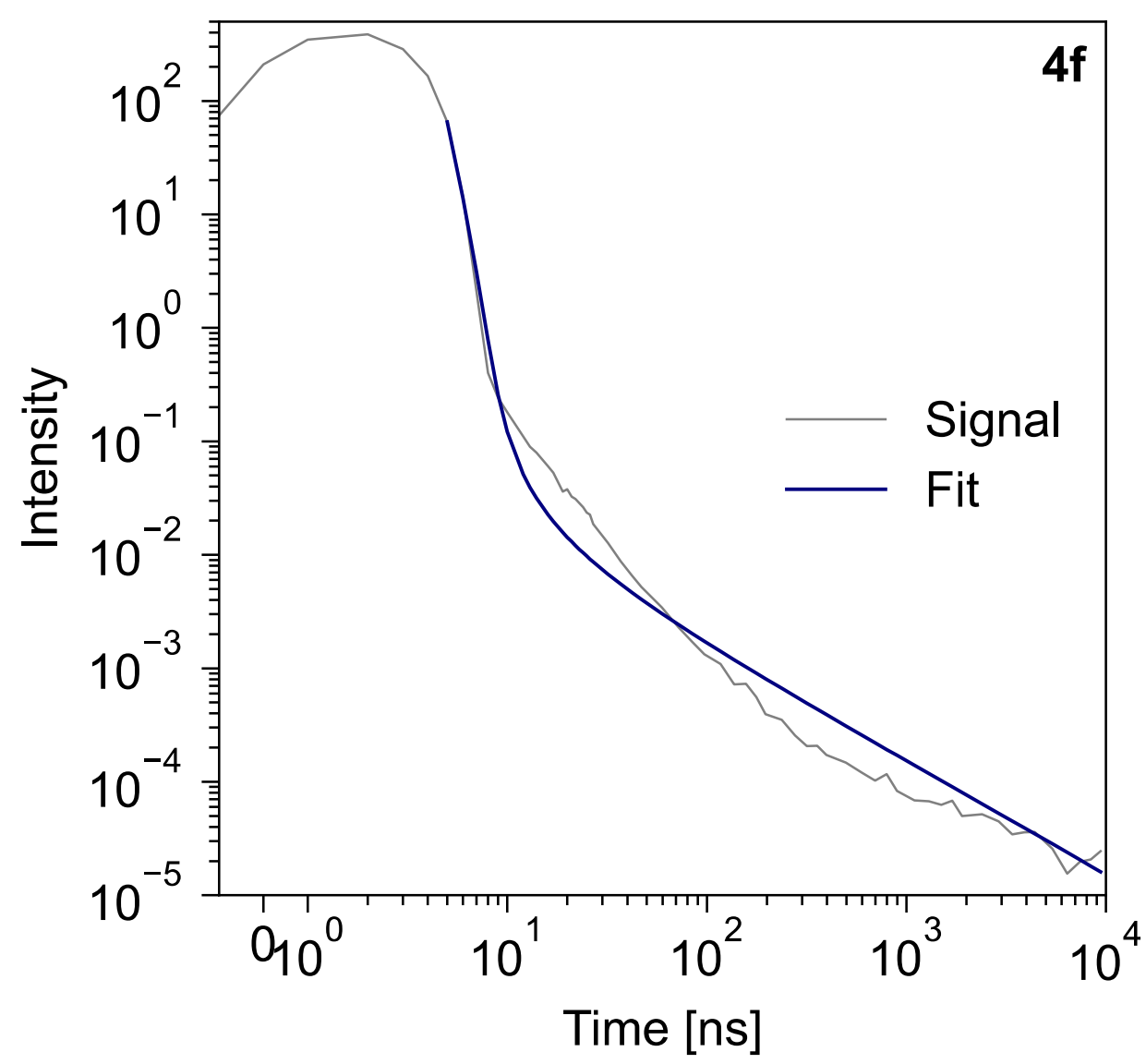

Figure S12 Thin film long-time time-resolved photoluminescence data for **4f**. Data fitted to an ISC → TTA-UC model as described below.

From the time-resolved photoluminescence data of **4f** in **Figure S12**, a fit was performed using the lmfit *Minimizer* function.[7] Data were modelled to the following process:

$$A \overset{k_1}{\rightarrow} B$$

$$B + B \overset{k_2}{\rightarrow} A \qquad \text{(Model VI)}$$

$$A \overset{k_3}{\rightarrow} GS$$

Data were fitted between $t = 5$ and 10 000 ns to exclude the initial 'S_1*' state, which is not captured in this model. The rate constants and lifetimes are given in **Table S16**.

Table S16 Model, rate constants $k_1 \cdots k_3$ and lifetimes $\tau_1 \cdots \tau_3$ given ISC → TTA-UC model (Model VI) for TRPL data on **4f**.

	**Value**
k_1 [ns^{-1}]	2.2
k_2 [ns^{-1}]	1.1×10^{1}
k_3 [ns^{-1}]	3.1
τ_1 [ns]	4.5×10^{-1}
τ_2 [ns]	8.8×10^{-2}
τ_3 [ns]	3.2×10^{-1}

S4. Crystallography

S4.1 Experimental details

SAINT v8.37A,[8] XL,[9] SHELXT 2018/2,[10] and Olex2 1.5-alpha[11] were used for data collection and refinement. ORTEP was used for the below visualization.[12] Mercury[13] and UCSF Chimera[14] were used to measure and visualize the distance between planes. Parameters are given for isopyrocoll **4a** and side product **5** in **Table S17** and **Table S18** respectively.

Table S17 Parameters used for collection and refinement of isopyrocoll **4a** crystal data.

Crystal data	
Chemical formula	$C_{16}H_{18}N_2O_2$
M_r	270.32
Crystal system, space group	Monoclinic, $P2_1/n$
Temperature [K]	120
a, b, c [Å]	4.5376 (2), 21.7323 (8), 7.3096 (3)
β [°]	104.166 (1)
V [Å^3]	698.90 (5)
Z	2
Radiation type	Mo *K*α
μ [mm^{-1}]	0.09
Crystal size [mm]	0.33 × 0.09 × 0.05
Data collection	
Diffractometer	Bruker D8 Quest
Absorption correction	Multi-scan<br>*SADABS2016*/2 (Bruker,2016/2) was used for absorption correction. wR2(int) was 0.1456 before and 0.0737 after correction. The Ratio of minimum to maximum transmission is 0.8393. The λ/2 correction factor is Not present.
T_{min}, T_{max}	0.626, 0.745
No. of measured, independent and observed [$I > 2\sigma(I)$] reflections	19723, 1441, 1185
R_{int}	0.052
$(\sin \theta/\lambda)_{max}$ [Å^{-1}]	0.627
Refinement	
$R[F^2 > 2\sigma(F^2)]$, $wR(F^2)$, S	0.054, 0.143, 1.20
No. of reflections	1441
No. of parameters	93
H-atom treatment	H-atom parameters constrained
$\Delta\rho_{max}$, $\Delta\rho_{min}$ [e Å^{-3}]	0.24, −0.35

Table S18 Parameters used for collection and refinement of side product **5** crystal data.

Crystal data	
Chemical formula	$C_{14}H_{11}N_5$
M_r	249.28
Crystal system, space group	Monoclinic, $P2_1/n$
Temperature [K]	296
a, b, c [Å]	4.411 (4), 22.56 (2), 12.793 (12)
β [°]	98.958 (19)
V [Å^3]	1257.6 (19)
Z	4
Radiation type	Mo $K\alpha$
μ [mm^{-1}]	0.09
Crystal size [mm]	0.020×0.026 ×0.16
Data collection	
Diffractometer	Bruker *APEX*-II CCD
Absorption correction	Multi-scan<br>*SADABS2016*/2 (Bruker,2016/2) was used for absorption correction. wR2(int) was 0.1586 before and 0.1011 after correction. The Ratio of minimum to maximum transmission is 0.5391. The λ/2 correction factor is Not present.
T_{min}, T_{max}	0.402, 0.745
No. of measured, independent and observed [$I > 2\sigma(I)$] reflections	29636, 2074, 1447
R_{int}	0.095
$(\sin \theta/\lambda)_{max}$ [Å^{-1}]	0.582
Refinement	
$R[F^2 > 2\sigma(F^2)]$, $wR(F^2)$, S	0.052, 0.143, 1.05
No. of reflections	2074
No. of parameters	188
H-atom treatment	3
$\Delta\rho_{max}$, $\Delta\rho_{min}$ [e Å^{-3}]	H atoms treated by a mixture of independent and constrained refinement
$R[F^2 > 2\sigma(F^2)]$, $wR(F^2)$, S	0.24, −0.28

S4.2 Crystal structures

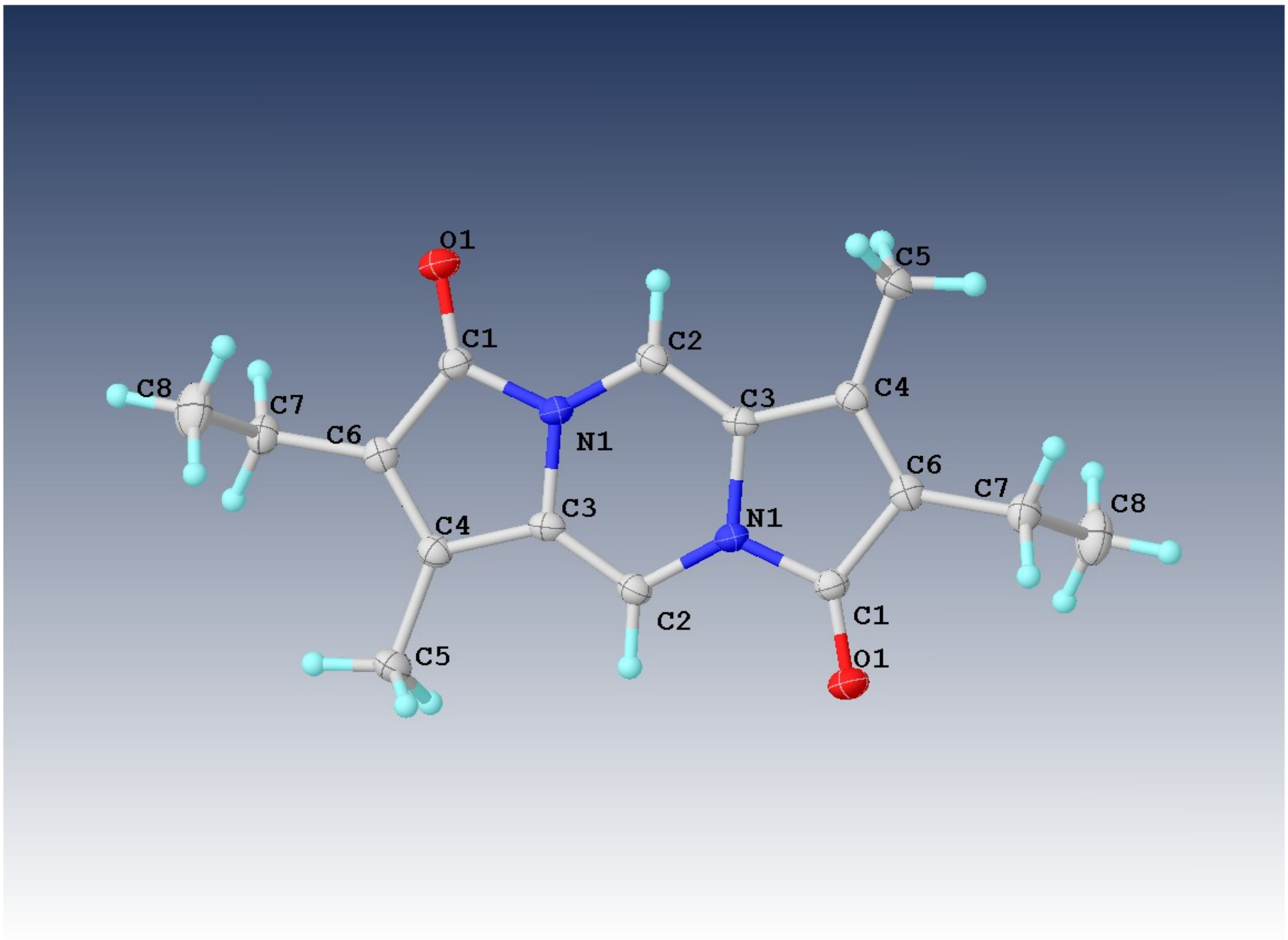

Figure S13 X-ray crystal structure of **4a**. Ellipsoids displayed at 50% probability, hydrogen atoms displayed as fixed spheres.

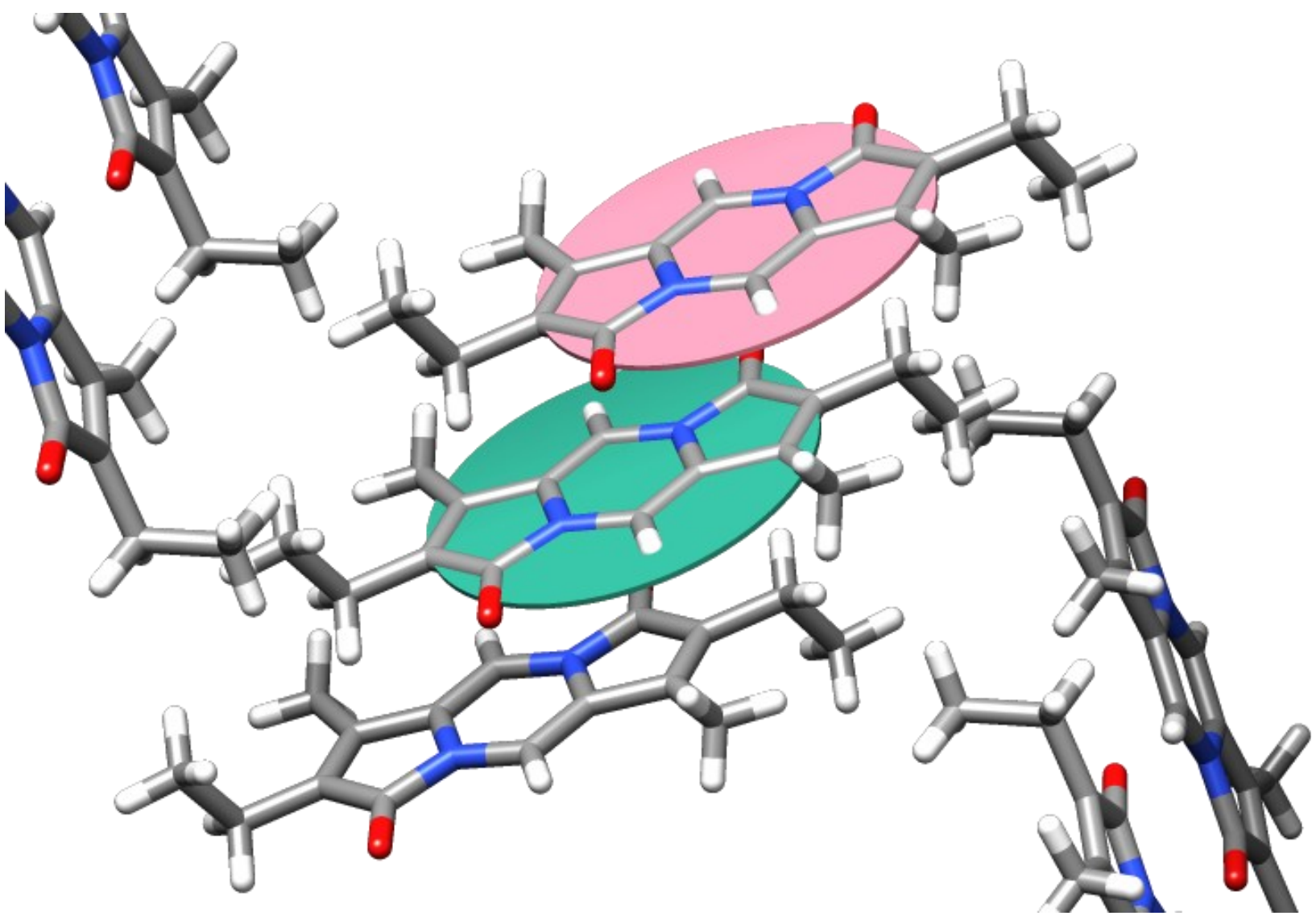

Figure S14 Crystal packing of **4a** showing 3.4 Å distance between planes.

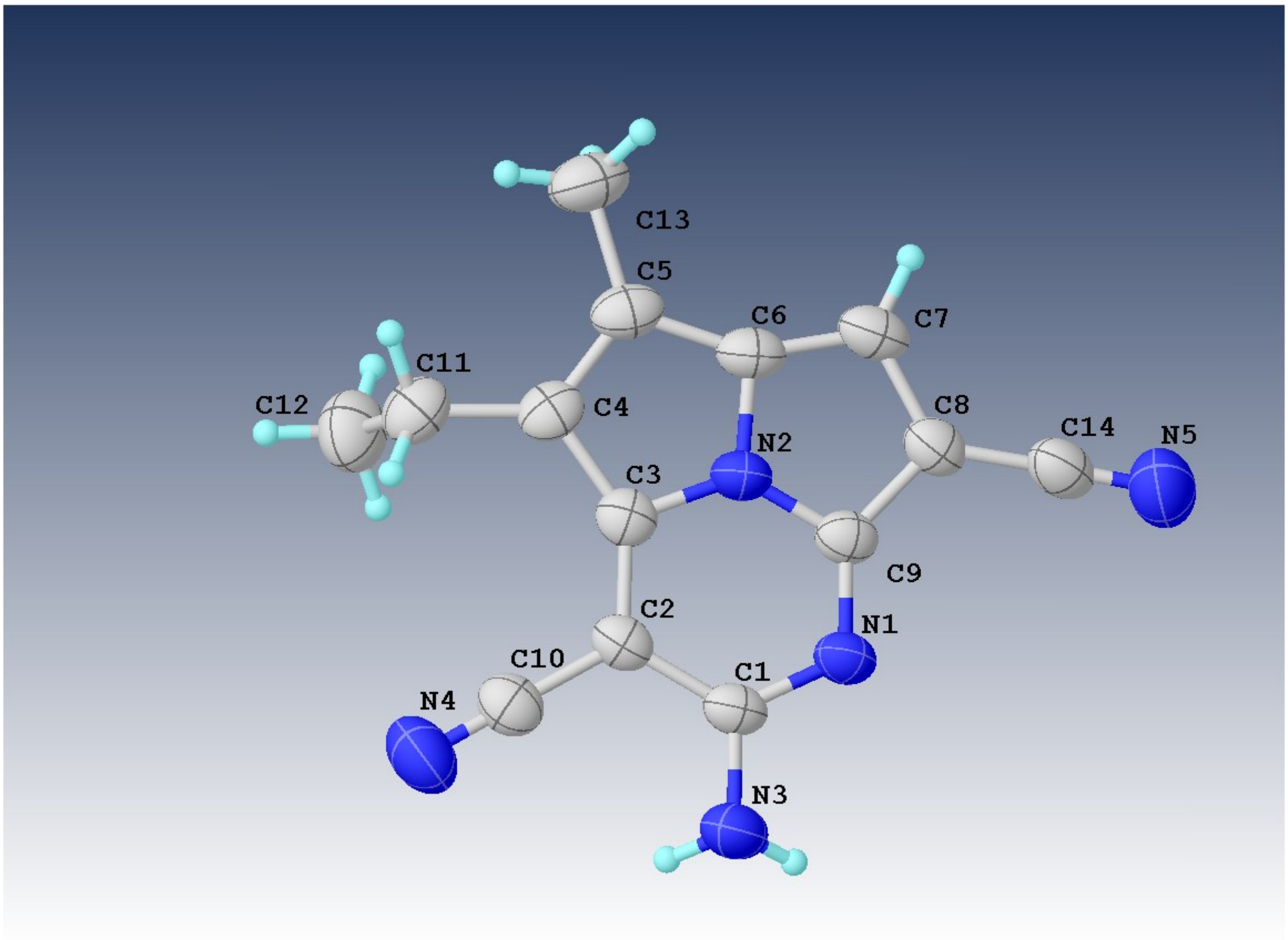

Figure S15 X-ray crystal structure of **5**. Ellipsoids displayed at 50% probability, hydrogen atoms displayed as fixed spheres.

S5. Computational

S5.1 General procedures

Density functional theory (DFT) calculations were performed using Gaussian16.[15] Starting geometries were computed using semi-empirical methods (GFN2-xTB),[16] then the molecular geometry was optimized using the 6-311G(d,p) basis set[17,18] and the B3LYP functional[19] unless otherwise described, with tight convergence criteria and ultrafine integration. The results were checked for all real vibrational frequencies and wavefunction stability. $S_0 \rightarrow S_n$ transition energies were calculated using time-dependent DFT (TD-DFT) using 32 excited states. The $S_0 \rightarrow T_1$ transition energy was calculated using three methods:

- Single-point energy calculation of the triplet configuration at the ground state geometry, given as $E(T_1)_v$ (vertical excitation),
- Single-point energy calculation of the optimized triplet geometry, given as $E(T_1)_a$ (adiabatic),
- TD-DFT using 32 excited states given as $E(T_1)_t$ (TD-DFT).

Natural bond order (NBO) and natural population analysis (NPA) for electron density of delocalized bonds (EDDB) calculations were performed using NBO 7.0.[20,21] Natural spin density (NSD) values for each atom given in **Figure S27** to **Figure S30** were obtained using NBO as above. EDDB version 11-Feb-2024 by Szczepanik and co-workers[22–24] was used with R version 4.5.2.[25] When analyzing Wiberg bond orders, we assume that any Wiberg bond index greater than 1, or 0.5 spin-separated, is attributable to π-bond order, and that lone pairs in the central rings are part of the π-system.

S5.2 Benchmarking of computational methods

The functional and basis set were benchmarked against experimental ^{1}H NMR and UV-visible data for structures **4a**, **4c**, **4d**, and **4f**. A variety of hybrid functionals (B3LYP,[19] PBE0,[26] TPSSh[27,28]) and one range-separated functional (ωB97XD[29]) were tested. One each of Pople, Dunning and Karlsruhe triple zeta basis sets were chosen: 6-311G(d,p),[17,18] cc-pVTZ,[30] and def2-TZVP.[31] Structures were optimized using the functionals and basis sets above, and solvent effects were approximated using the polarizable continuum model (PCM) with the integral equation formalism variant and the parameters for chloroform.[32] NMR

chemical shifts were calculated using the GIAO (gauge-independent atomic orbital) method,[33] using the PCM as described above. Root mean squared error (RMSE) was calculated using each distinct proton signal; calculated chemical shifts were referenced using tetramethylsilane (TMS) as 0 ppm, and shifts from chemically equivalent protons were averaged. From these optimized structures, $S_0 \rightarrow S_n$ transitions were calculated as described in **S5.1 General procedures**, using the PCM as described above, but with parameters for dichloromethane to match the solvent used experimentally. RMSE was calculated using the $S_0 \rightarrow S_1$ and $S_0 \rightarrow S_2$ transitions for each structure. These data are given in **Figure S16** and **Figure S17**.

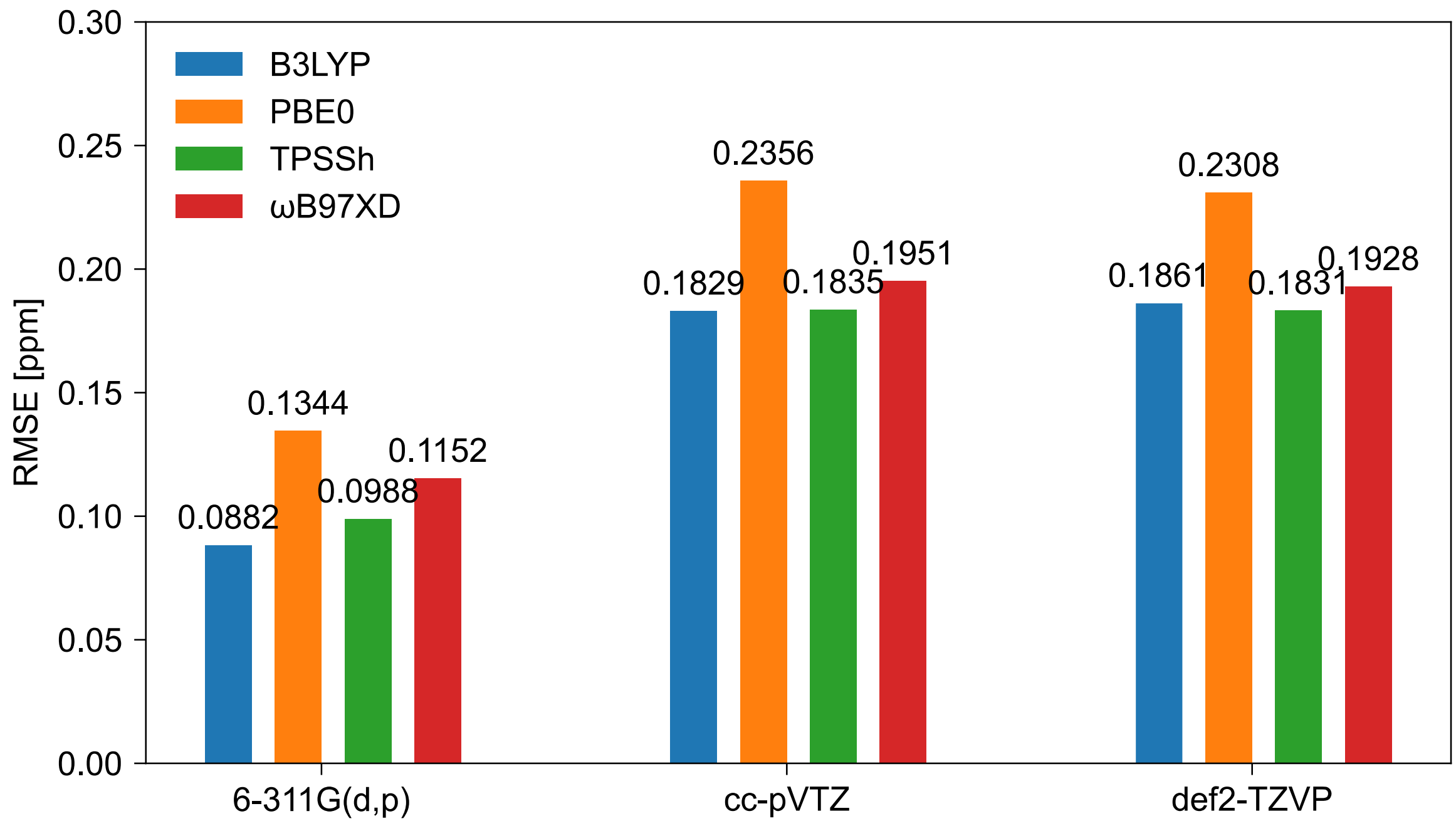

Figure S16 Root mean squared error (RMSE) of computed NMR shifts of **4a**, **4c**, **4d**, and **4f** using various functionals and basis sets.

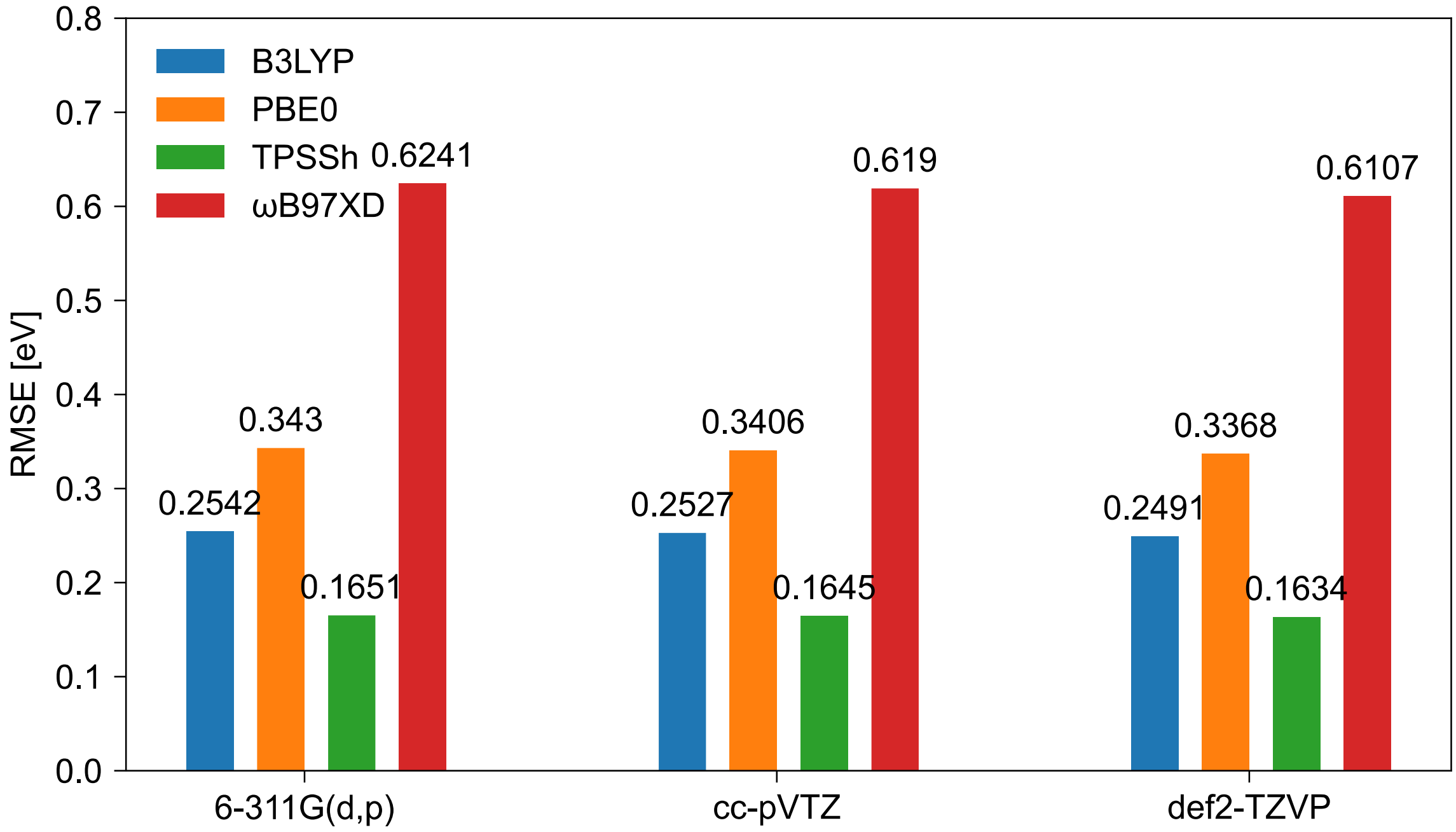

Figure S17 Root mean squared error (RMSE) of computed UV-visible transitions of **4a**, **4c**, **4d**, and **4f** using various functionals and basis sets.

From the above data, B3LYP / 6-311G(d,p) was selected as the functional and basis set combination for the remaining computational work. While TPSSh performed better on the UV-visible transition benchmark, B3LYP performed better on magnetic shielding calculations, which we assessed to be important to the assertions made by this work regarding the aromaticity assignments of these structures.

S5.3 $S_0 \rightarrow S_1$ and T_1 transition energy results

$S_0 \rightarrow S_1$ transition energies were calculated directly using time-dependent density functional theory (TD-DFT, $E(S_1)_v$) and benchmarked against recorded UV-visible absorption spectra. TD-DFT can be an unreliable method for the computation of $S_0 \rightarrow T_1$ transition energies,[34] so to establish a rough estimate of these energies both the vertical excitation and adiabatic excitation methods were used, consistent with literature approaches,[35,36] though TD-DFT results are also shown for reference. We also used the linear correction method of Tayebjee and co-workers, given here as $E(T_1)_c$, which gives accurate values of $E(T_1)$ for similar molecules.[37] **4a** and **4f** were re-optimized as described in **S5.1** but using the B3LYP / 3-21G level of theory, and a linear correction was applied to the adiabatic triplet energy as described in **Equation S1**:

$$E(T_1)_c = \frac{E(T_1)_a + 0.04}{1.12} \tag{S1}$$

The calculated singlet and triplet transition energies are then directly compared with each other, and considered useful for SF if $E(S_1) \geq 2E(T_1)$, and $E(T_1) > 1.1$ eV (the minimum photon energy necessary to produce electricity in a silicon photovoltaic cell).[38] These data are given in **Figure S18**; a full list of calculated energies is given in **Table S19**.

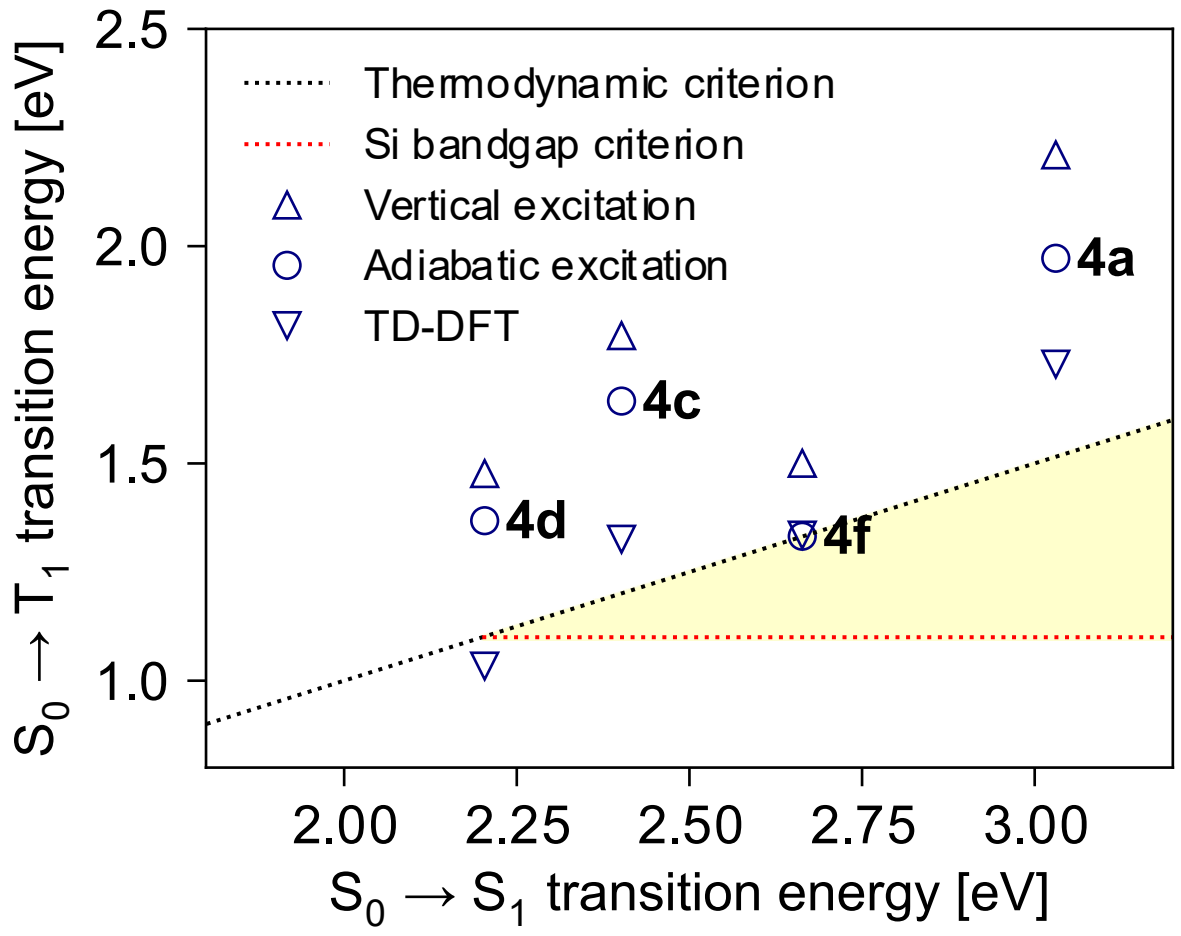

Figure S18 Computed singlet (TD-DFT) and triplet (vertical excitation, adiabatic excitation, TD-DFT) energies of **4a**, **4c**, **4d** and **4f**. The thermodynamic criterion for SF, $E(S_1) \geq 2E(T_1)$, and the bandgap of silicon are given as dotted black and red lines respectively, with the useful area marked in yellow.

At this level of theory, $E(S_1)/E(T_1)$ is still insufficient for exothermic SF in **4c** and **4d**. However, when the carbonyl group is substituted for dicyanomethylene, as in **4f**, $E(S_1)/E(T_1)$ is significantly improved, though we did not observe SF. We also attempted to optimize the geometries of the S_1 states of **4a**, **4c**, **4d**, and **4f** using TD-DFT. However, the resulting adiabatic S_1 energies ($E(S_1)_a$) indicate a poor description of the excited state: the $E(S_1)_a$ of **4a**, **4c** and **4d** are 0.65, 0.53, and 0.50 eV respectively. In **4c** and **4d**, since $E(S_1)_a < E(T_1)_a$, the calculated $E(S_1)_a$ results were inconsistent with the experimentally-observed ISC. We were not able to obtain an optimized S_1 structure for **4f** due to convergence difficulties, though the lowest energy obtained in the optimization (1.21 eV) is lower than the calculated $E(T_1)_a$ and so would also preclude ISC in that compound.

Table S19 Calculated TD-B3LYP/6-311G(d,p) singlet and triplet excitations of **4a**, **4c**, **4d**, and **4f**. All energies are given in units of eV.[a] Structures with singlet and triplet energies sufficient for exo- or isothermic singlet fission are marked in green, others are marked in red.

Mole-cule	$E(S_1)_v$	$E(T_1)_v$	$E(T_1)_a$	$E(T_1)_c$	$E(T_1)_t$	$E(T_2)_t$ [b]	$E(S_1)$ / $E(T_1)_a$	$E(S_1)$ / $E(T_1)_c$	$E(T_2)_t$ / $E(T_1)_a$
**4a**	3.03	2.21	1.97	1.49	1.73	0.69	**1.54**	**2.03**	0.35
**4c**	2.40	1.79	1.64	*n.d.*	1.33	0.91	**1.46**	*n.d.*	0.55
**4d**	2.20	1.48	1.37	*n.d.*	1.03	0.84	**1.61**	*n.d.*	0.61
**4f**	2.66	1.50	1.33	1.15	1.34	1.04	**2.00**	**2.32**	0.78

[a] $E(T_n)_v$, $E(T_n)_a$, $E(T_1)_c$ and $E(T_n)_t$ are the vertical, adiabatic, corrected, and TD-DFT-derived triplet energies of *n*th state respectively. [b] Calculated from the T_1 structure.

S5.4 Natural bond orbital (NBO) analysis

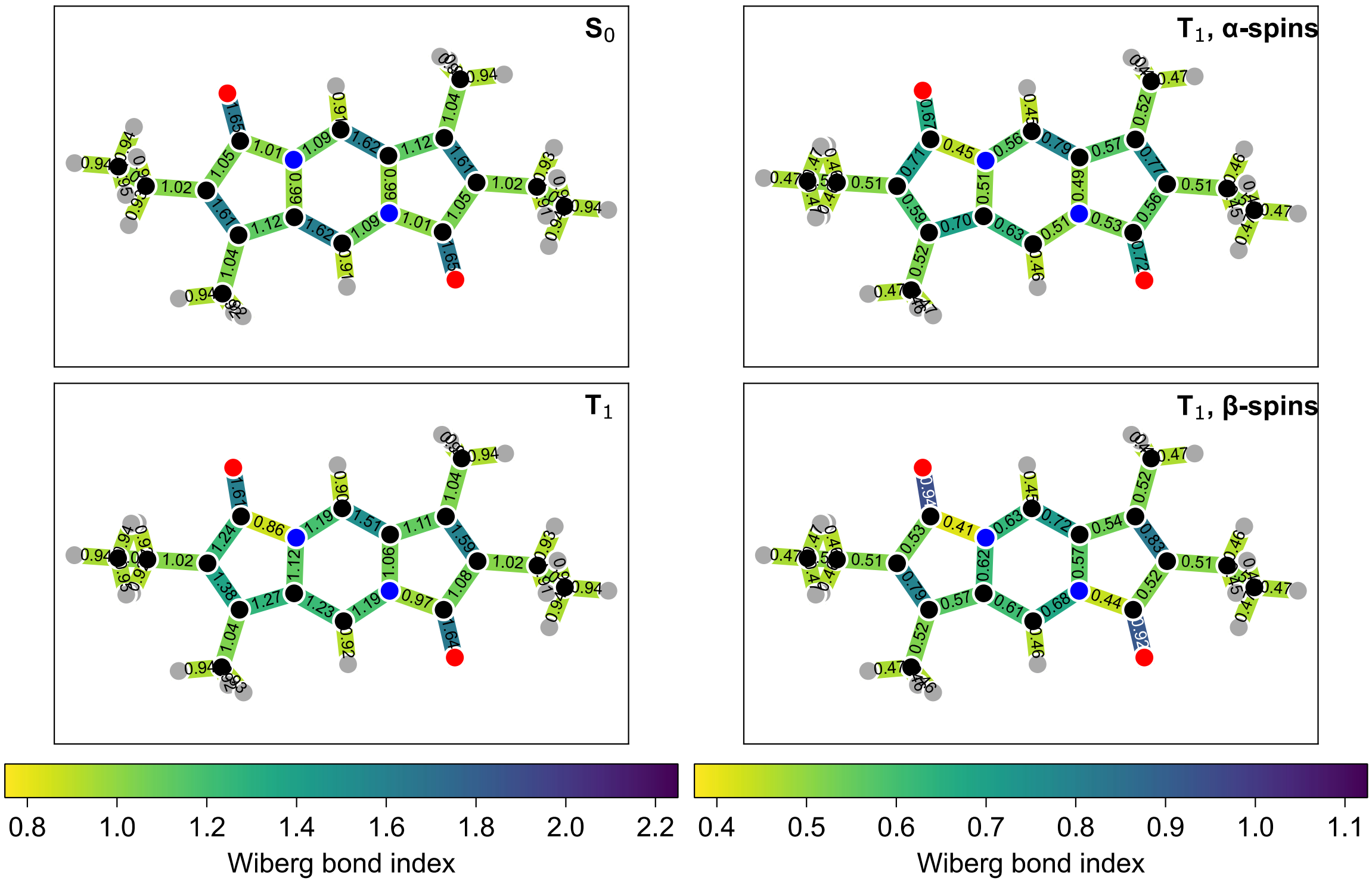

Figure S19 Calculated Wiberg bond indices of **4a** in the singlet and triplet states (B3LYP / 6-311G(d,p)).

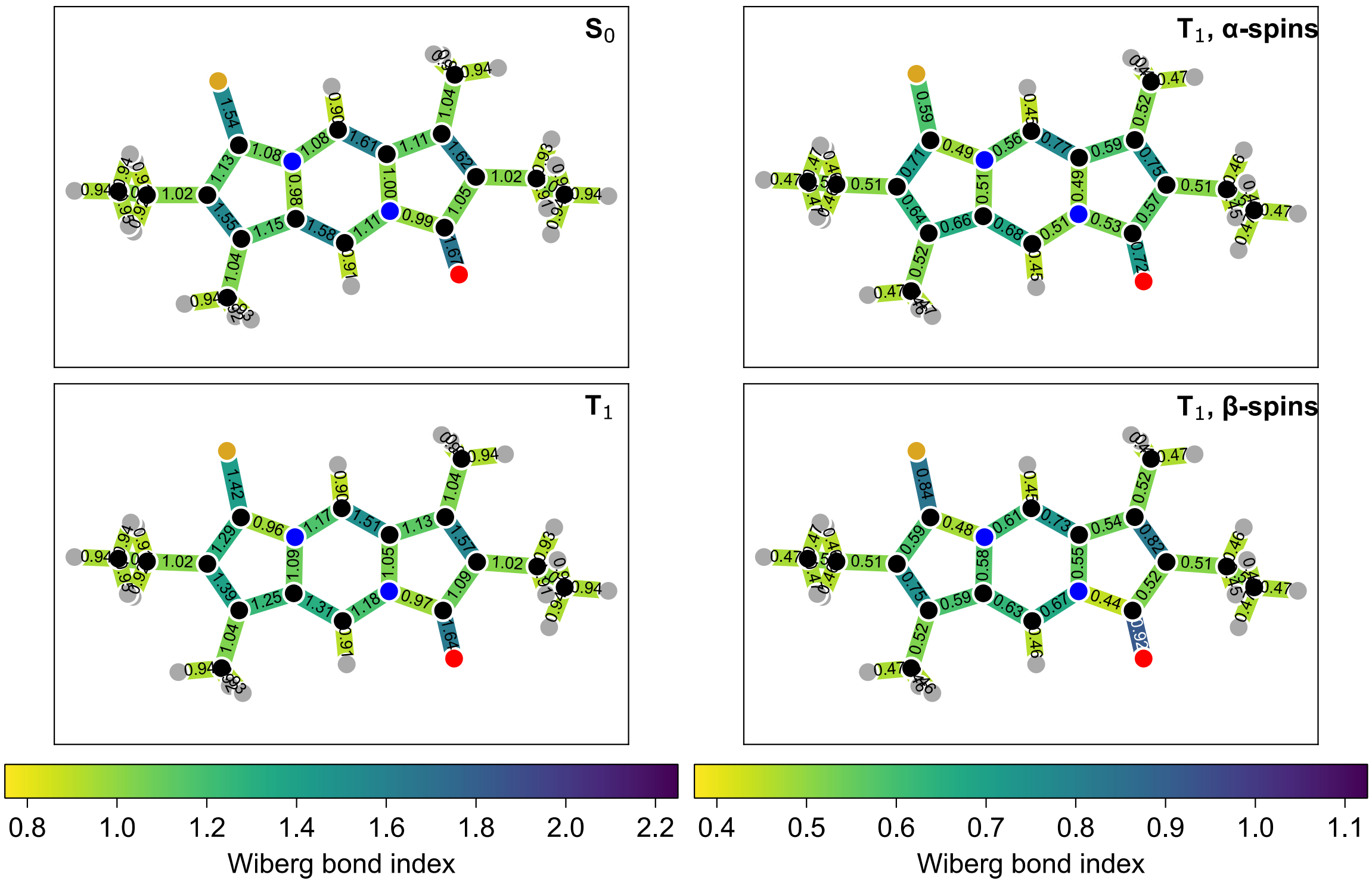

Figure S20 Calculated Wiberg bond indices of **4c** in the singlet and triplet states (B3LYP / 6-311G(d,p)).

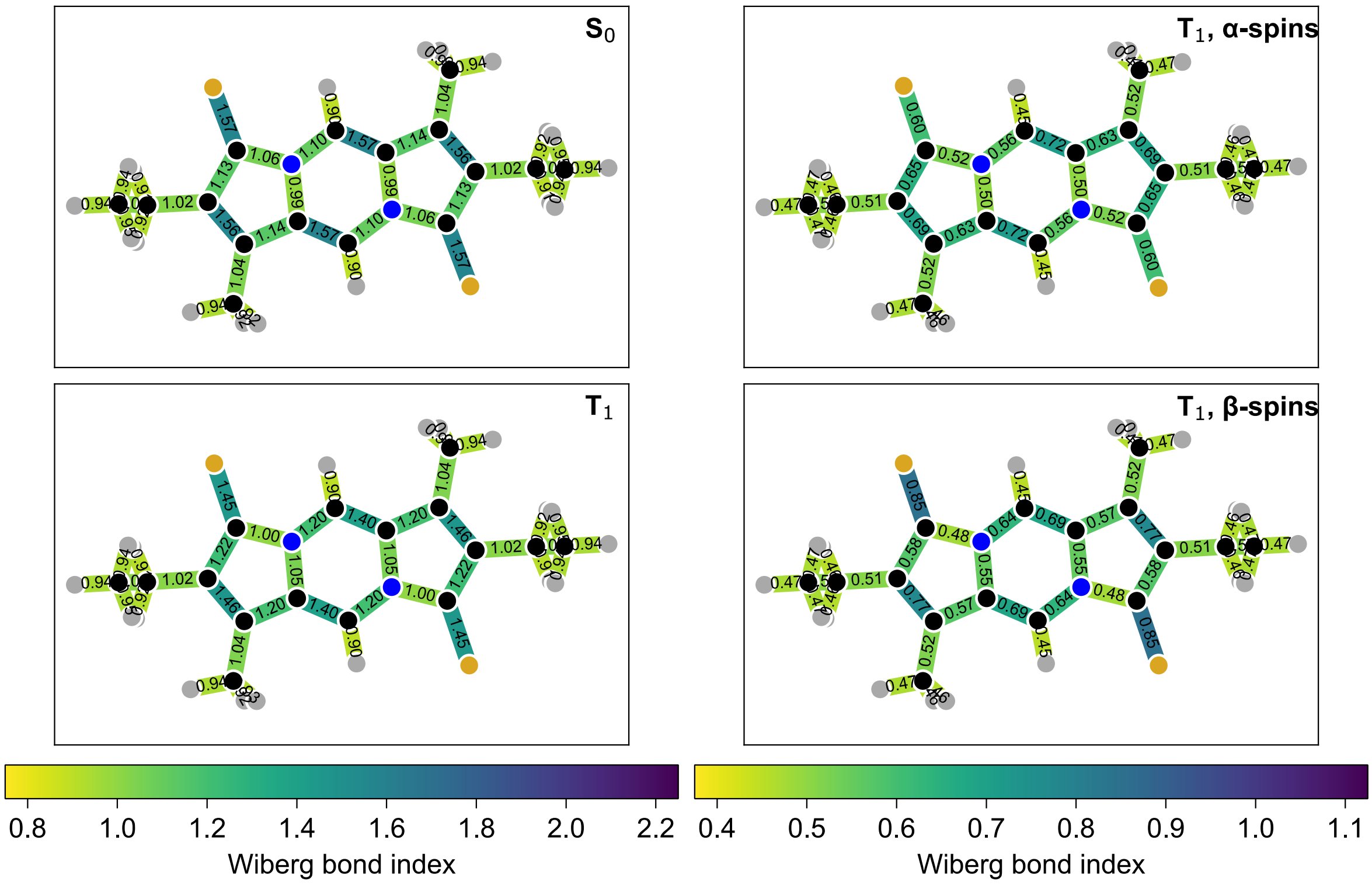

Figure S21 Calculated Wiberg bond indices of **4d** in the singlet and triplet states (B3LYP / 6-311G(d,p)).

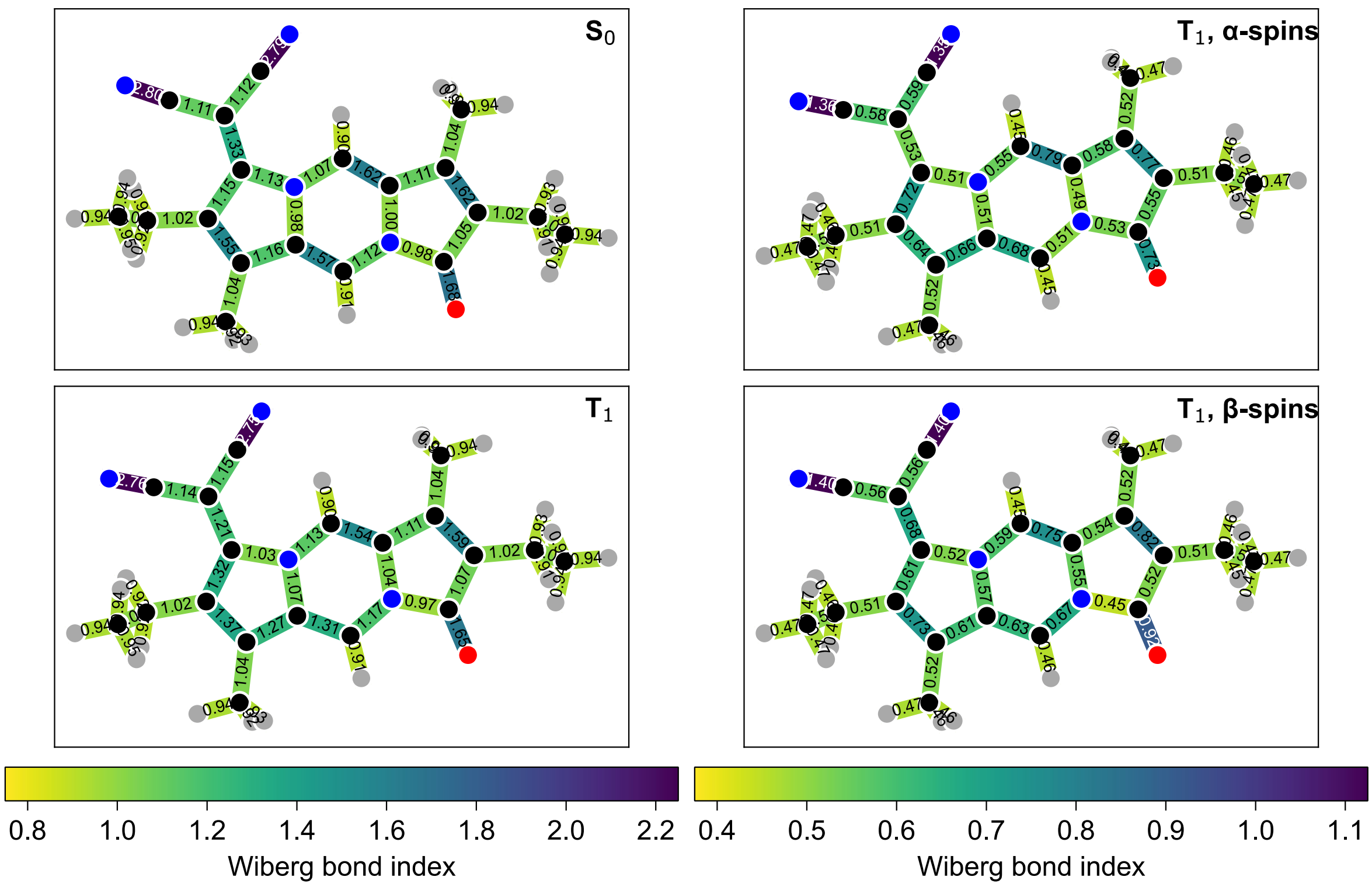

Figure S22 Calculated Wiberg bond indices of **4f** in the singlet and triplet states (B3LYP / 6-311G(d,p)).

S5.5 Calculated bond lengths

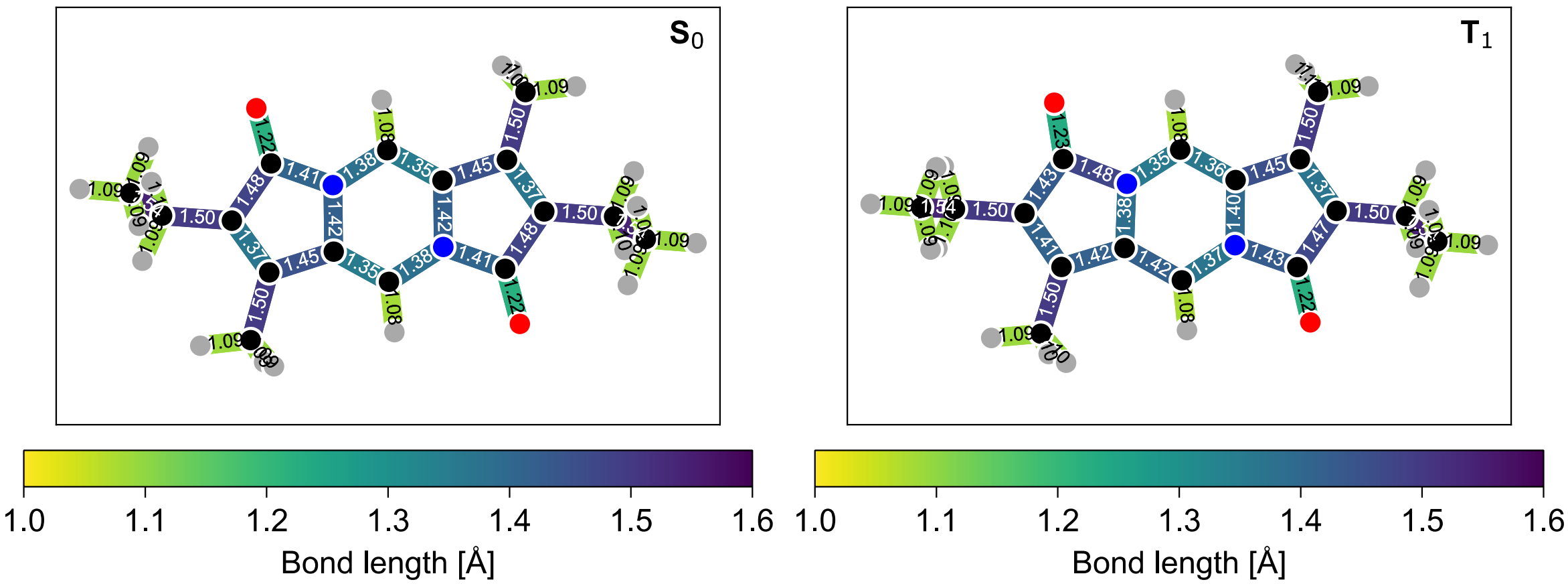

Figure S23 Calculated bond lengths of **4a** in the singlet and triplet states (B3LYP / 6-311G(d,p)).

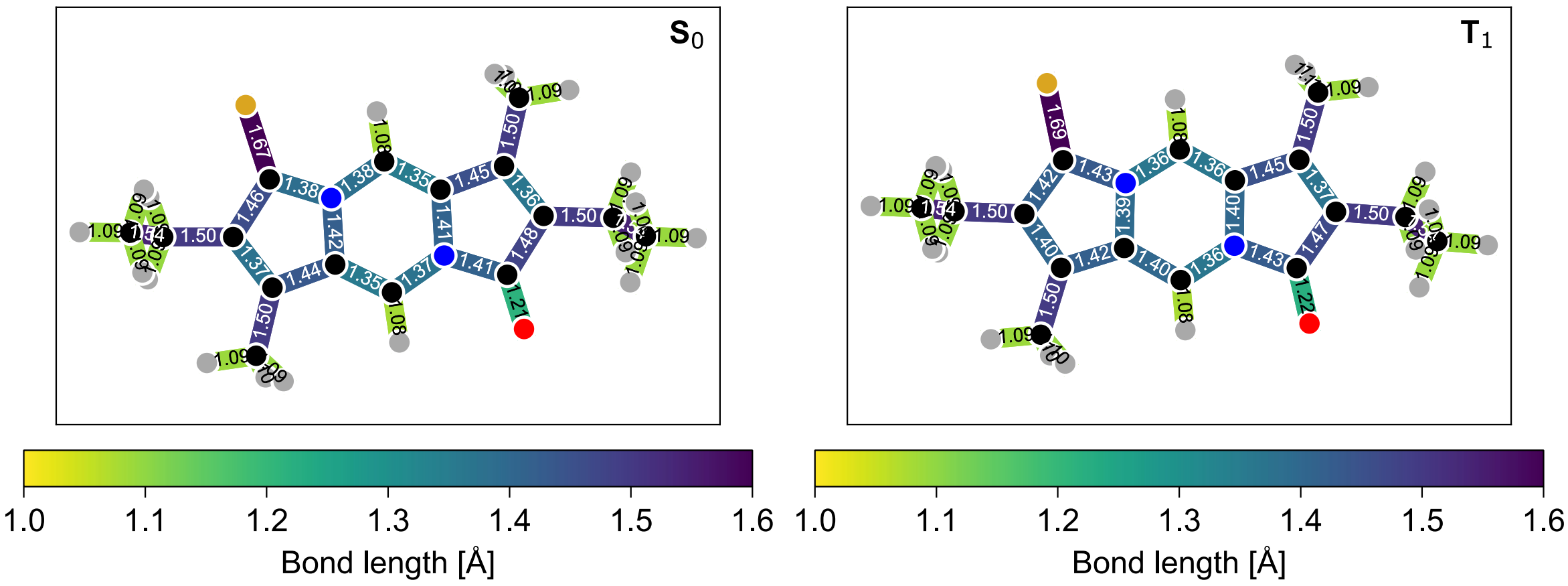

Figure S24 Calculated bond lengths of **4c** in the singlet and triplet states (B3LYP / 6-311G(d,p)).

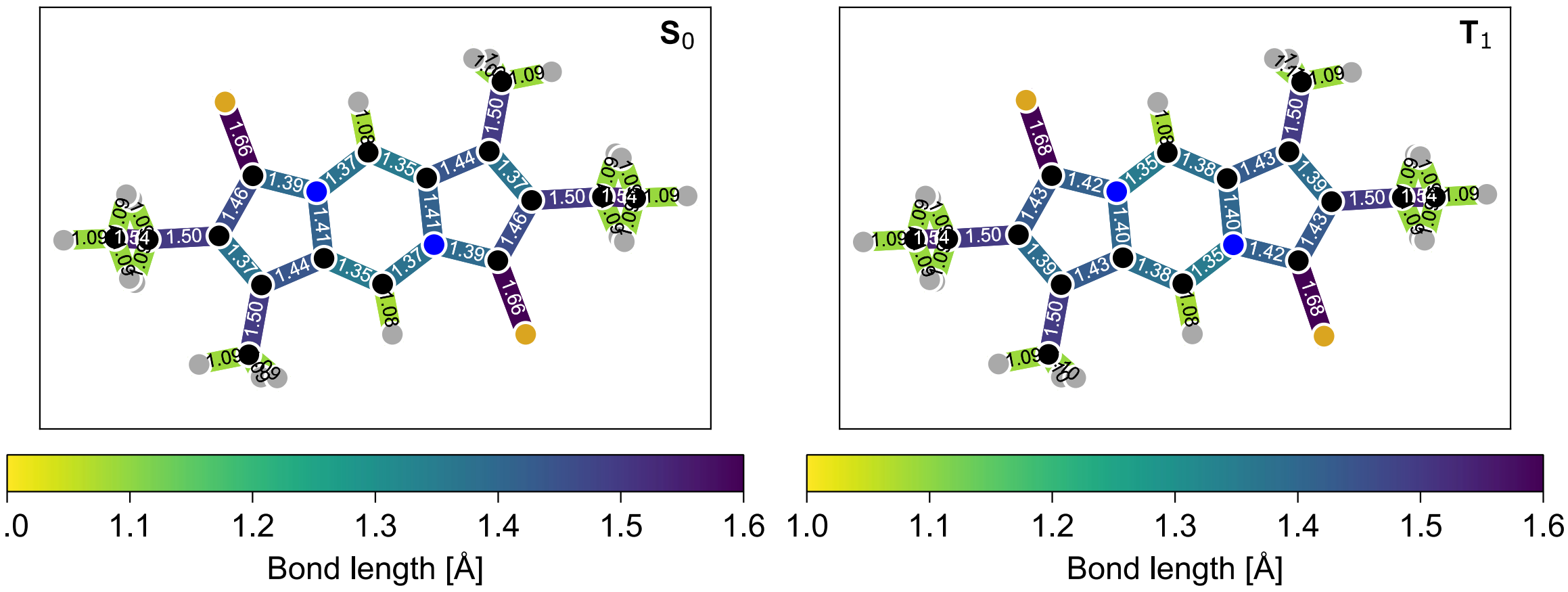

Figure S25 Calculated bond lengths of **4d** in the singlet and triplet states (B3LYP / 6-311G(d,p)).

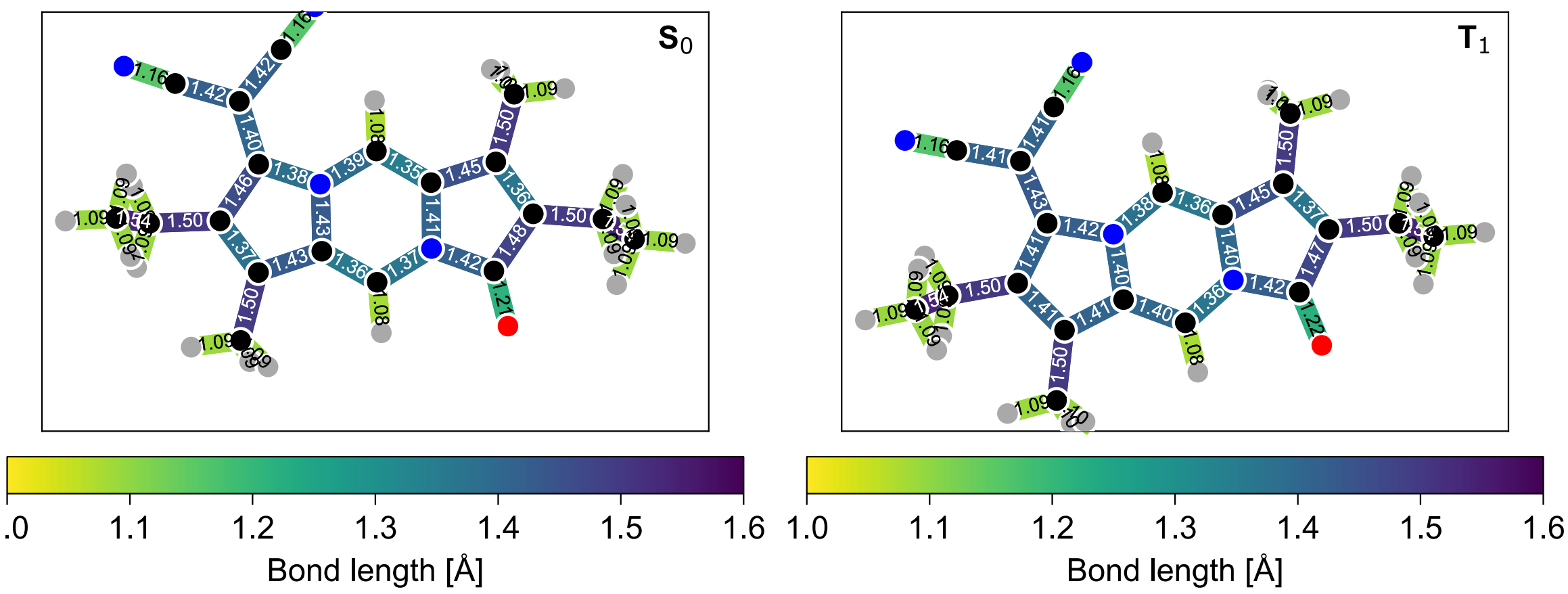

Figure S26 Calculated bond lengths of **4f** in the singlet and triplet states (B3LYP / 6-311G(d,p)).

S5.6 Spin density calculations

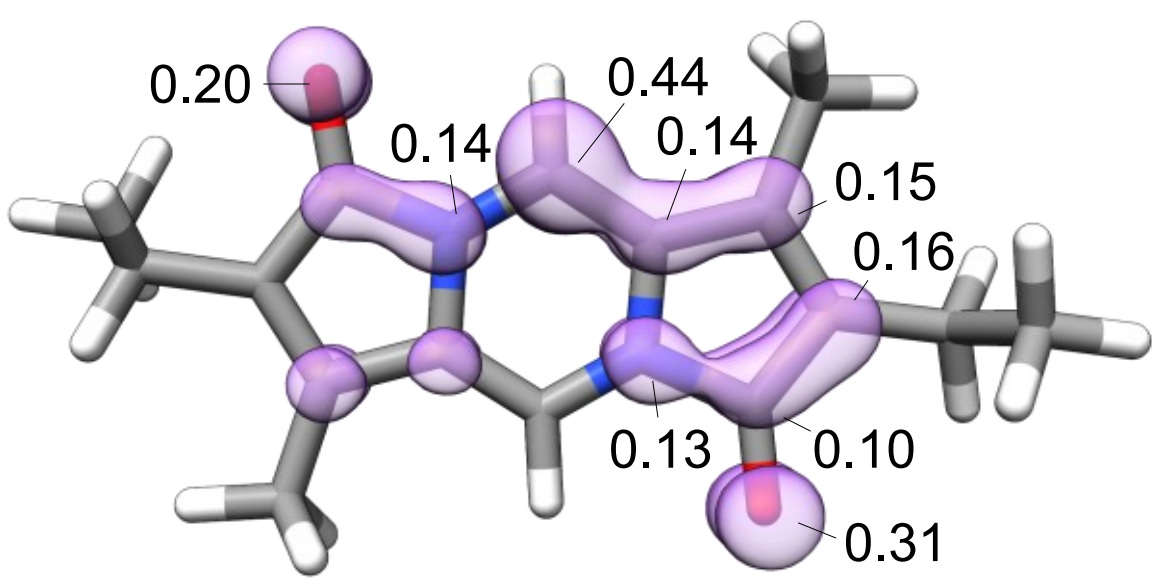

Figure S27 Calculated spin density isosurface of **4a** in the triplet state (B3LYP / 6-311G(d,p)). Isovalue = 0.005. Natural spin density values given where NSD ≥ 0.1.

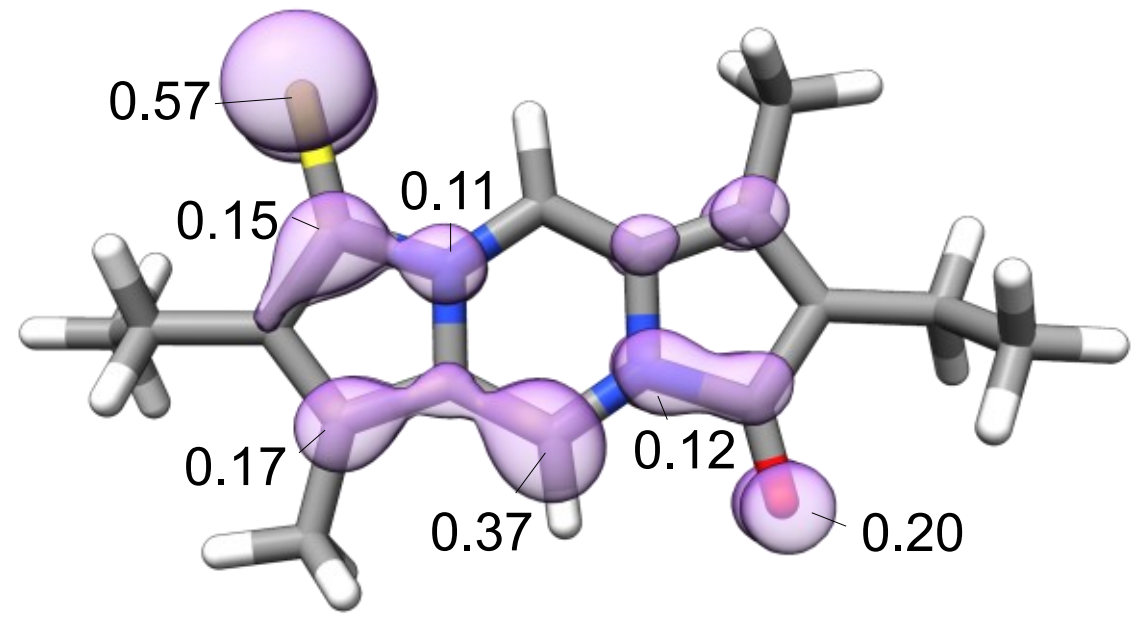

Figure S28 Calculated spin density isosurface of **4c** in the triplet state (B3LYP / 6-311G(d,p)). Isovalue = 0.005. Natural spin density values given where NSD ≥ 0.1.

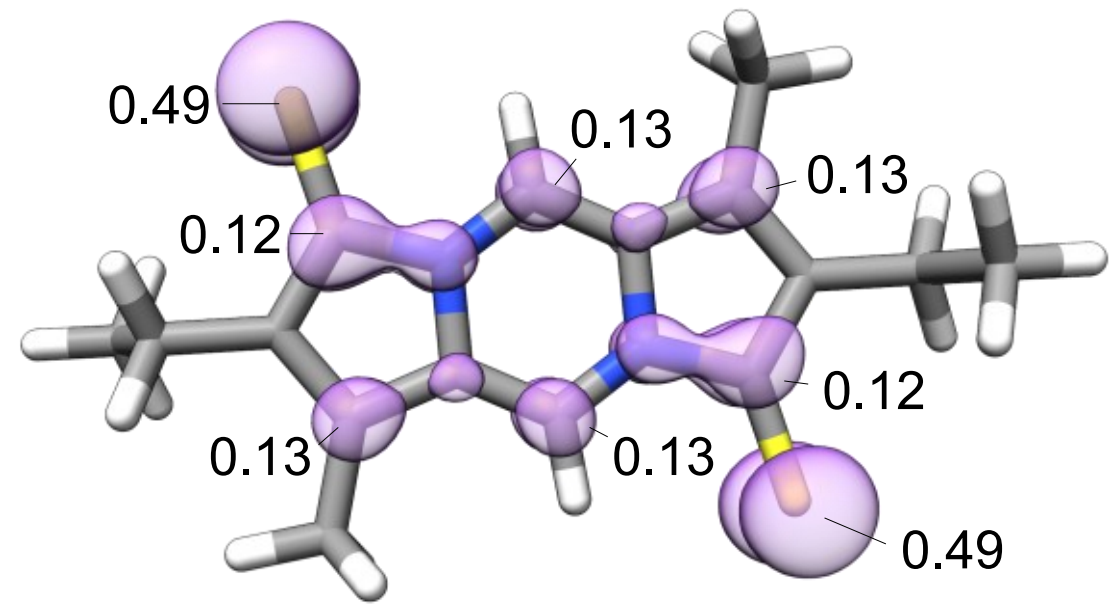

Figure S29 Calculated spin density isosurface of **4d** in the triplet state (B3LYP / 6-311G(d,p)). Isovalue = 0.005. Natural spin density values given where NSD ≥ 0.1.

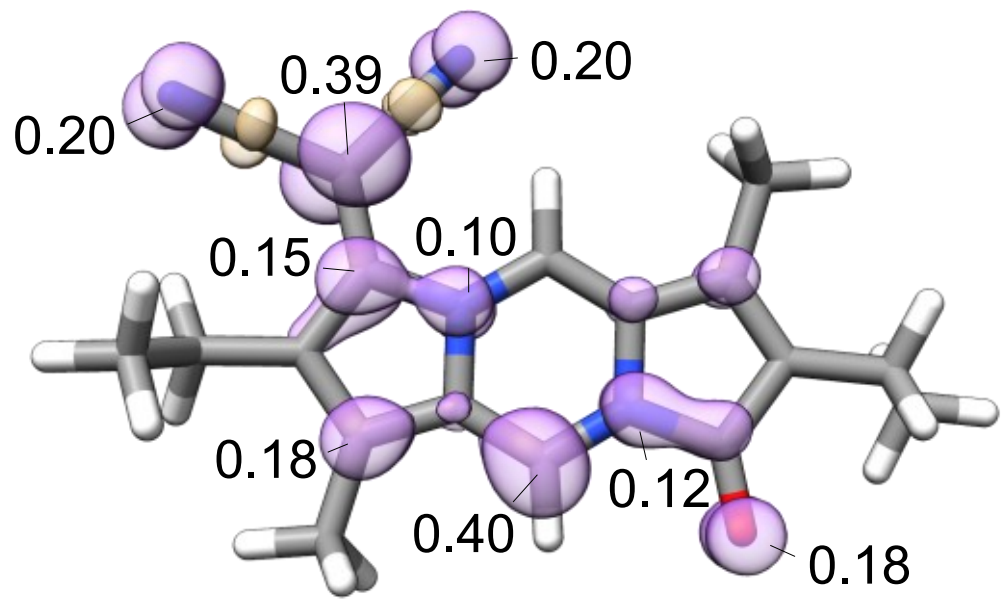

Figure S30 Calculated spin density isosurface of **4f** in the triplet state (B3LYP / 6-311G(d,p)). Isovalue = 0.005. Natural spin density values given where NSD ≥ 0.1.

S5.7 Electron density of delocalized bonds (EDDB) calculations

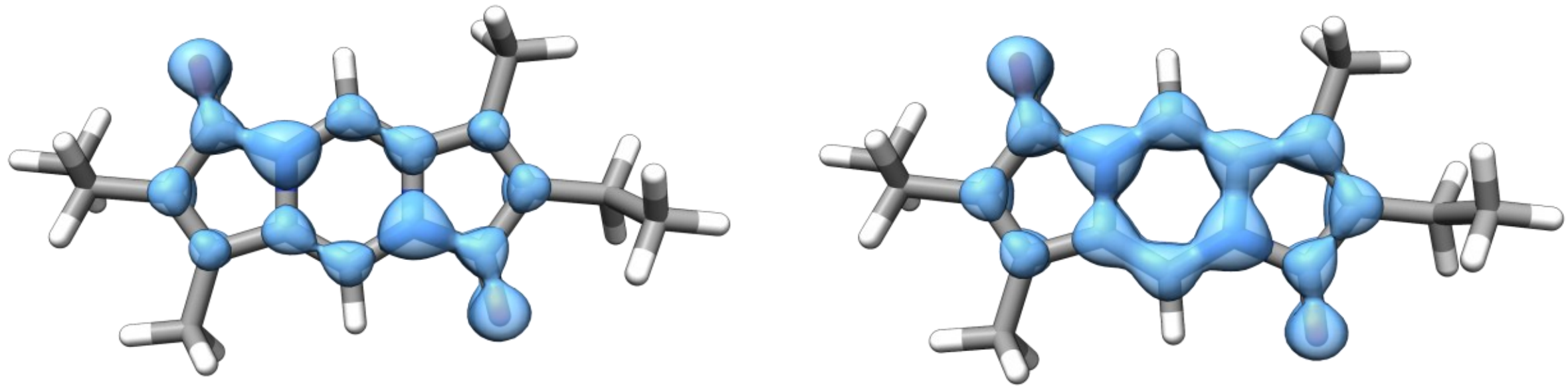

Figure S31 Calculated $EDDB_G$ isosurface of **4a** singlet (*left*) and triplet (*right*) (B3LYP / 6-311G(d,p)). Isovalue = 0.025.

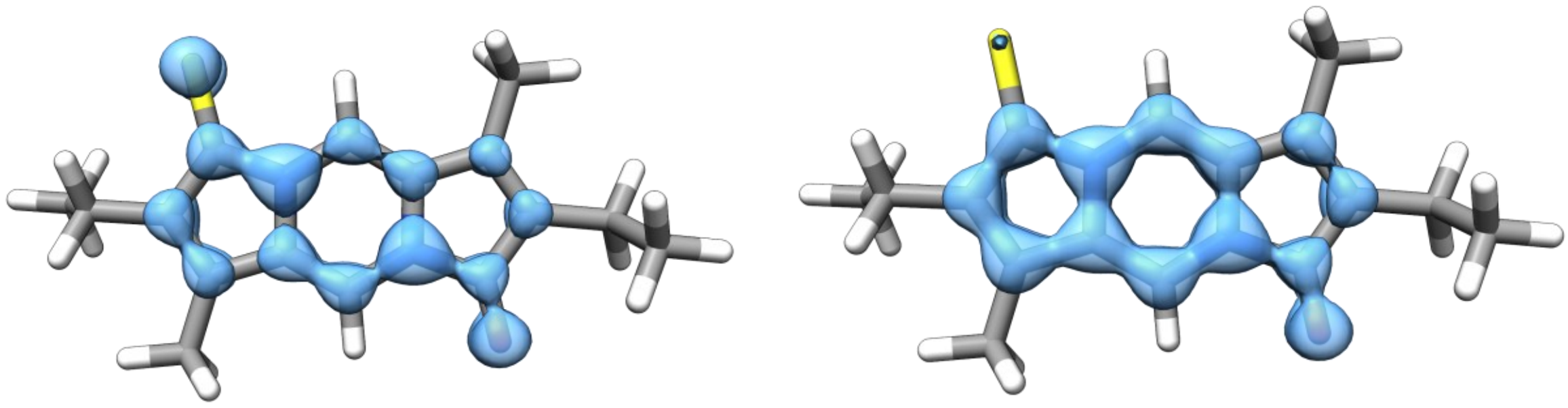

Figure S32 Calculated $EDDB_G$ isosurface of **4c** singlet (*left*) and triplet (*right*) (B3LYP / 6-311G(d,p)). Isovalue = 0.025.

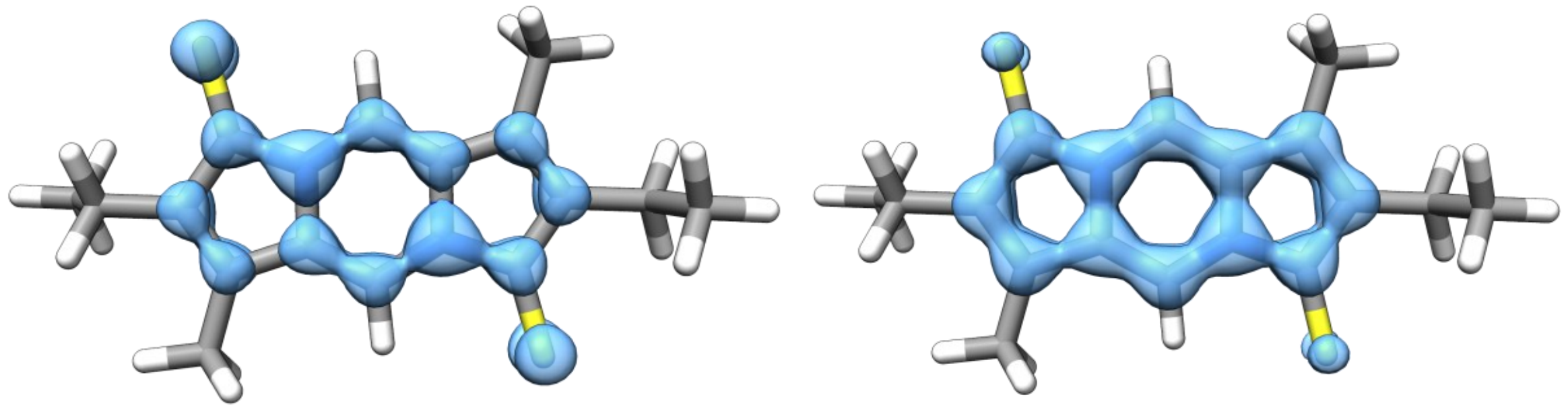

Figure S33 Calculated $EDDB_G$ isosurface of **4d** singlet (*left*) and triplet (*right*) (B3LYP / 6-311G(d,p)). Isovalue = 0.025.

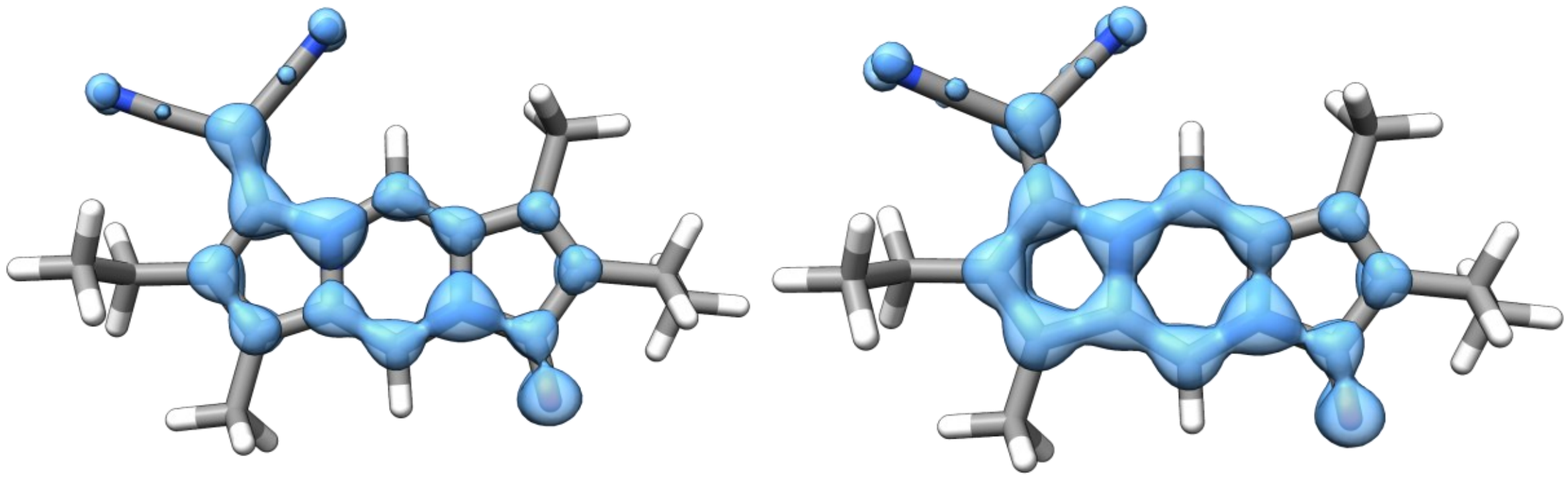

Figure S34 Calculated $EDDB_G$ isosurface of **4f** singlet (*left*) and triplet (*right*) (B3LYP / 6-311G(d,p)). Isovalue = 0.025.

S5.8 Nucleus-independent chemical shift

The σ-only model was implemented according to a procedure adapted from that reported by Stanger.[39] To create the σ-only models, hydrogen atoms were added at an angle of 95° to the ring plane facing outwards, with a distance of 1.00 Å from each carbon atom in a ring, and 0.96 Å from each heteroatom, including exocyclic oxygen atoms. The additional hydrogen atoms in the σ-only models were not optimized before analytical calculations were performed, which is consistent with the operation of the AROMA package.[40] For both the σ-only models and the original structures, a line of ghost atoms (Bq) was drawn 1.7 Å above the ring plane on the opposite face to the additional hydrogen atoms (for σ-only), in accordance with the NICS-XY method described by Gershoni-Poranne and Stanger.[41] This line connects the centroids of each ring and extends 2.5 Å beyond the centroids of the terminal rings. NMR shielding tensors at each ghost atom were calculated using the gauge-independent atomic orbital (GIAO) technique, a fine CPHF grid, and 6 d functions. Structures in the σ-only model were calculated with a charge of +2; the non-σ-only models were calculated as uncharged. The triplet σ-only models were calculated at the triplet geometry with a multiplicity of 1, as the unpaired electrons are expected to reside within the π-system and are thus extinguished by the σ-only model.

$NICS_{\pi,zz}$ for singlet and triplet structures were calculated by subtracting the zz shielding tensors obtained with the σ-only model from the base model at each ghost atom. These data are given in **Figure S35**.

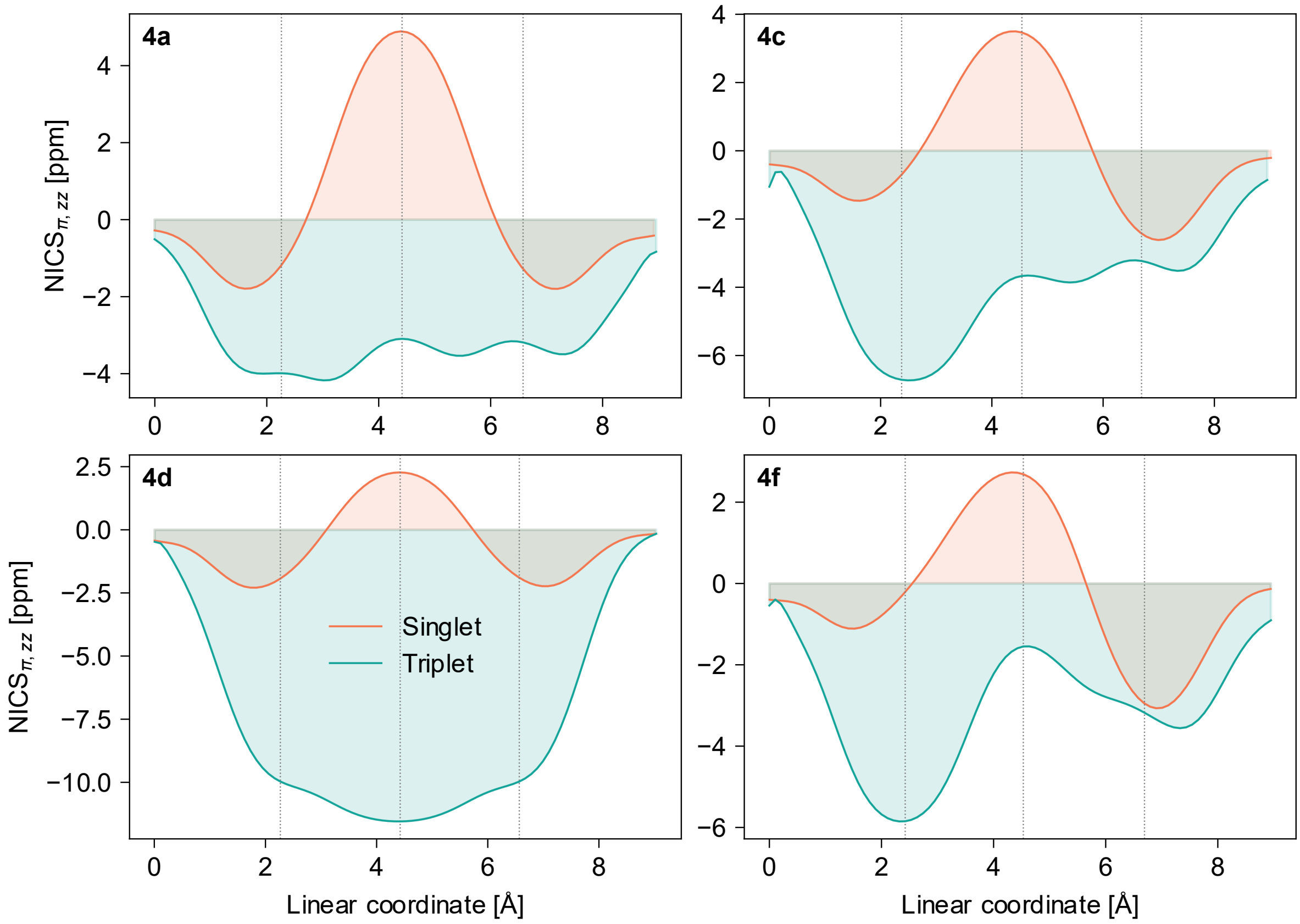

Figure S35 Singlet and triplet NICS$_{\pi,zz}$ plots of **4a**, **4c**, **4d** and **4f**, measured 1.7 Å above the ring plane (B3LYP / 6-311G(d,p)). Ring centroids are shown as dotted lines. Smooth line obtained by interpolation of discrete data points spaced *c.* 0.1 Å apart.

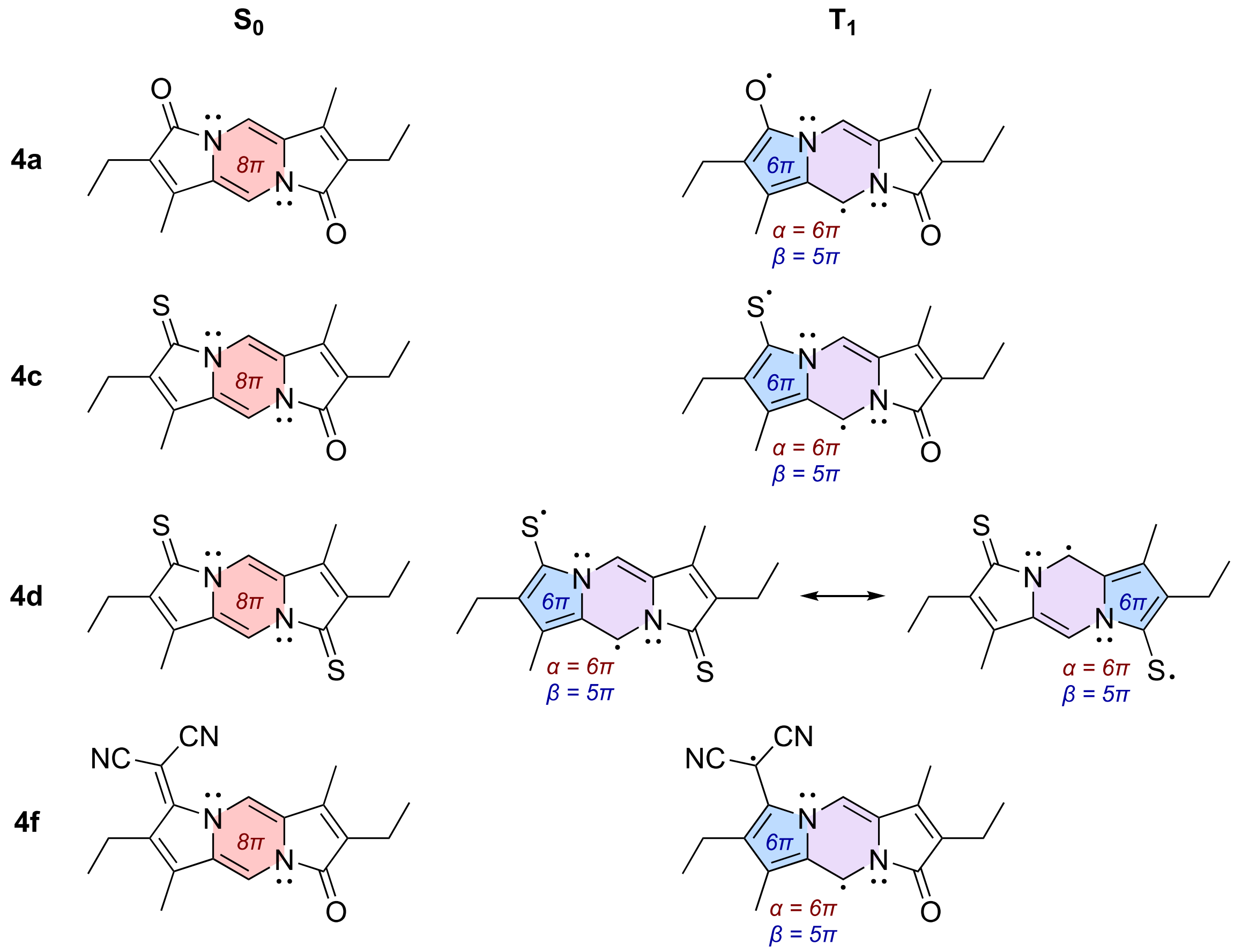

Figure S36 Dominant resonance structures of **4a**, **4c**, **4d** and **4f** in the singlet and triplet states, assigned based on analysis of the above computational metrics (Wiberg bond indices, calculated bond lengths, spin density, EDDB and $NICS_{\pi,zz}$). Mandado (anti)aromatic rings are marked. The resonance-hybrid nature of **4d** in the triplet state is shown as its two individual Lewis structures.

S6. Additional mechanisms

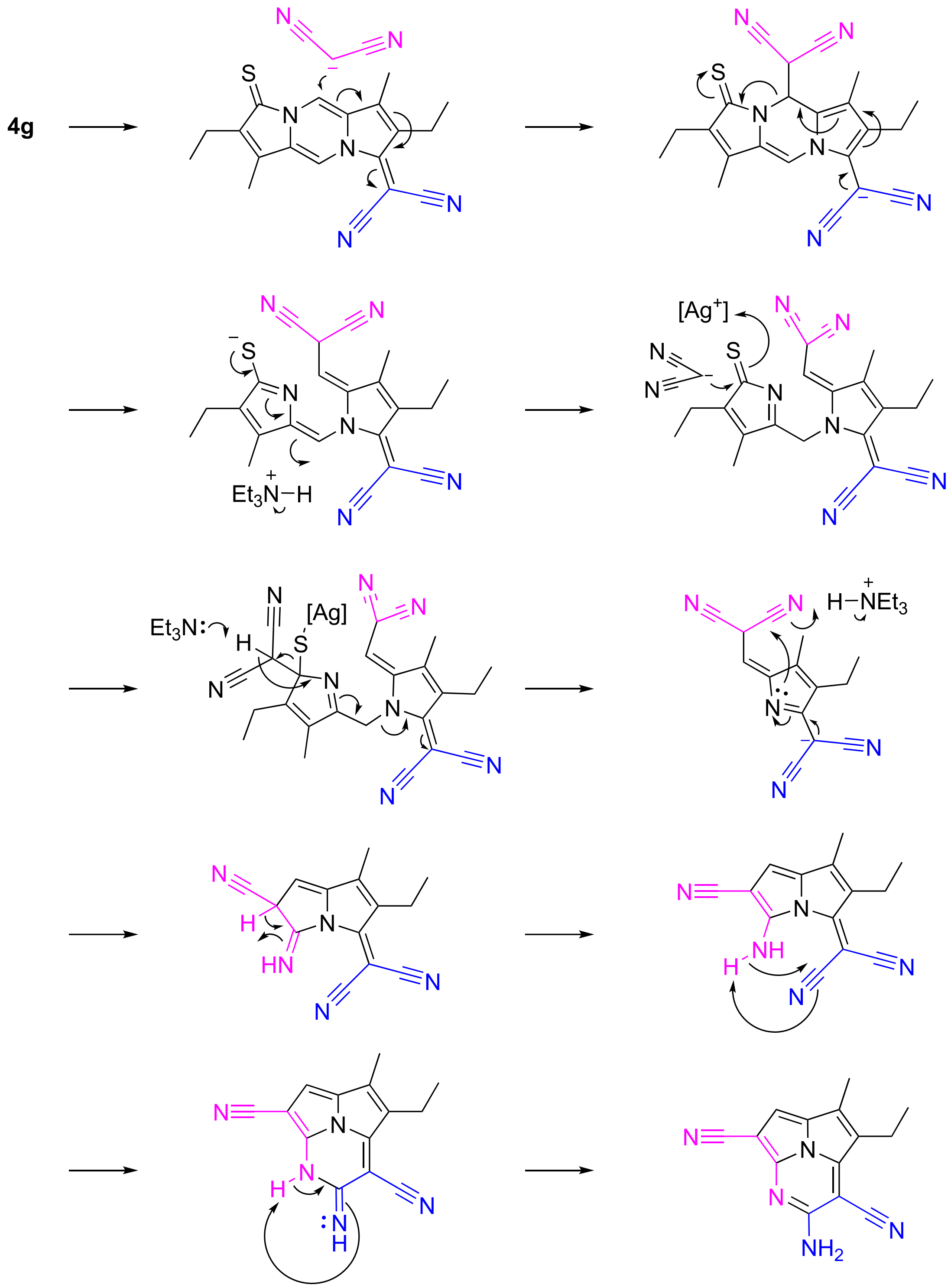

Figure S37 A proposed mechanism for formation of **5**.

S7. NMR spectra

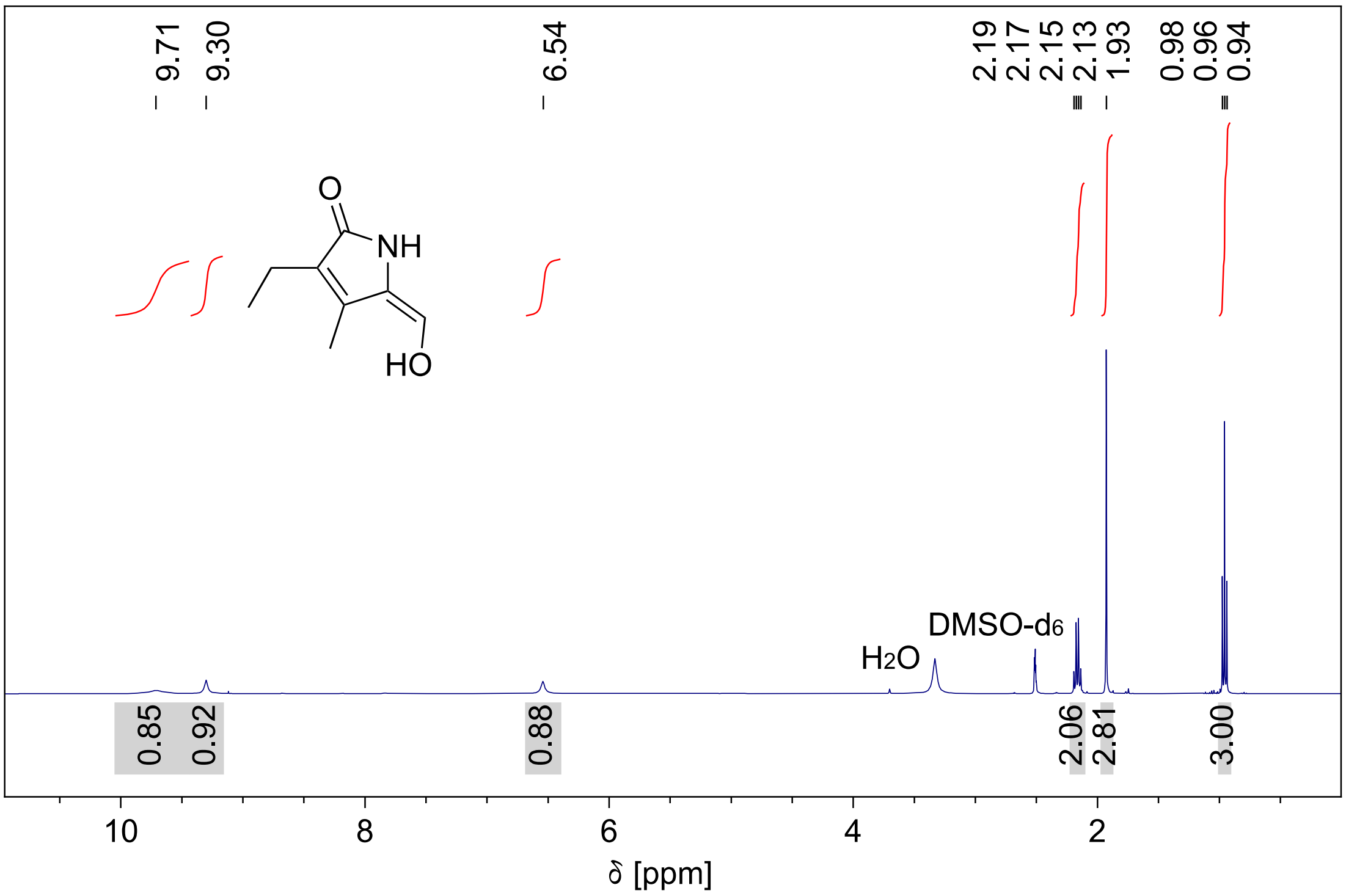

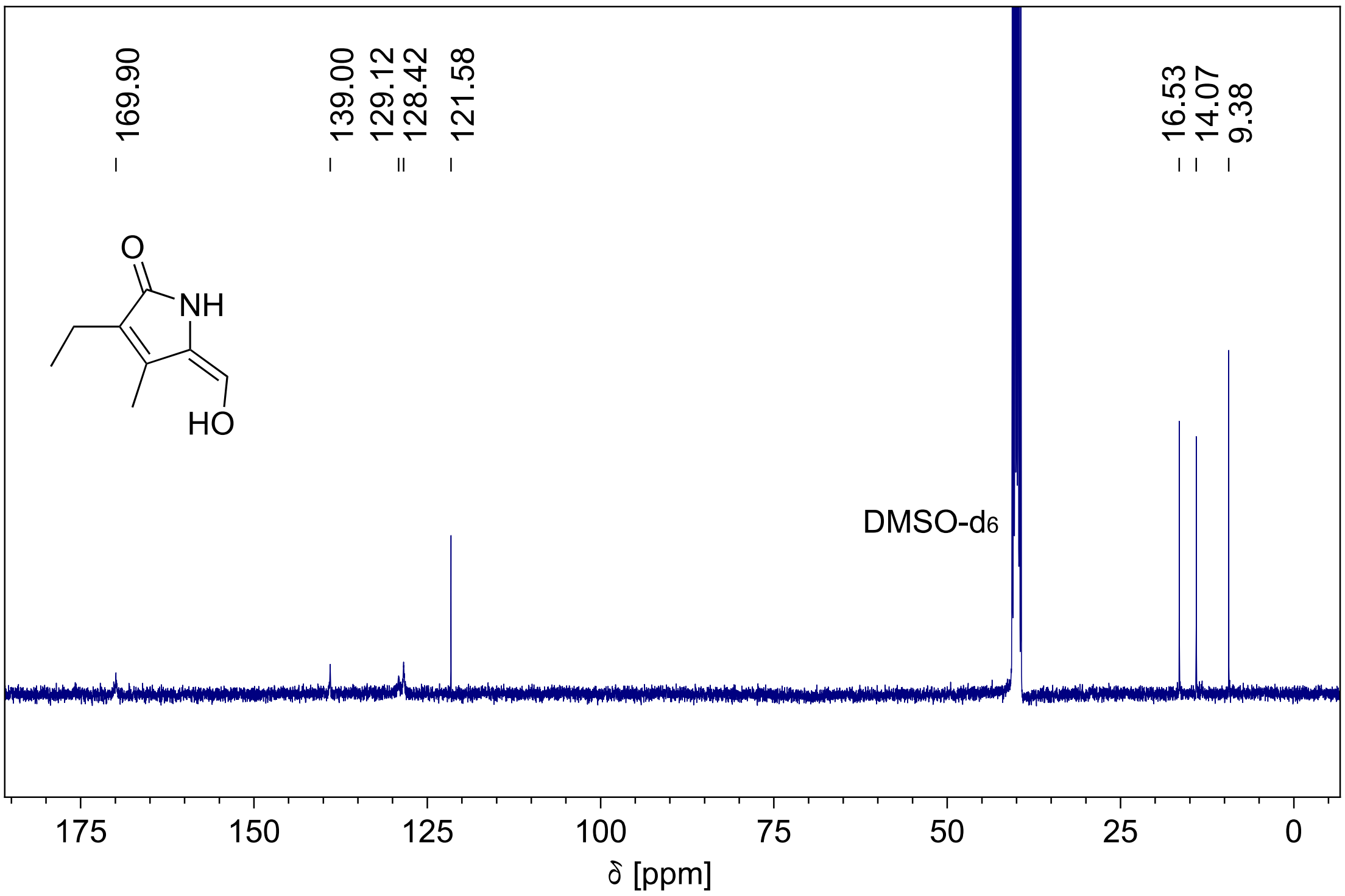

Figure S38 (*Top*) ^{1}H NMR spectrum of enol **2**, DMSO-d$_6$, 400 MHz, 298 K. *(Bottom)* ^{13}C NMR spectrum of enol **2**, DMSO-d$_6$, 101 MHz, 298 K.

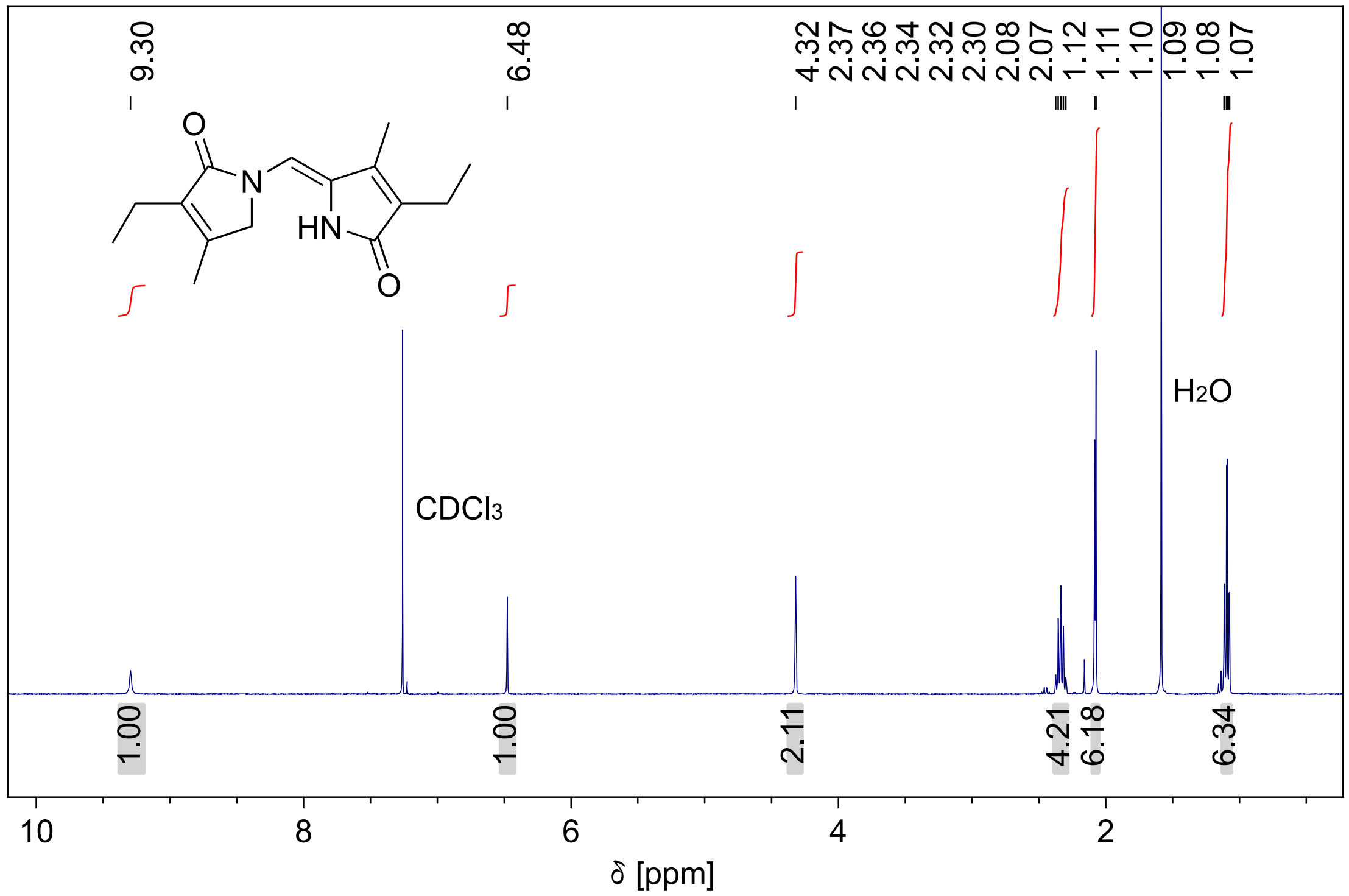

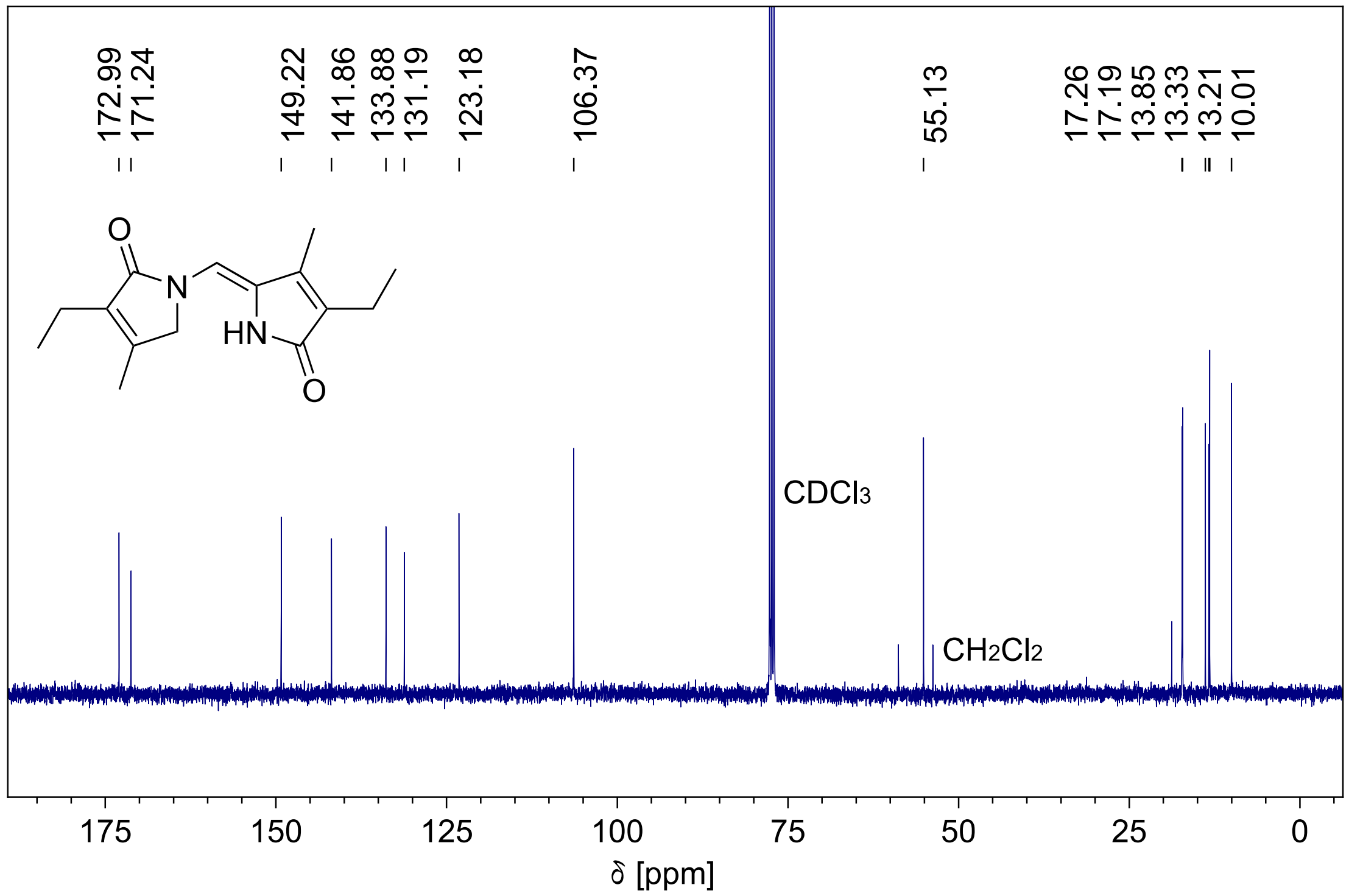

Figure S39 (*Top*) ^{1}H NMR spectrum of open dimer **3**, $CDCl_3$, 400 MHz, 298 K. *(Bottom)* ^{13}C NMR spectrum of open dimer **3**, $CDCl_3$, 101 MHz, 298 K.

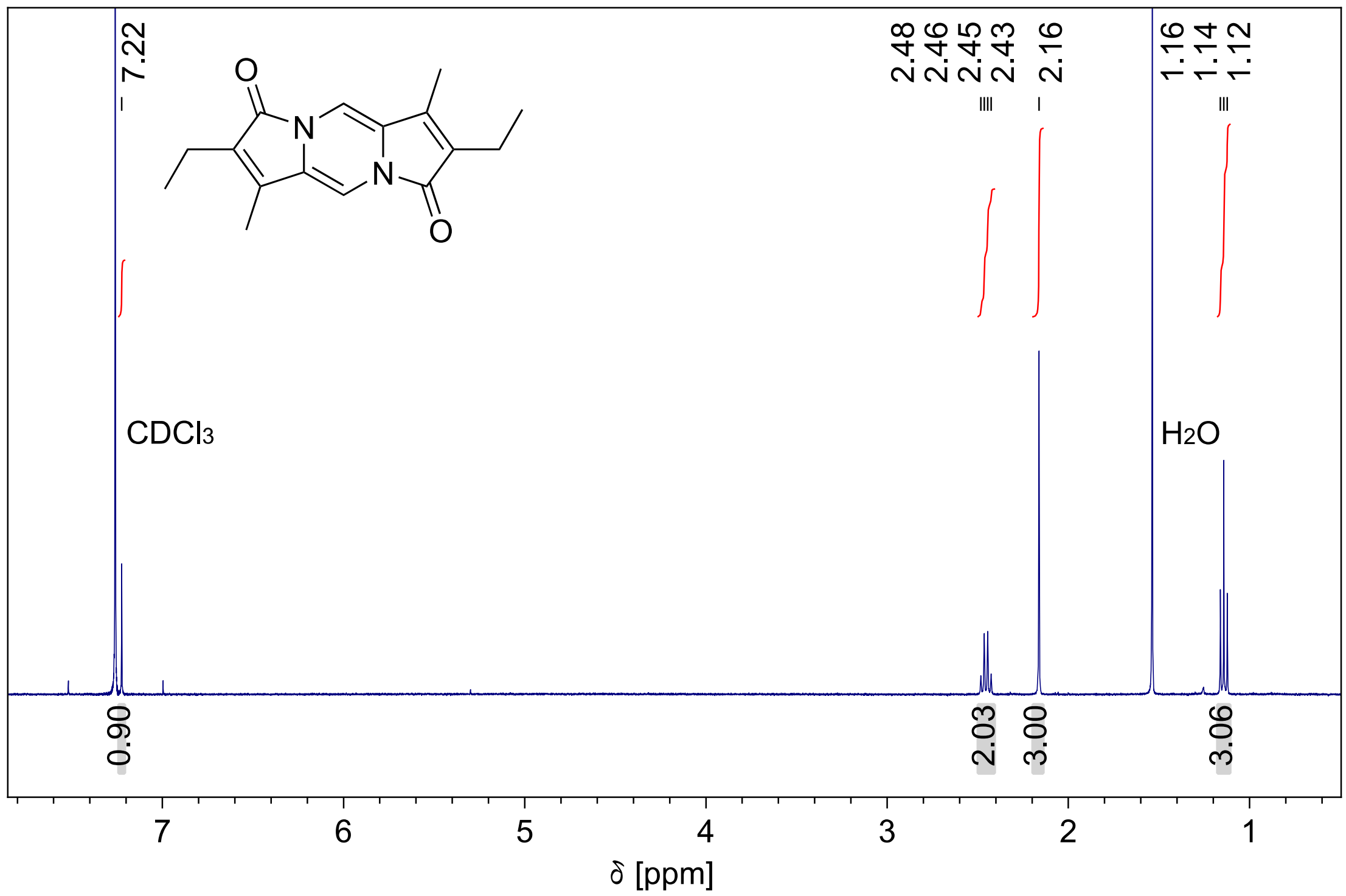

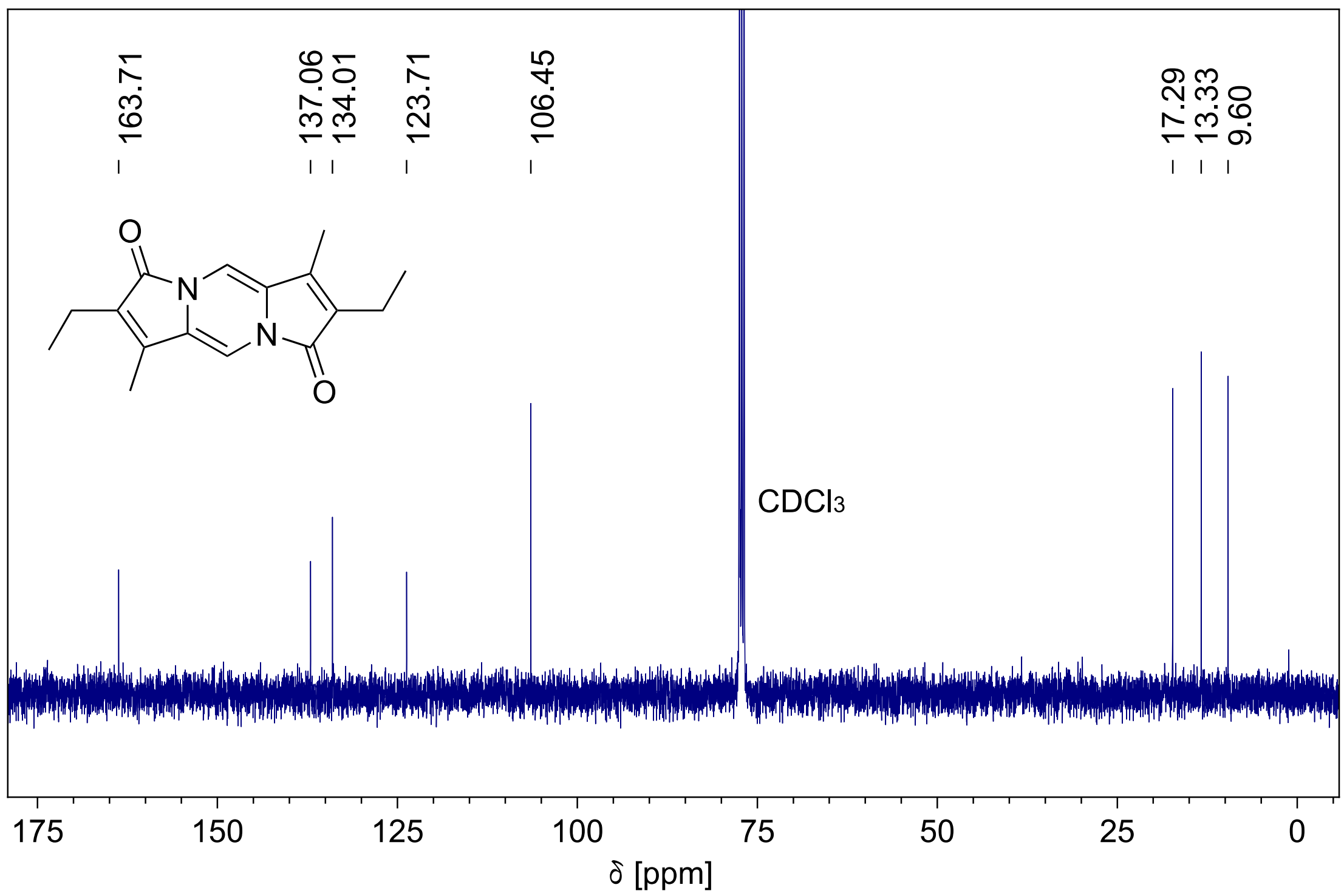

Figure S40 (*Top*) ^{1}H NMR spectrum of isopyrocoll **4a**, $CDCl_3$, 400 MHz, 298 K. *(Bottom)* ^{13}C NMR spectrum of isopyrocoll **4a**, $CDCl_3$, 101 MHz, 298 K.

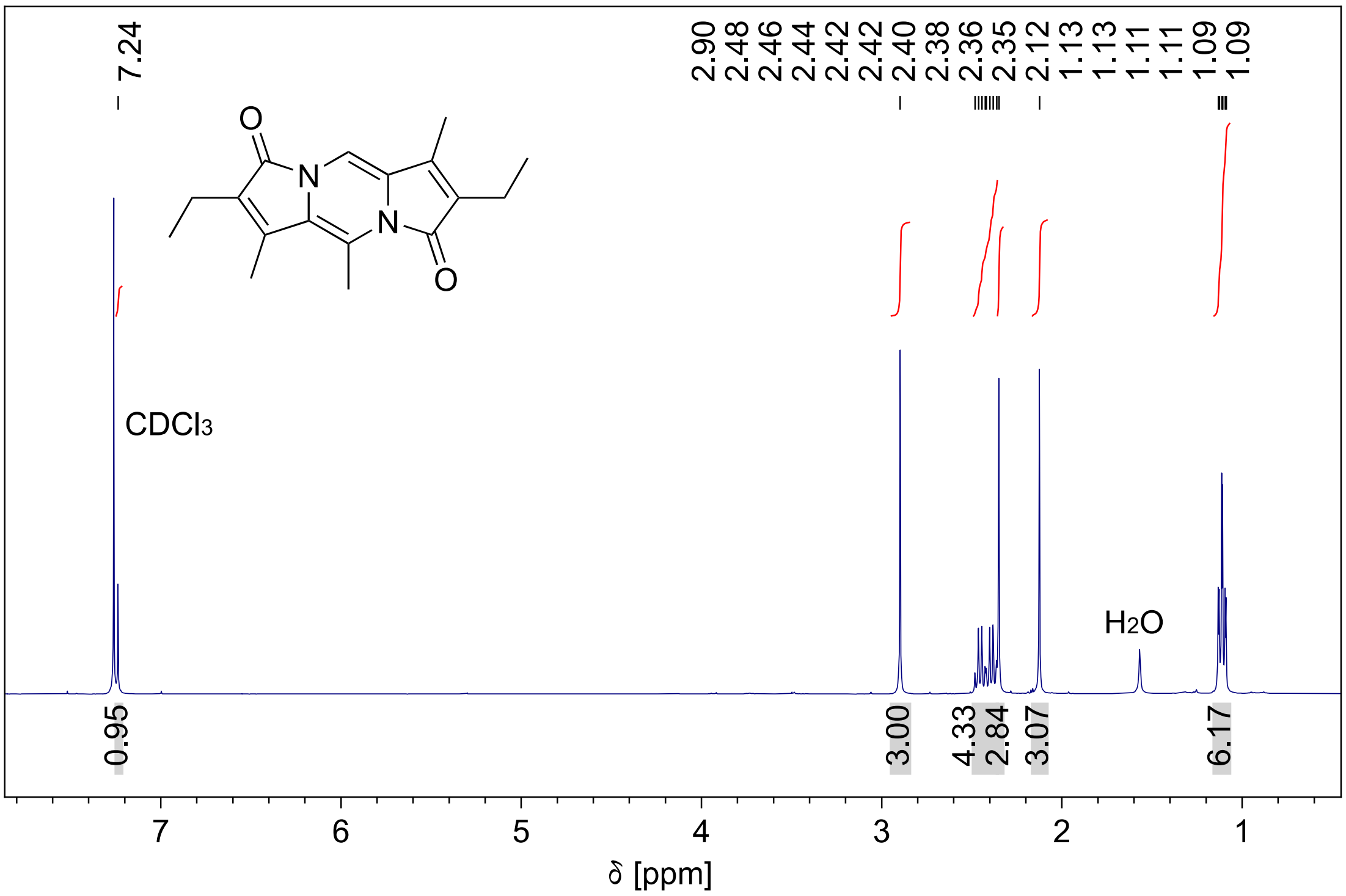

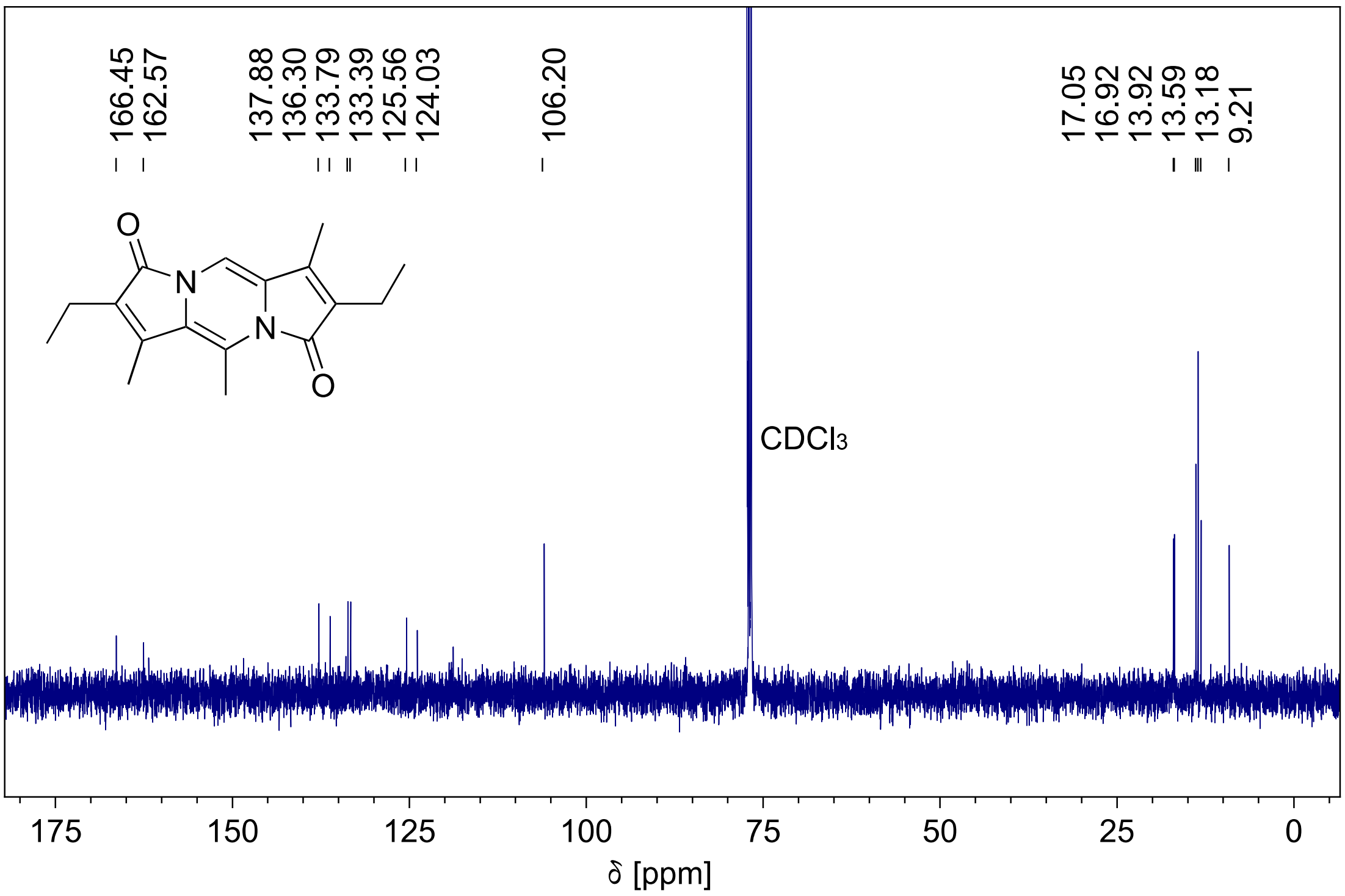

Figure S41 (*Top*) ^{1}H NMR spectrum of isopyrocoll **4a**, $CDCl_3$, 400 MHz, 298 K. *(Bottom)* ^{13}C NMR spectrum of isopyrocoll **4a**, $CDCl_3$, 101 MHz, 298 K.

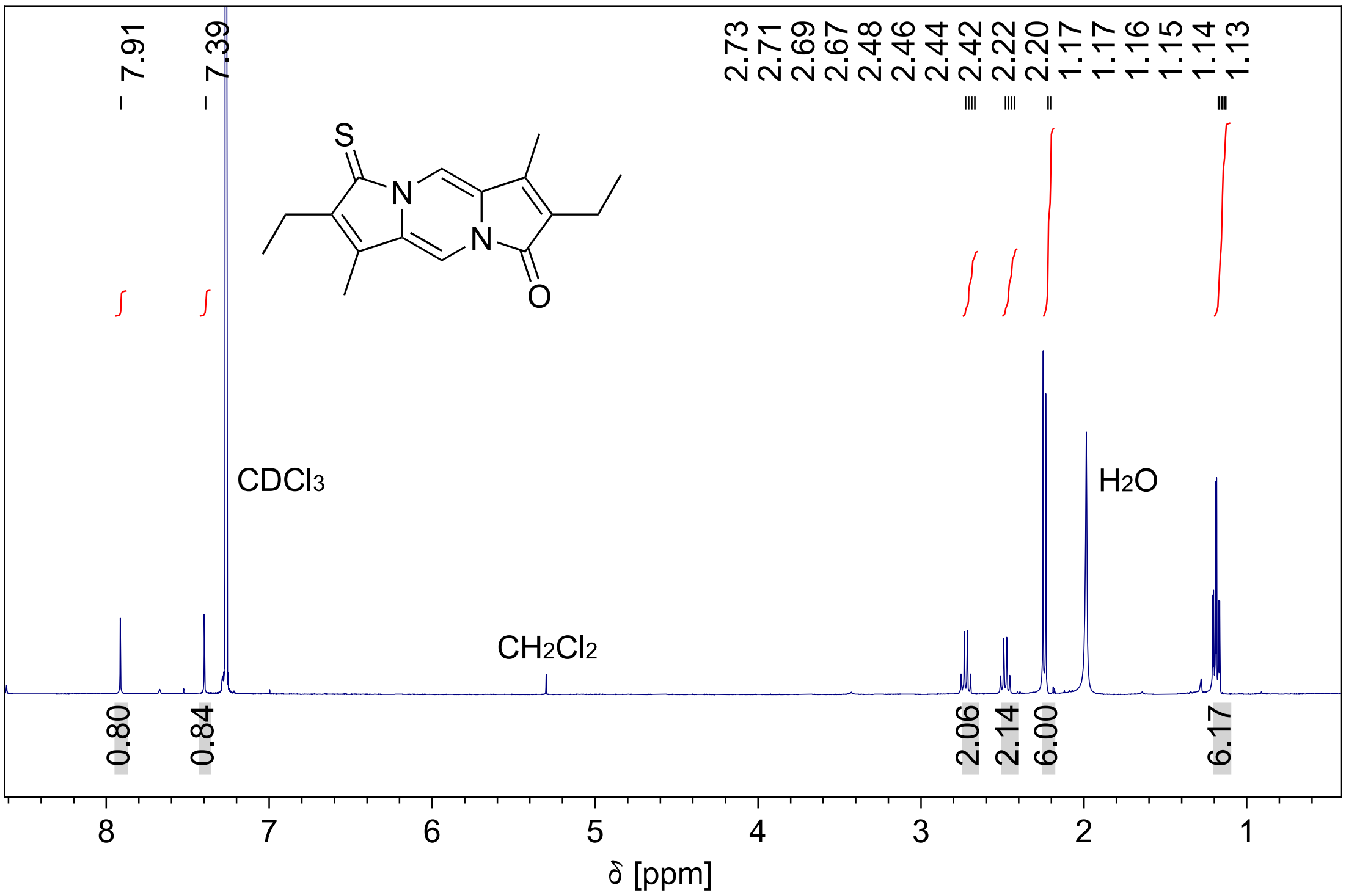

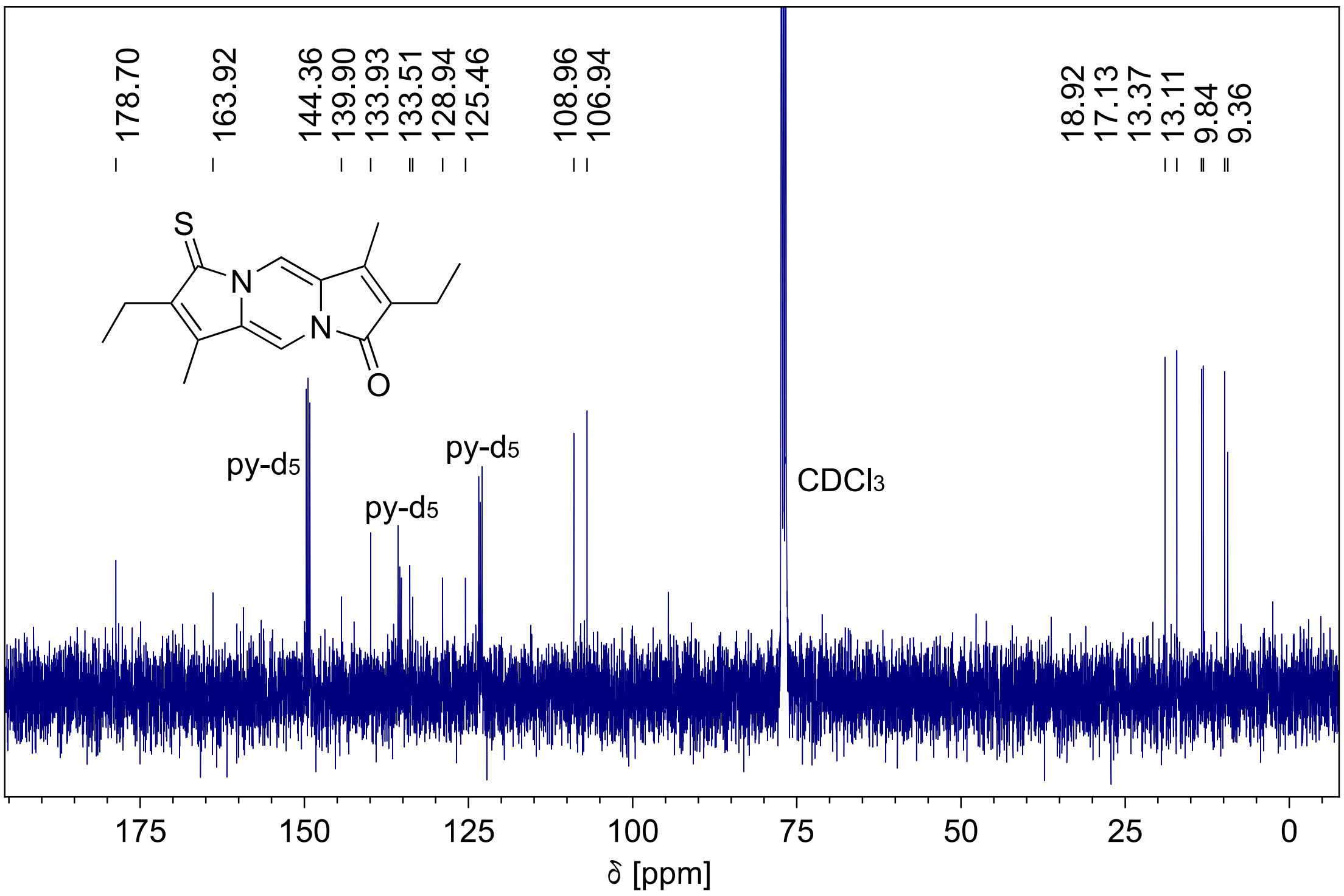

Figure S42 (*Top*) ^{1}H NMR spectrum of thionated isopyrocoll **4c**, $CDCl_3$ + 1% pyridine-d_5, 400 MHz, 298 K. *(Bottom)* ^{13}C NMR spectrum of thionated isopyrocoll **4c**, $CDCl_3$ + 1% pyridine-d_5, 101 MHz, 298 K.

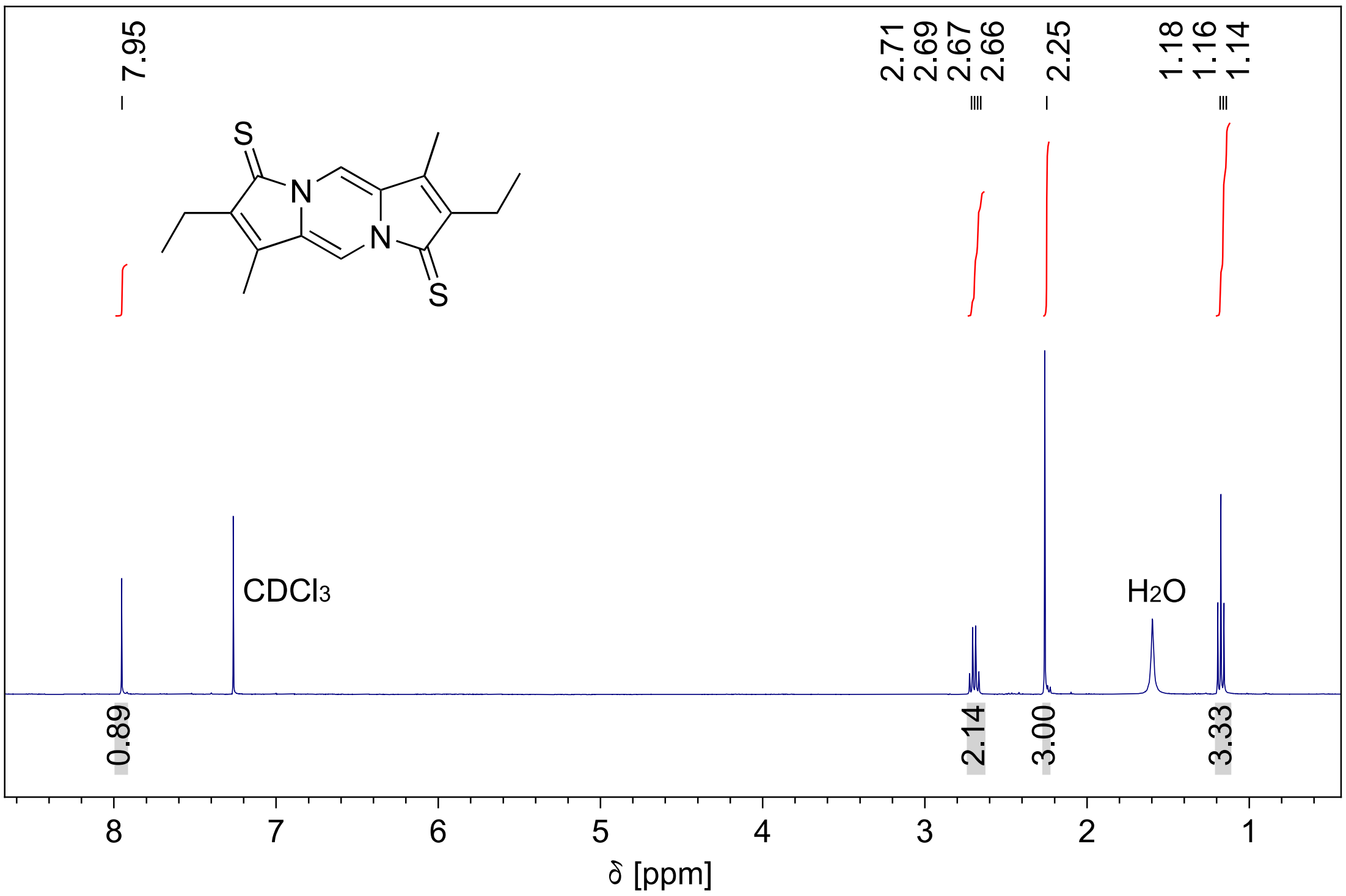

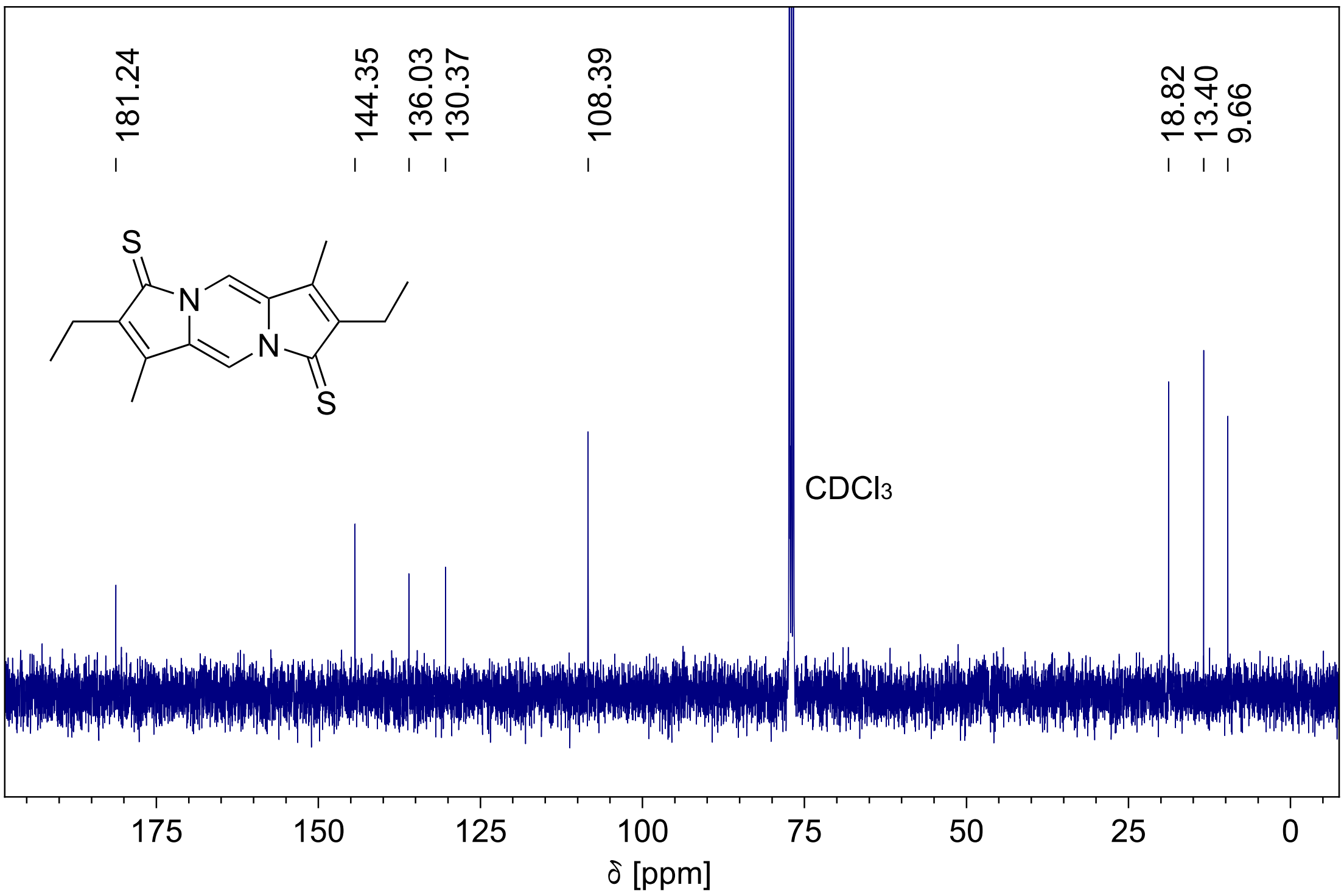

Figure S43 (*Top*) ^{1}H NMR spectrum of thionated isopyrocoll **4d**, $CDCl_3$, 400 MHz, 298 K. *(Bottom)* ^{13}C NMR spectrum of thionated isopyrocoll **4d**, $CDCl_3$, 101 MHz, 298 K.

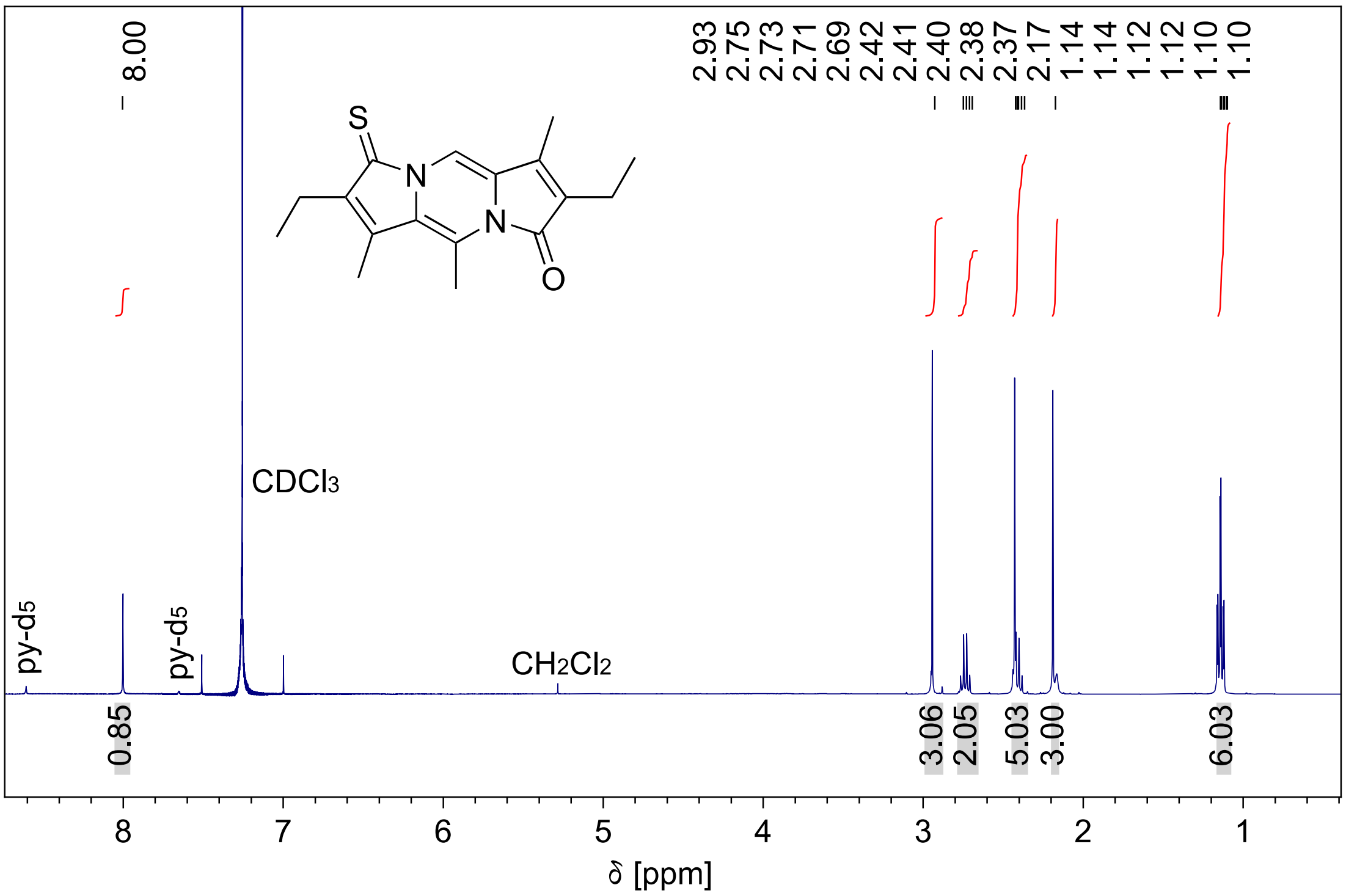

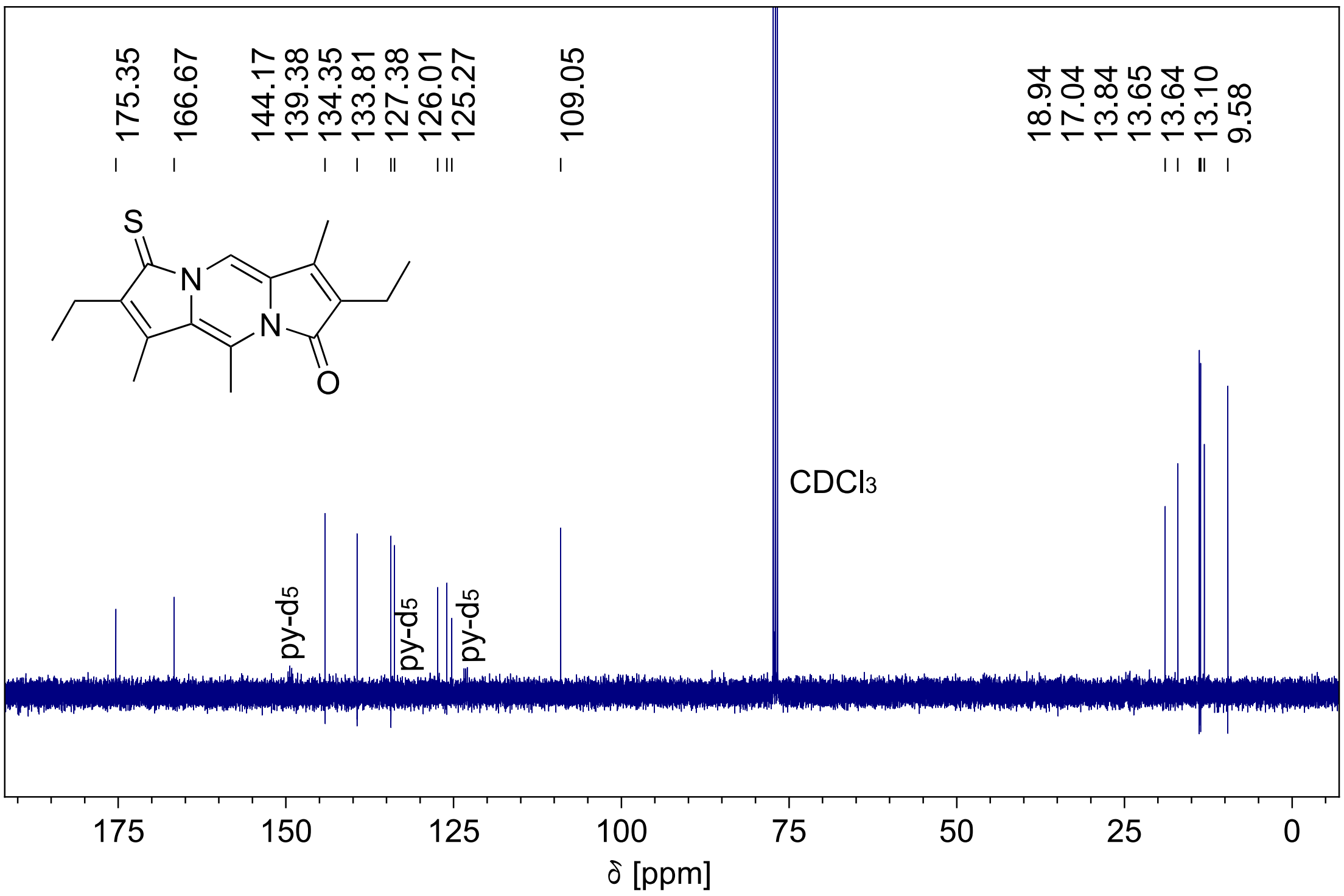

Figure S44 (*Top*) ^{1}H NMR spectrum of thionated isopyrocoll **4e**, $CDCl_3$ + 1% pyridine-d_5, 400 MHz, 298 K. *(Bottom)* ^{13}C NMR spectrum of thionated isopyrocoll **4e**, $CDCl_3$ + 1% pyridine-d_5, 101 MHz, 298 K. Note that the ^{13}C peaks at 13.64 and 13.65 ppm are very close to each other and Gaussian multiplication has been used to resolve them.

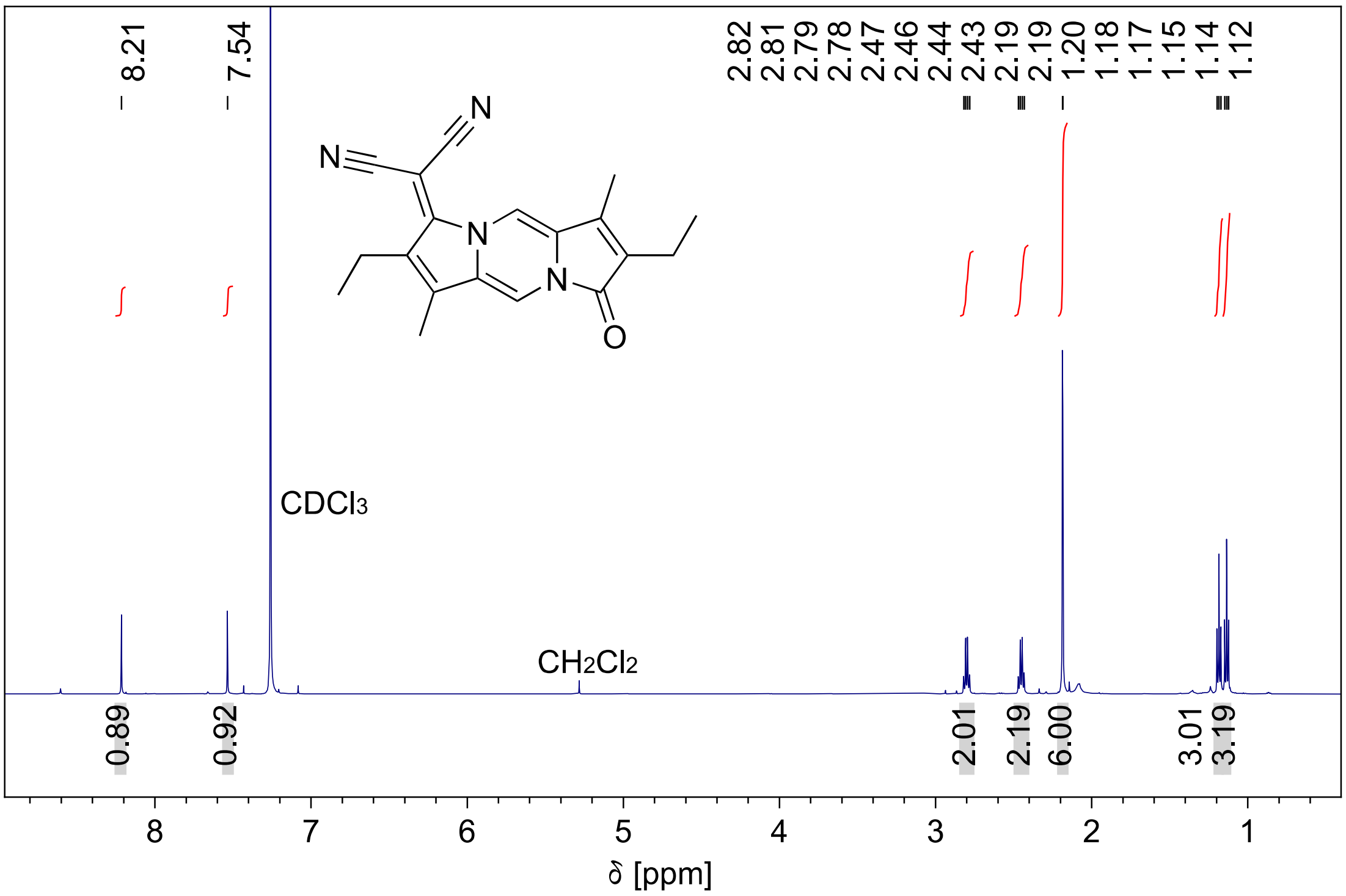

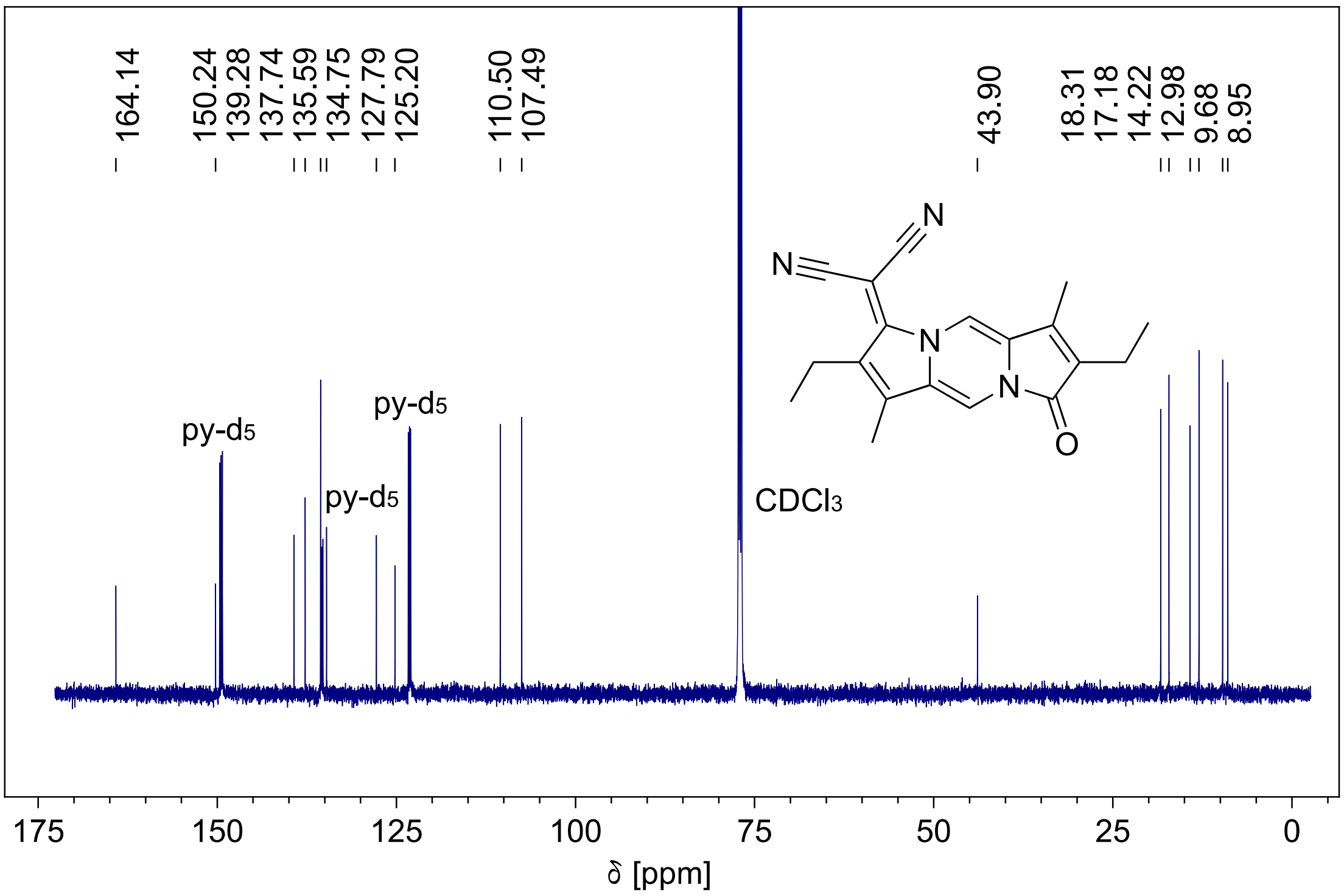

Figure S45 (*Top*) ^{1}H NMR spectrum of dicyanomethylene derivative **4f**, $CDCl_3$ + 1% pyridine-d_5, 400 MHz, 298 K. *(Bottom)* ^{13}C NMR spectrum of dicyanomethylene derivative **4f**, $CDCl_3$ + 1% pyridine-d_5, 151 MHz, 298 K.

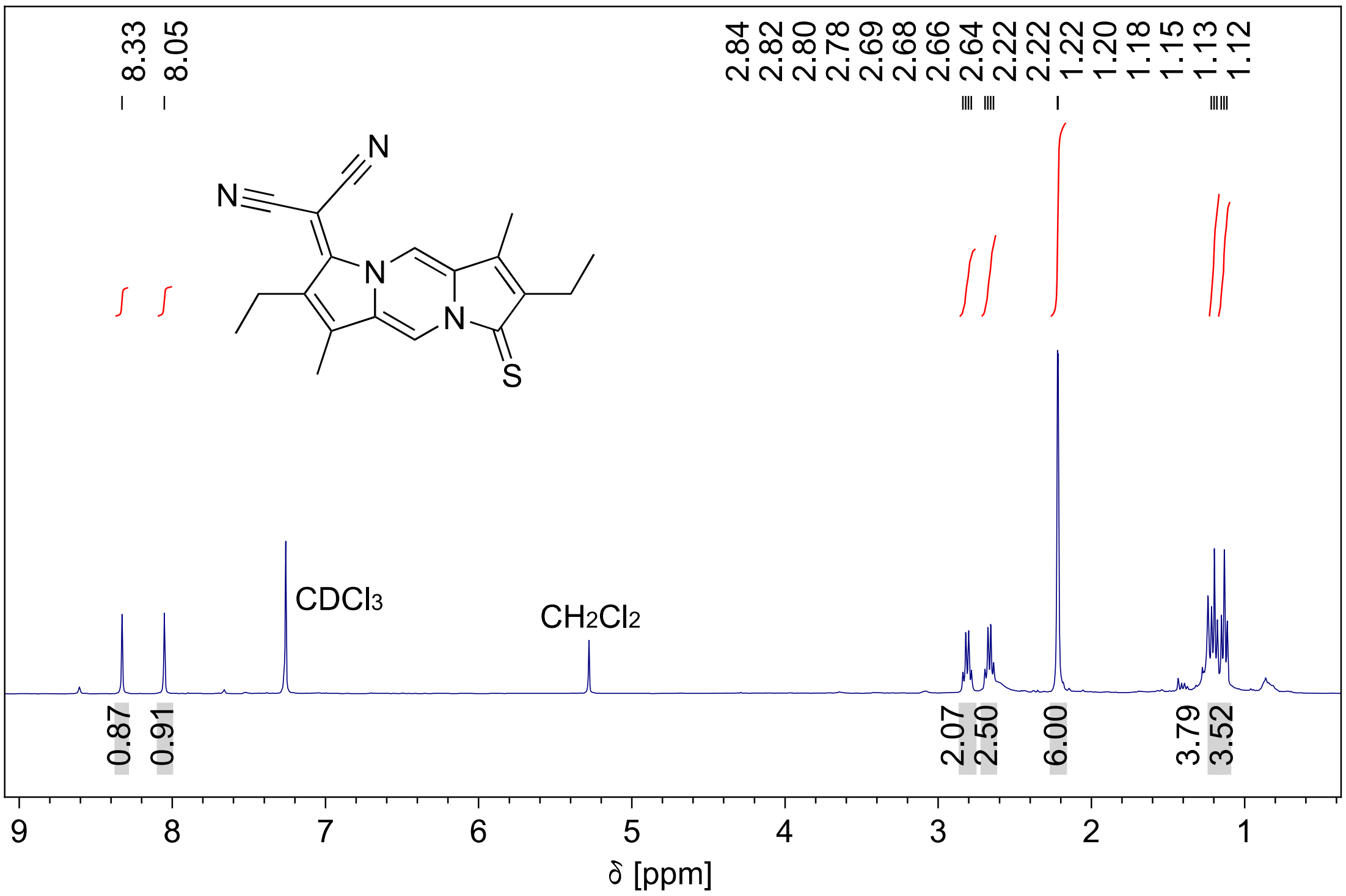

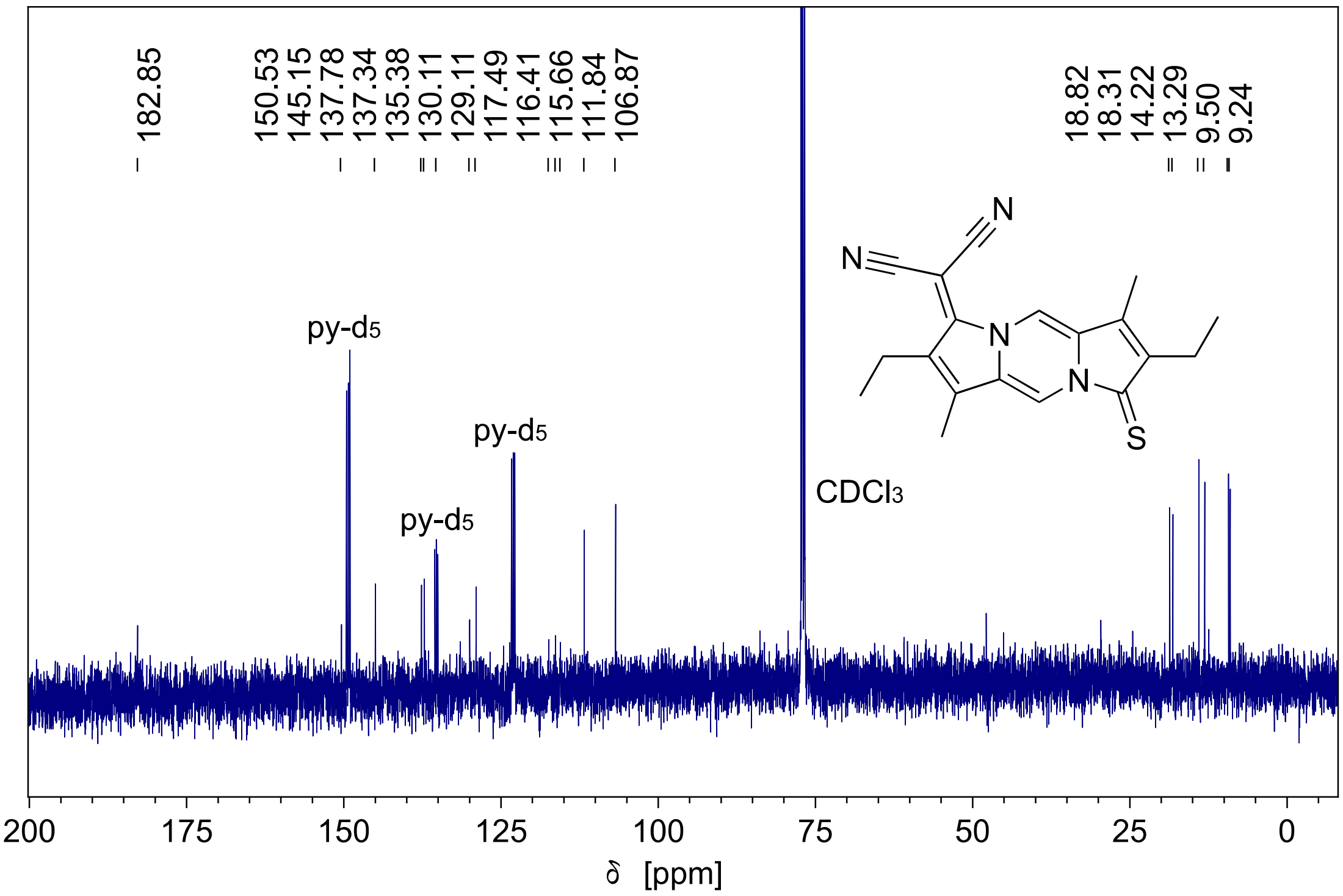

Figure S46 (*Top*) ^{1}H NMR spectrum of dicyanomethylene derivative **4g**, $CDCl_3$ + 1% pyridine-d_5, 400 MHz, 298 K. *(Bottom)* ^{13}C NMR spectrum of dicyanomethylene derivative **4g**, $CDCl_3$ + 1% pyridine-d_5, 101 MHz, 298 K.

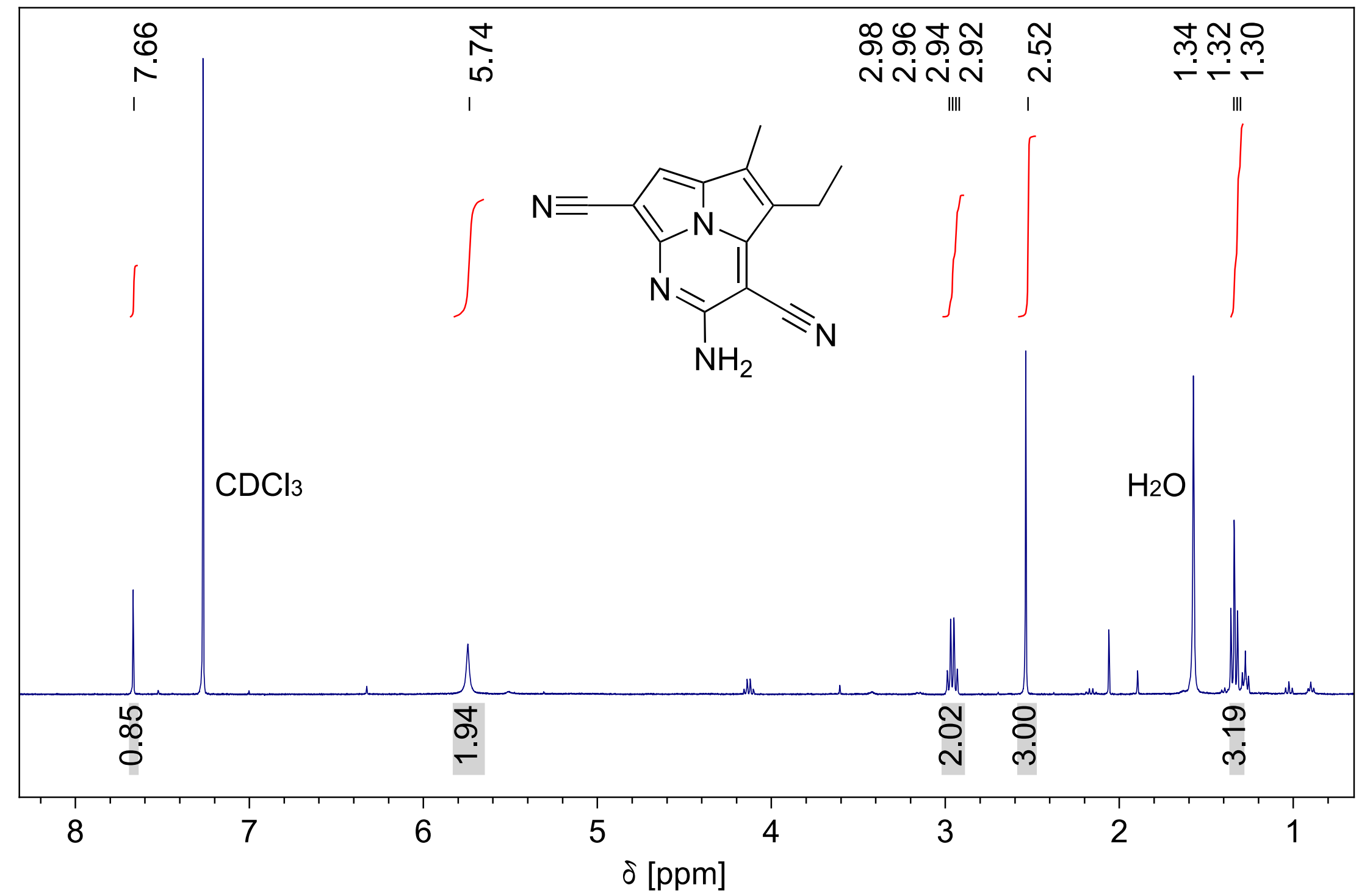

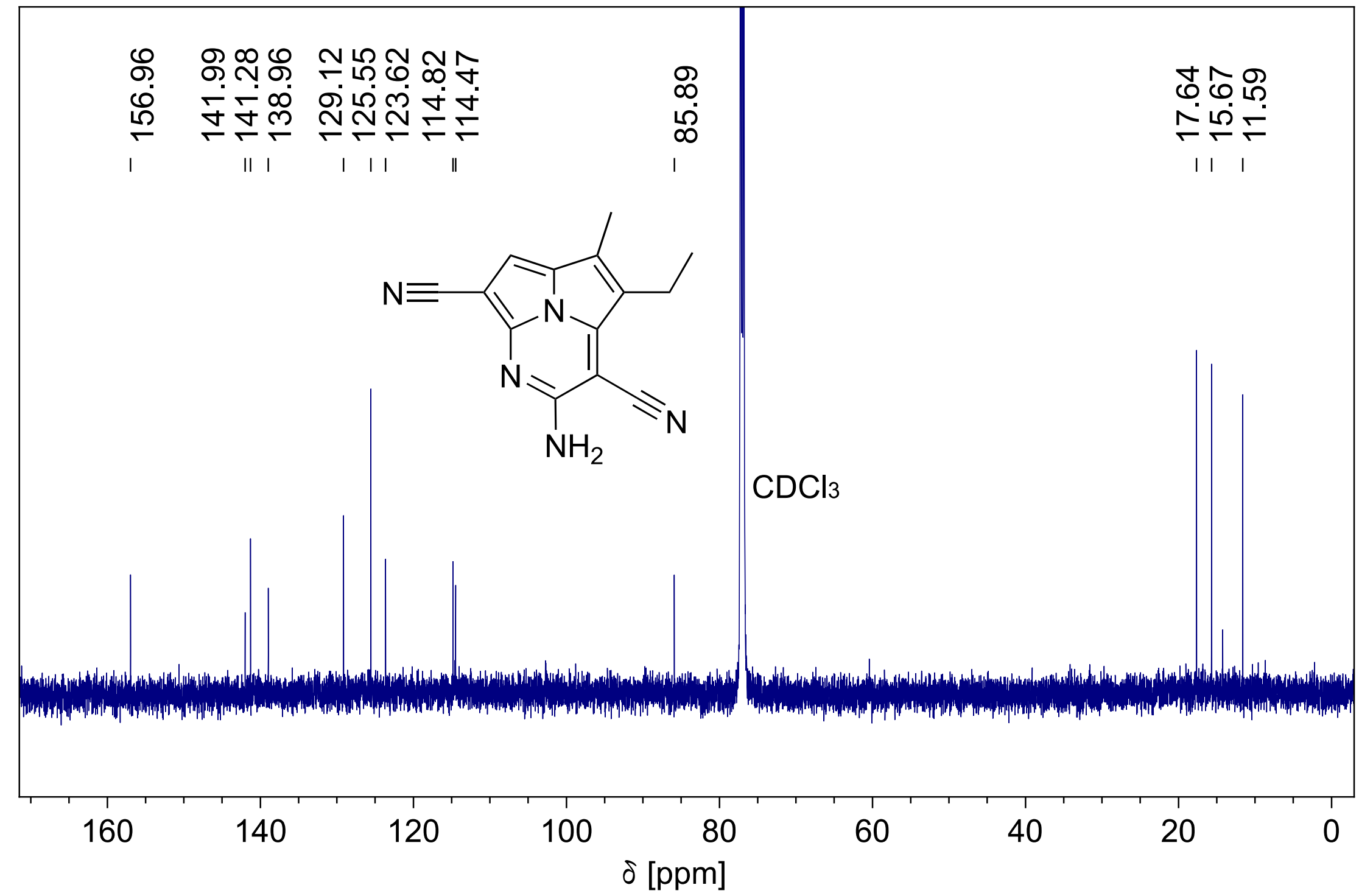

Figure S47 (*Top*) ^{1}H NMR spectrum of side product **5**, $CDCl_3$, 600 MHz, 298 K. *(Bottom)* ^{13}C NMR spectrum of side product **5**, $CDCl_3$, 151 MHz, 298 K.

S8. References

1 H. Plieninger, H. Bauer, A. R. Katritzky and U. Lerch, *Justus Liebigs Ann. Chem.*, 1962, **654**, 165–180.
2 H. Plieninger, U. Lerch and H. Sommer, *Justus Liebigs Ann. Chem.*, 1968, **711**, 130–134.
3 H. Dobeneck and E. Brunner, 1965, **340**, 200–209.
4 F. Schnierle, H. Reinhard, N. Dieter, E. Lippacher and H. von Dobeneck, *Justus Liebigs Ann. Chem.*, 1968, **715**, 90–97.
5 C. A. Wamser, *J. Am. Chem. Soc.*, 1951, **73**, 409–416.
6 C. Müller, T. Pascher, A. Eriksson, P. Chabera and J. Uhlig, *J. Phys. Chem. A*, 2022, **126**, 4087–4099.
7 M. Newville, R. Otten, A. Nelson, T. Stensitzki, A. Ingargiola, D. Allan, A. Fox, F. Carter and M. Rawlik, LMFIT Zenodo 2025.
8 Bruker AXS Inc., SAINT 2012.
9 G. M. Sheldrick, *Acta Crystallogr. Sect. C Struct. Chem.*, 2015, **71**, 3–8.
10 G. M. Sheldrick, *Acta Crystallogr. Sect. Found. Adv.*, 2015, **71**, 3–8.
11 O. V. Dolomanov, L. J. Bourhis, R. J. Gildea, J. a. K. Howard and H. Puschmann, *J. Appl. Crystallogr.*, 2009, **42**, 339–341.
12 L. J. Farrugia, *J. Appl. Crystallogr.*, 2012, **45**, 849–854.
13 C. F. Macrae, P. R. Edgington, P. McCabe, E. Pidcock, G. P. Shields, R. Taylor, M. Towler and J. van de Streek, *J. Appl. Crystallogr.*, 2006, **39**, 453–457.
14 E. F. Pettersen, T. D. Goddard, C. C. Huang, G. S. Couch, D. M. Greenblatt, E. C. Meng and T. E. Ferrin, *J. Comput. Chem.*, 2004, **25**, 1605–1612.
15 M. J. Frisch, G. W. Trucks, H. B. Schlegel, G. E. Scuseria, M. A. Robb, J. R. Cheeseman, G. Scalmani, V. Barone, G. A. Petersson, H. Nakatsuji, X. Li, M. Caricato, A. V. Marenich, J. Bloino, B. G. Janesko, R. Gomperts, B. Mennucci, H. P. Hratchian, J. V. Ortiz, A. F. Izmaylov, J. L. Sonnenberg, D. Williams-Young, F. Ding, F. Lipparini, F. Egidi, J. Goings, B. Peng, A. Petrone, T. Henderson, D. Ranasinghe, V. G. Zakrzewski, J. Gao, N. Rega, G. Zheng, W. Liang, M. Hada, M. Ehara, K. Toyota, R. Fukuda, J. Hasegawa, M. Ishida, T. Nakajima, Y. Honda, O. Kitao, H. Nakai, T. Vreven, K. Throssell, J. A. Montgomery Jr., J. E. Peralta, F. Ogliaro, M. J. Bearpark, J. J. Heyd, E. N. Brothers, K. N. Kudin, V. N. Staroverov, T. A. Keith, R. Kobayashi, J. Normand, K. Raghavachari, A. P. Rendell, J. C. Burant, S. S. Iyengar, J. Tomasi, M. Cossi, J. M. Millam, M. Klene, C. Adamo, R. Cammi, J. W. Ochterski, R. L. Martin, K. Morokuma, O. Farkas, J. B. Foresman and D. J. Fox, Gaussian 16 Revision C.01 Gaussian Inc., Wallingford CT 2016.
16 C. Bannwarth, S. Ehlert and S. Grimme, *J. Chem. Theory Comput.*, 2019, **15**, 1652–1671.
17 A. D. McLean and G. S. Chandler, *J. Chem. Phys.*, 1980, **72**, 5639–5648.
18 R. Krishnan, J. S. Binkley, R. Seeger and J. A. Pople, *J. Chem. Phys.*, 1980, **72**, 650–654.
19 A. D. Becke, *J. Chem. Phys.*, 1993, **98**, 1372–1377.
20 E. D. Glendening, J. K. Badenhoop, A. E. Reed, J. E. Carpenter, J. A. Bohmann, C. M. Morales, P. Karafiloglou, C. R. Landis and F. Weinhold, NBO 7.0 Theoretical Chemistry Institute, University of Wisconsin, Madison 2018.
21 E. D. Glendening, C. R. Landis and F. Weinhold, *J. Comput. Chem.*, 2019, **40**, 2234–2241.
22 D. W. Szczepanik, M. Andrzejak, K. Dyduch, E. Żak, M. Makowski, G. Mazur and J. Mrozek, *Phys. Chem. Chem. Phys.*, 2014, **16**, 20514–20523.
23 D. W. Szczepanik, *Comput. Theor. Chem.*, 2016, **1080**, 33–37.
24 D. W. Szczepanik, M. Andrzejak, J. Dominikowska, B. Pawełek, T. M. Krygowski, H. Szatylowicz and M. Solà, *Phys. Chem. Chem. Phys.*, 2017, **19**, 28970–28981.
25 R Core Team, R: A Language and Environment for Statistical Computing. R Foundation for Statistical Computing, Vienna, Austria 2025.

26 C. Adamo and V. Barone, *J. Chem. Phys.*, 1999, **110**, 6158–6170.
27 J. Tao, J. P. Perdew, V. N. Staroverov and G. E. Scuseria, *Phys. Rev. Lett.*, 2003, **91**, 146401.
28 V. N. Staroverov, G. E. Scuseria, J. Tao and J. P. Perdew, *J. Chem. Phys.*, 2004, **121**, 11507.
29 J.-D. Chai and M. Head-Gordon, *Phys. Chem. Chem. Phys.*, 2008, **10**, 6615–6620.
30 T. H. Dunning Jr., *J. Chem. Phys.*, 1989, **90**, 1007–1023.
31 F. Weigend and R. Ahlrichs, *Phys. Chem. Chem. Phys.*, 2005, **7**, 3297–3305.
32 J. Tomasi, B. Mennucci and R. Cammi, *Chem. Rev.*, 2005, **105**, 2999–3094.
33 J. R. Cheeseman, G. W. Trucks, T. A. Keith and M. J. Frisch, *J. Chem. Phys.*, 1996, **104**, 5497–5509.
34 D. Jacquemin, E. A. Perpète, I. Ciofini and C. Adamo, *J. Chem. Theory Comput.*, 2010, **6**, 1532–1537.
35 O. El Bakouri, J. R. Smith and H. Ottosson, *J. Am. Chem. Soc.*, 2020, **142**, 5602–5617.
36 T. Zeng, R. Hoffmann and N. Ananth, *J. Am. Chem. Soc.*, 2014, **136**, 5755–5764.
37 M. J. Y. Tayebjee, K. L. K. Lee and T. W. Schmidt, *J. Mater. Chem. C*, 2024, **12**, 13884–13891.
38 M. B. Smith and J. Michl, *Chem. Rev.*, 2010, **110**, 6891–6936.
39 A. Stanger, *J. Org. Chem.*, 2010, **75**, 2281–2288.
40 A. Rahalkar and A. Stanger, AROMA package http://chemistry.technion.ac.il/members/amnon-stanger 2014.
41 R. Gershoni-Poranne and A. Stanger, *Chem. – Eur. J.*, 2014, **20**, 5673–5688.
